# Full (Electro-)Chemical Control over the Aggregation State of Diblock Copolymer Mixtures Including Nonequilibrium Micelles

**DOI:** 10.1021/acspolymersau.6c00044

**Published:** 2026-06-01

**Authors:** Vincent Schildknecht, Annika Lehmann, Richard Neubert, Quirin Prasser, Anja Voigt, Gilbert Chahine, Isabelle Morfin, Erica Brendler, Sabine Rosenfeldt, Ulrich Mansfeld, Felix A. Plamper

**Affiliations:** † Institute of Physical Chemistry, 26545 TU Bergakademie Freiberg, Lessingstr. 45, Freiberg 09599, Germany; ‡ CNRS, Grenoble-INP, SIMAP, Université Grenoble Alpes, Grenoble 38000, France; § 27015 CNRS, LiPhy, Université Grenoble Alpes, Grenoble 38000, France; ∥ Institute of Analytical Chemistry, TU Bergakademie Freiberg, Lessingstr. 45, Freiberg 09599, Germany; ⊥ Bayreuth Institute of Physical Chemistry I and Bavarian Polymer Institute (BPI), 26523 University of Bayreuth, Universitätsstr. 30, Bayreuth 95447, Germany; # Bayreuth Institute for Macromolecular Research (BIMF) and Bavarian Polymer Institute (BPI), 26523 University of Bayreuth, Universitätsstr. 30, Bayreuth 95447, Germany; ∇ Center for Efficient High Temperature Processes and Materials Conversion ZeHS, TU Bergakademie Freiberg, Winklerstr. 5, Freiberg 09599, Germany; ○ Freiberg Center for Water Research ZeWaF, TU Bergakademie Freiberg, Winklerstr. 5, Freiberg 09599, Germany

**Keywords:** formulation, surfactants, amphiphiles, electrolysis, polyelectrolytes, pathway-dependence

## Abstract

Stimuli-responsive polymers have endowed soft matter research with numerous applications. Moving beyond classical stimuli like temperature or pH, we introduce an electrochemical approach to control block copolymer aggregation by shifting between bishydrophilic and amphiphilic states. The aqueous solutions of the nonionic-cationic block copolymers poly­[*N*-(3-aminopropyl)­methacrylamide]_30_-*b*-poly­(*N*,*N*-dimethylacrylamide)_120_ (PAPMA_30_-*b*-PDMAA_120_), synthesized via RAFT polymerization, and poly­(ethylene oxide)_114_-*b*-poly­{[2-(methacryloyloxy)­ethyl]­diisopropylmethylammonium chloride}_171_ (i.e., PEO_114_-*b*-PDPAEMA_171_ with a quaternized poly­(diisopropylaminoethyl methacrylate) block, abbreviated as qPDPAEMA), were investigated for micellization via Dynamic Light Scattering (DLS), Small Angle X-ray Scattering (SAXS), and Cryogenic Transmission Electron Microscopy (cryo-TEM). Both polymers exhibit selective aggregation with hexacyanoferrate species: PAPMA-*b*-PDMAA forms aggregates specifically in the presence of ferrocyanide ions ([Fe­(CN)_6_]^4–^), while PEO-*b*-qPDPAEMA forms micelles with ferricyanide ions ([Fe­(CN)_6_]^3–^). These polymer systems demonstrate both chemical and electrochemical reversibility, with the combined polymer system allowing selective control over the type of polymer that aggregates by modulating the ferricyanide/ferrocyanide ratio. Furthermore, the pH sensitivity of PAPMA-*b*-PDMAA allows the disintegration of the micellar state by increasing the pH (electro-)­chemically. In total, these results demonstrate full control over the assembly state of complex polymer mixtures by electrochemical means. In addition, it is shown for PEO-*b*-qPDPAEMA that electrochemical switching as a soft trigger leads to the thermodynamically preferred micelle morphology, while rapid chemical redox leads to kinetically trapped, nonequilibrium micelles. Here, electrochemistry can act as a trigger to transform the trapped micelles toward the thermodynamically stable ones, which can be recycled back to the nonequilibrium micelles. The principle of this electrochemically triggered micellization could represent a significant step toward developing stimuli-sensitive materials for autonomous drug delivery, sensing, or even smart materials within a systems chemistry approach.

## Graphical abstract

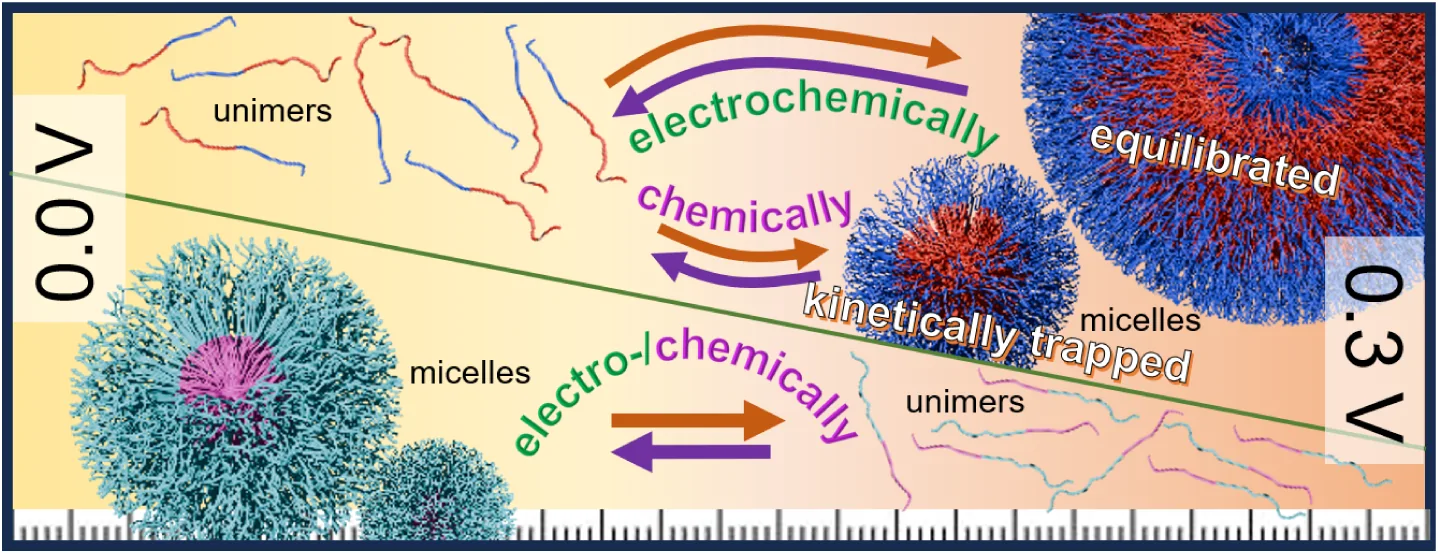

## Introduction

The precise response of molecules to certain signals is a prerequisite for systems chemistry,^1^ including time-programmed assemblies.^2^ Here, stimuli-responsive block copolymers have emerged as a powerful tool capable of undergoing controlled structural transitions in response to external triggers such as pH^3^ or temperature.^4^ Particularly intriguing are polymers where a solubility change in one or more blocks induces a structural transition. These macromolecules could turn amphiphilic and self-assemble^5^ on demand into various morphologies, including star-like micelles, vesicles,^6^ and other organized structures,^7^ making them highly attractive for a broad spectrum of applications,^8^ e.g., in drug delivery,^9^ sensing,^10^ and smart material systems.

While pH and temperature changes offer reliable triggers for polymer transitions, they often lack the precision required for more sophisticated applications. Thus, among the various stimuli, electrochemical control^11^ offers unique advantages because of its precise temporal and spatial control, reversibility, and compatibility with microfluidic and biomedical devices. Electrochemical control beneficially lacks a direct feeding of chemicals, while the solution behavior in, e.g., a reaction vessel can be manipulated from the outside. Thinking of microrobots,^12^ their sensing and manipulating abilities could induce an autonomous response to their environment with even further feedback loops. Hence, electrochemically addressable polymer systems could pave the way to such sophisticated system chemistry approaches with novel emerging behaviors. Such addressability can be achieved by covalently incorporating electroactive groups into the polymer,^13^ including ferrocene and photoresponsive moieties that drive vesicle-to-micelle transitions in di- and triblock copolymers,^14,15^ or by harnessing other molecules as triggers.

Our current approach relies on polyelectrolytes, i.e., polymers bearing electroactive groups in their repeating units. Depending on their pH-dependent charging behavior, they are strategically classified as either weak or strong polyelectrolytes in the case of pH-dependently or pH-independently charged monomeric units, respectively. Polyelectrolytic block copolymers exhibit rich aggregation behavior because of their electrostatic interactions with counterions and other charged species, forming polyelectrolyte or interpolyelectrolyte complexes.^16^ Furthermore, the complexation of polyelectrolyte blocks with multivalent ions can dramatically alter the hydrophilic–hydrophobic balance of the copolymer, triggering different conformational changes. Examples of different multivalent ions in that context include, e.g., hexacyanocobaltate­(III),^17^ chromate,^18^ yttrium^3+^ ions,^19^ or diverse other heavy metals.^20^

When these interactions involve redox-active species, such as hexacyanoferrate ions, the aggregation state can be reversibly controlled through electrochemical oxidation and reduction. The use of hexacyanoferrates as redox probes and as complexants for miktoarm star polymers with poly­[[2-(methacryloyloxy)­ethyl]­trimethylammonium chloride] (PMOTAC) as the responsive moiety was introduced by our group.^21^ PMOTAC turns insoluble in the presence of hexacyanoferrate­(III) ions (ferricyanide) but remains soluble in the presence of hexacyanoferrate­(II) ions (ferrocyanide). In our case, we chose a similar polymer to PMOTAC, carrying isopropyl groups instead of methyl groups at the quaternary nitrogen atom. This polymer, namely poly­{[2-(methacryloyloxy)­ethyl]­diisopropylmethylammonium chloride}, or quaternized poly­(diisopropylaminoethyl methacrylate) (qPDPAEMA), appears more hydrophobic^22,23^ than its methyl-carrying derivative, and the strong interaction with ferricyanide was already shown.^23^ Molecular-level evidence for this selective ferricyanide binding to the quaternary ammonium groups was recently provided for MAPTAC-based microgels, where paramagnetic relaxation enhancement NMR (nuclear magnetic resonance spectroscopy) revealed pseudocontact shifts as the dominant mechanism,^24^ an interaction that is directly transferable to our qPDPAEMA system. Finally, poly­(ethylene oxide) (PEO), also known as poly­(ethylene glycol), is introduced as a solubilizing block, which is always water-soluble under the conditions used (room temperature).^25^

In real life, surfactant systems with just one amphiphilic component are rarely used. In most cases, mixtures of different surfactants and auxiliary additives act concertedly, as seen in washing powder, shampoo, cosmetics, or pharmaceuticals. Such complex formulations are often needed for fine-tuning to serve optimally a specific purpose.^26–28^ Then, stimuli-responsive components could broaden the application scope of a product, while an antagonistic action of the different ingredients or a changing role of certain amphiphiles could be beneficial in these cases. However, such advanced, adaptive formulations are not yet available.

Academically, fascinating structures have been obtained from amphiphilic polymer mixtures. For example, block copolymer mixtures assemble into unprecedented micellar morphologies, as demonstrated by Winnik, Manners, and coworkers.^29–31^ The coassembly of individual polymers into mixed micelles was also utilized for the generation of, e.g., interpolyelectrolyte complex micelles.^16,32,33^ Here, nonequilibrium processes could be investigated,^34^ whereby these polyelectrolyte complexes are valuable constituents of kinetically trapped nonequilibrium micelles,^35^ which can undergo a property change under basically constant conditions by the application of a temporary trigger,^36–38^ such as temperature. Below, we will demonstrate an extension of this approach toward an electrochemical trigger. Regarding simple amphiphile mixtures, a comicellization into mixed micelles can occur, or an independent micellization of each of the components can be achieved with the help of preassembled frozen micelles^39^ or by the use of incompatible solvophobic blocks. Also, more complex co- and self-assembly schemes, depending on the molecular structure and the presence of additives, were examined.^40–42^ However, the individual control over the assembly state of these copolymer mixtures, in terms of micellized or unimolecularly dissolved molecules for each component, is more difficult to achieve. One could think of a combination of two diblock copolymers containing oppositely charged weak polyelectrolytes, where one component forms micelles at low pH and the other one forms micelles at high pH,^43^ similar to what is possible for schizophrenic micelles. However, a fully dissolved state is difficult to obtain in this approach, as the system is likely limited by hydrogen bonding interactions preventing complete solubility. Achieving full control, where either all polymers form micelles simultaneously, all are unimolecularly dissolved, or only one of the polymers forms micelles, while the other one is dissolved (and vice versa), is even more difficult and would require a second orthogonal trigger, such as temperature or light. In this publication, we will introduce a polymer system that accomplishes exactly this task with only one trigger. Even more, control can be achieved by purely electrochemical means.

Therefore, we now introduce an orthogonally switchable system by adding another different amphiphilic block copolymer to the previously discussed PEO-*b*-qPDPAEMA system, which now interacts preferentially with ferrocyanide instead of ferricyanide. For this study, we chose poly­[*N*-(3-aminopropyl)­methacrylamide] (PAPMA) because previous investigations^44^ suggested enhanced interaction of the polymer with ferrocyanide at the electrode surface. The interaction of a protonated primary amino group with ferrocyanide in the context of electroactive aggregation of polymers was also investigated by Matsuno et al.^45^ They employed poly­(allylamine-*co*-allylurea) (PAU) as the polymer component for electrochemically reversible ferrocyanide complexation. The specific interaction between the amine and ferrocyanide over ferricyanide can be explained by their reduced polarizability in line with the Pearson hard/soft acid/base concept,^46^ which is discussed more thoroughly in the Results and Discussion section.

To facilitate this orthogonal switching utilizing PAPMA, a novel block copolymer consisting of the polyelectrolyte PAPMA and the hydrophilic polymer poly­(*N*,*N*-dimethylacrylamide) (PDMAA) was synthesized. PDMAA is a well-known biocompatible hydrophilic polymer that is already used in multiple applications, for example, in the medical field for contact lenses^47^ or drug delivery.^48^ DMAA is often preferred compared to the alternative acrylamide because of the toxicity of the latter. After presenting the route of synthesis, we relate its solution properties to those of already prepared PEO-*b*-qPDPAEMA. We pay special attention to their behavior in the presence of hexacyanoferrates, where redox reactions can occur chemically or electrochemically, with implications on the equilibrium situation. Finally, we will address mixtures of both polymers and demonstrate their independent switchability by electrochemistry.

## Results and Discussion

### Synthesis of PAPMA_30_-*b*-PDMAA_120_

While there are a number of reports on copolymers made from the monomers *N*,*N*-dimethylacrylamide (DMAA) and *N*-(3-aminopropyl)­methacrylamide (APMA),^49–54^ readily prepared block copolymers of these two monomers have, to the best of our knowledge and despite the availability of the involved monomers, not been reported so far. Hence, a novel block copolymer, poly­[*N*-(3-aminopropyl)­methacrylamide]_30_-*b*-poly­(*N*,*N*-dimethylacrylamide)_120_ (PAPMA_30_-*b*-PDMAA_120_), was synthesized in three steps as shown in Figure 1. The synthesis of the pure diblock copolymer appeared being nontrivial, showing obstacles both in the blocking efficiency and the availability of analytical tools: an effective block copolymerization of this secondary methacrylamide/tertiary acrylamide pair is not straightforward due to an unfavorable blocking efficiency in both polymerization directions probably similar to the reasons for the ineffective homopolymerization of dialkylmethacrylamides.^55^ In addition, the analysis of the resulting block copolymer is challenging in terms of size exclusion chromatography (SEC) because of the subtle interplay of the different solubilities of the homopolymers in typical eluents,^56^ which makes protected derivatives the primary workaround for the characterization of these polymers (details see Section 2.1 in SI). To obtain a polymer with a narrow molecular weight distribution and controllable chain length, controlled radical polymerization, in particular reversible addition−fragmentation chain-transfer (RAFT) polymerization, was chosen. As chain transfer agent (CTA), 2-cyano-2-propyldodecyltrithiocarbonate (CPDC) leads to a successful polymerization (**A**) of the monomer *N*-(3-*t*-Boc-aminopropyl)­methacrylamide, i.e. APMA bearing a *tert*-butyloxycarbonyl (*t*-Boc) protecting group, in ethanol. The second block consisting of *N*,*N*-dimethylacrylamide was also added in ethanol using a similar procedure, resulting in the diblock copolymer **B**. The final polymer (**C**) was then obtained by deprotecting the amine.

**Figure 1:**
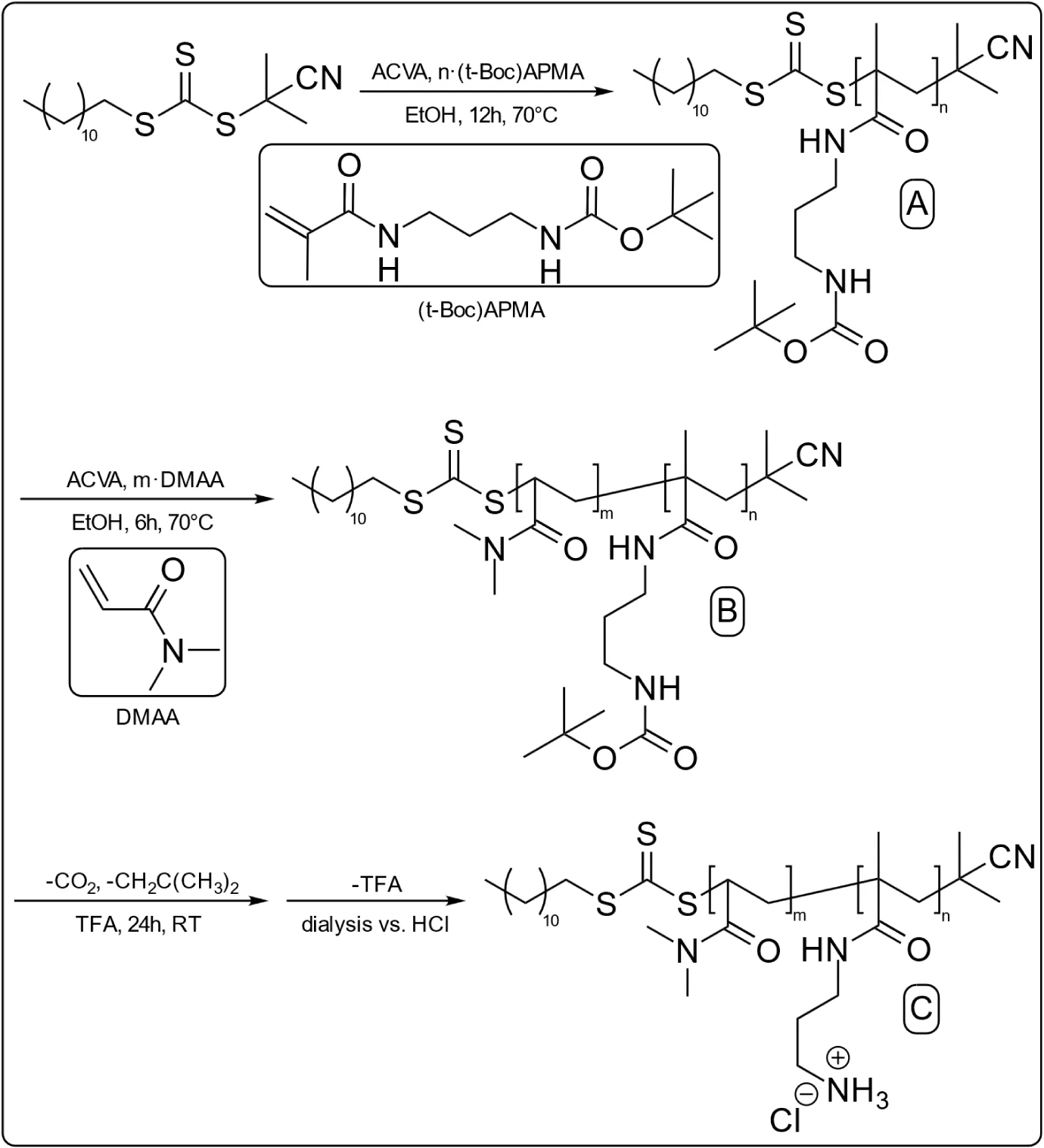
Schematic illustration of the synthesis of the novel diblock copolymer PAPMA-*b*-PDMAA (C). First step, RAFT polymerization of *N*-(3-*t*-Boc-aminopropyl)­methacrylamide (A) with CPDC (2-cyano-2-propyldodecyltrithiocarbonate) as the CTA. Second step, RAFT polymerization of *N*,*N*-dimethylacrylamide onto the macro-CTA to yield poly­[*N*-(3-*t-*Boc-aminopropyl)­methacrylamide-*b*-poly­(*N*,*N*-dimethylacrylamide). Third step, removal of the protecting *t*-Boc group with trifluoroacetic acid (TFA). For the polymerization steps, 4,4′-azobis­(4-cyanopentanoic acid) (ACVA) was used as initiator.

The SEC results of compound **A** (*Đ* = 1.23; *M*_n_ = 5080, PEO-calibrated) and **B** are depicted in Figure 2. In the first polymerization, the conversion ranged around 55% (as an average over three different attempts), indicating that system-specific optimization could unlock even greater efficiency gains.

**Figure 2:**
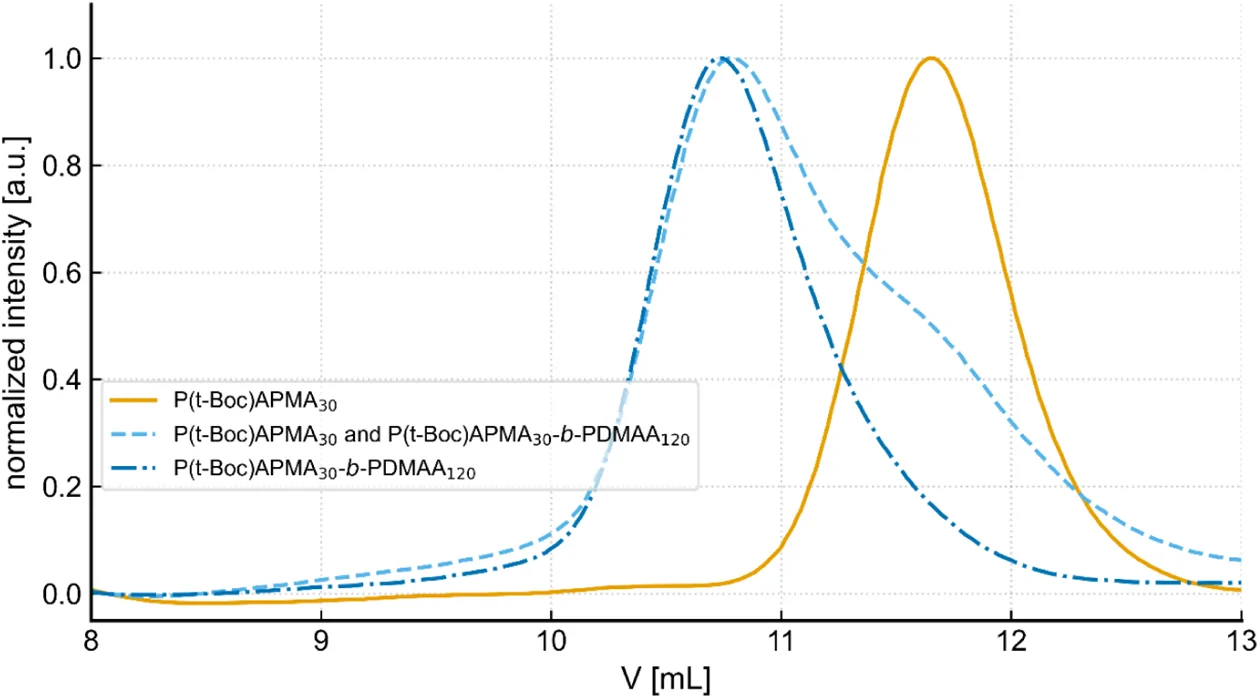
SEC of the homopolymer P­(*t*-Boc)­APMA_30_ (orange; *Đ* = 1.23; *M*_n_ = 5080, PEO-calibrated), P­(*t*-Boc)­APMA_30_-*b*-PDMAA_120_ with residual homopolymer (dashed, light blue line), and purified P­(*t*-Boc)­APMA_30_-*b*-PDMAA_120_ (doted dark blue line; *Đ* = 1.27; *M*_n_ = 14,300, PEO-calibrated). The preparation and characterization of the other block copolymer is documented in a previous publication.^23^

After the addition of the second block (94% conversion on average), a non-negligible amount of homopolymer **A** indicates a nonquantitative blocking efficiency. The homopolymer was removed by dialysis (details in Section 1.1.2 in SI), yielding the pure diblock (*Đ* = 1.27; *M*_n_ = 14,300, PEO-calibrated; see Figure 2). The reason for the reduced blocking efficiency might be attributed to the largely different reaction kinetics of the different blocks (elaborated in detail in Section 2.1 in SI).^57–59^

Detailed results from NMR for all compounds are given in the SI (Section 1.1 ^60^). Conversion and yield suggest an average chain length of *n* = *P*_n,PAPMA_ = 30 for the PAPMA block and an average chain length of *m* = *P*_n,PDMAA_ = 87 for the PDMAA block of the specific polymer used in this paper. However, it must be kept in mind that the block length of the second block is higher than anticipated from conversion and the CTA/monomer ratio due to the presence of residual P­(*t*-Boc)­APMA_30_ homopolymer after synthesis. The relative proportions of both blocks can be elucidated through NMR spectroscopy of polymers **B** (after purification) and **C**. With this approach, the PAPMA/P­(*t-*Boc)­APMA block can be assigned to a molar ratio of approximately 0.2 (details: Section 1.1.2 in SI). Assuming *n* = 30, we obtain a substantially larger *m* value of 120. End group determination is troublesome for the protected polymer, because of many overlapping signals, but it is possible for the unprotected polymers (deprotected macro-CTA and polymer **C**). This end group determination leads to *n* = 29 and *m* = 123, which aligns quite well with the results determined by the molar ratio.

Furthermore, both the final diblock copolymer PAPMA_30_-*b*-PDMAA_120_ and the deprotected macro-CTA PAPMA_30_ were analyzed by using diffusion-ordered NMR spectroscopy (DOSY) to corroborate the SEC findings. This analysis confirmed the absence of residual homopolymer and verified the successful attachment of the PDMAA block to the macro-CTA. As shown in Figure 3, the diblock copolymer exhibits a clear shift toward lower diffusion coefficients relative to those of the macro-CTA precursor, with well-aligned signals observed for both polymers. The appearance of characteristic PDMAA signals in the 2.4–2.7 ppm region further confirms successful block incorporation.

**Figure 3:**
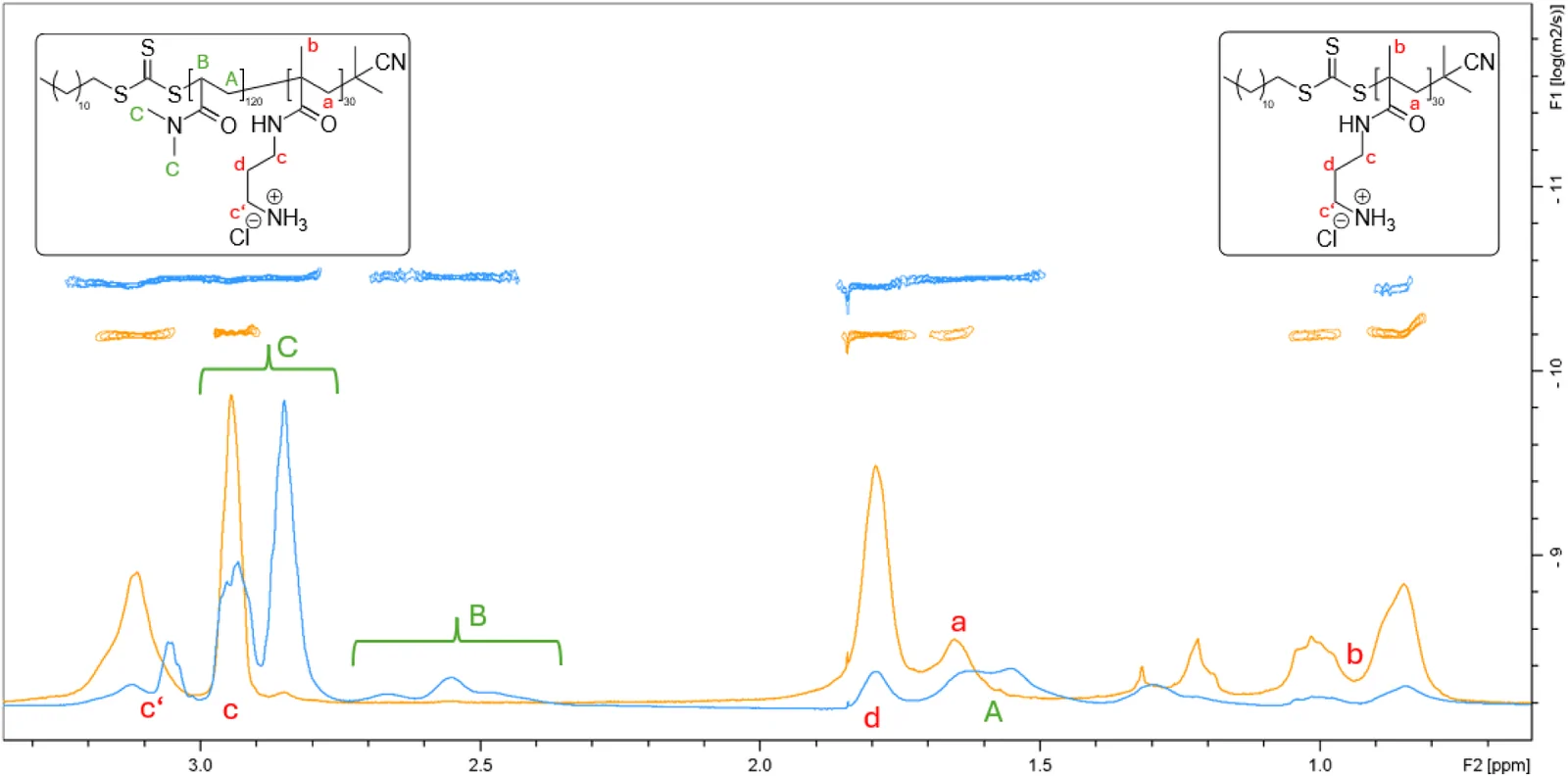
Diffusion-ordered NMR spectroscopy (DOSY) of PAPMA_30_-*b*-PDMAA_120_ (blue) and PAPMA_30_ (orange), with corresponding ^1^H NMR in D_2_O (concentration of polymer: 8.5 g/L; integrals used for DOSY spectrum; measured at 500 MHz).

### Redox-Switchable Aggregation of PAPMA_30_ **-** *b* **-**PDMAA_120_ in the Presence of Hexacyanoferrates

After the preparation of the corresponding block copolymers, we first investigated the solution properties of PAPMA-*b*-PDMAA alone in buffered brine in the presence of hexacyanoferrates. The variable complexation between these multivalent, redox-switchable counterions and the polyelectrolyte, is expected to lead to a controllable micellization process because of the concomitant solubility changes of the polycationic block. The pH of the acetate buffer was close to 4.7 to ensure almost full protonation of the PAPMA block for efficient electrostatic interaction with the hexacyanoferrates.^61,62^ Intriguingly, PAPMA-*b*-PDMAA shows aggregation when accompanied by hexacyanoferrate­(II) ions (ferrocyanide) but remains unimolecularly dissolved in the presence of hexacyanoferrate­(III) ions (ferricyanide) under the conditions used in this study (compare Scheme 1). As outlined before, previous studies already showed this effect by using PAPMA during electrochemical depositions on an electrode surface.^44^

**Scheme 1:**
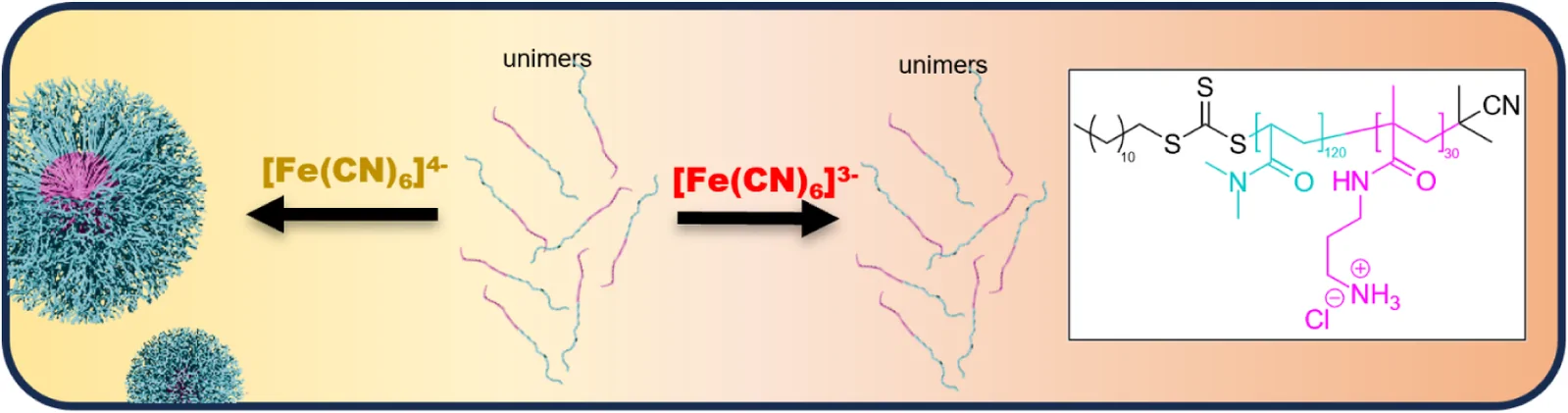
Aggregation Behavior of PAPMA_30_-*b*-PDMAA_120_ ^a^

a Addition of ferrocyanide to the polymer-containing solution leads to micellization because of PAPMA-hexacyanoferrate complexation, while hardly any complexation, and thus no micellization, occurs with ferricyanide in the chosen concentration regime.

First, this effect was more closely investigated by measuring Dynamic Light Scattering (DLS) at different concentrations of the respective iron species at a concentration of 1 g/L polymer, by mixing stock solutions of polymer and the corresponding hexacyanoferrate to yield the desired concentrations. Because the copolymer is charged, a salt concentration of 0.1 M KCl is used additionally to the buffer at all times for all solutions to provide a well-defined and hardly changing ionic strength. Additionally, the salt provides a necessary background electrolyte for the electrochemical experiments. Experiments at varying KCl concentrations (0–0.1 M) confirmed that the salt does not alter the fundamental switching mechanism up to 0.1 M KCl, though aggregate size and dispersity depend on ionic strength (details in SI, Section 2.5). Notably, 0.1 M KCl yields the most compact and monodisperse aggregates (*R*_h_ = 102 nm), supporting the choice for the optimal condition for reproducible characterization. Moreover, a limited addition of salt facilitates the establishment of the thermodynamically preferred conformation of the PAPMA-hexacyanoferrate complex (while 0.1 M KCl is still not enough to guarantee equilibrium morphologies for the PEO-*b*-qPDPAEMA complex; see below). The PAPMA_30_-*b*-PDMAA_120_ results are presented in Figure 4.

**Figure 4:**
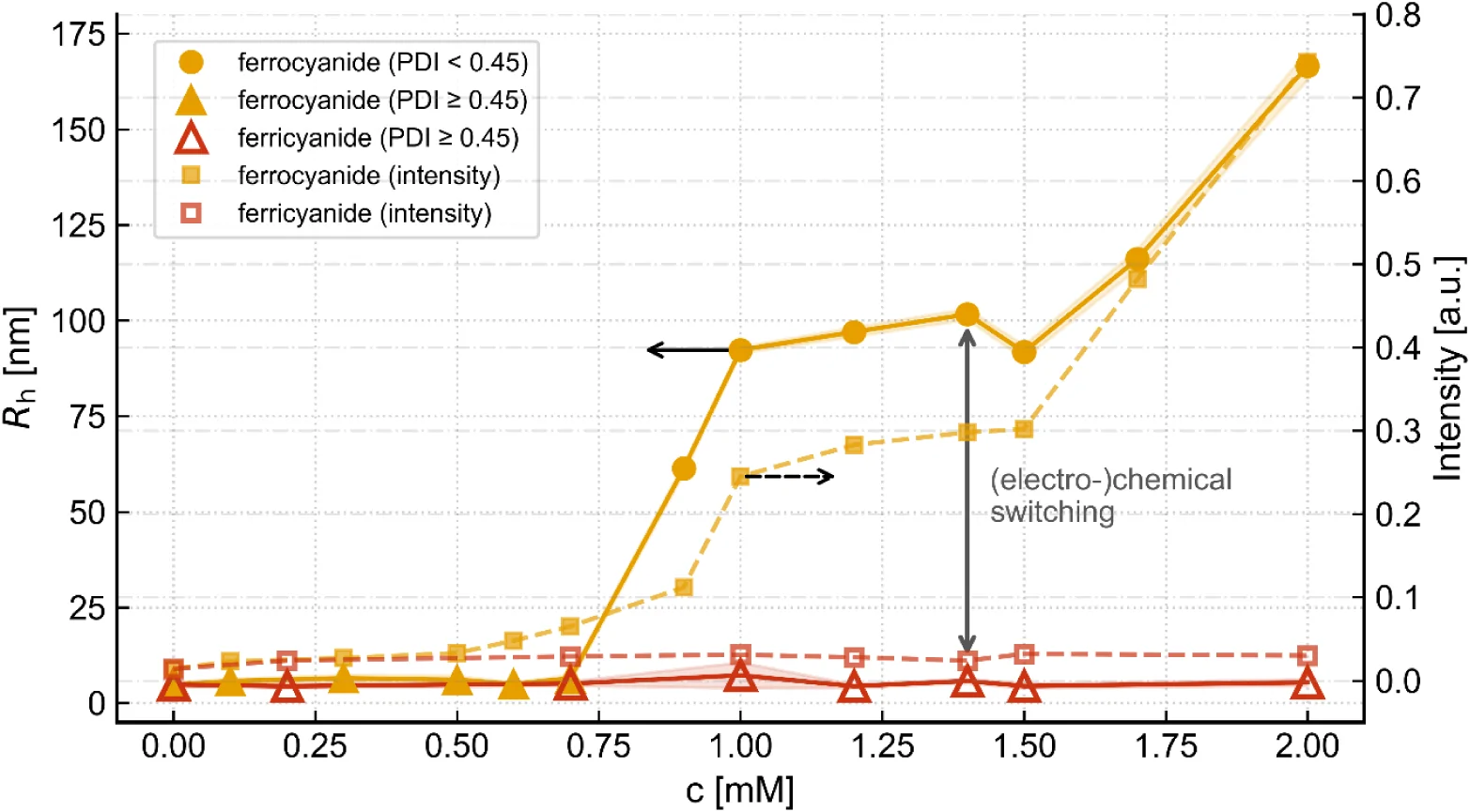
DLS measurements of PAPMA_30_-*b*-PDMAA_120_ (1 g/L) in 0.1 M KCl at pH 4.7 (buffered with sodium acetate) with different concentrations of either ferrocyanide (yellow, full symbols) or ferricyanide (red, open symbols). Dots (•) represent *R*_h_ values extracted from cumulant analysis, and triangles (▲) represent values from regularized fitting (loose aggregates not shown). Shaded regions represent error bounds calculated from diffusion coefficient analysis across multiple scattering angles. Squares (■ with dashed lines) represent the average scattering intensity at a 90° scattering angle for the respective measurement. Changing between the different aggregation states is possible by chemical oxidation/reduction of the iron species with H_2_O_2_ or ascorbic acid.

The polydispersity index (*PDI*) extracted from the cumulant analysis of the DLS data can provide an idea of the dispersity of the sample. A *PDI* threshold of 0.45 was established to categorize the dispersity characteristics of this polymer. The selection of the critical *PDI* value was specifically substantiated by a regularized fitting evaluation, which revealed a clear distinction in the multimodal behavior above and below this threshold. Samples with *PDI* ≥ 0.45 were classified as having high dispersity, consistently exhibiting multiple modes in the regularized fitting results, including loose aggregates alongside unimeric species (details Section 2.4 in SI^63^). Samples with *PDI* < 0.45 were classified as “low” dispersity samples, and the hydrodynamic radius (*R*_h_) values from cumulant fitting were utilized for these cases, as cumulant fitting is often the most robust technique for analyzing DLS data. However, additional small peaks can be observed in regularized fitting methods in some cases for these “low” dispersity samples (details Section 2.5 in SI^64^), but in comparison to the polydisperse samples, the mean scattering intensity is an order of magnitude higher (see Figure 4) and the decay time distributions are not as broad as expected, for example, loose aggregates. Generally, the intensity starts to slowly increase with 0.5 mM ferrocyanide, indicating the beginning of micelle formation. In conclusion, it can be assumed that some kind of micelle-like aggregation occurs in the case of the “low” dispersity samples.

As mentioned, in the case of the unimeric state exhibiting an *R*_h_ of 5.5 nm, additional loose aggregates with broad distributions are visible, which are not depicted in the figure for clarity. They are not partaking in the micellization, and their origin has been debated to this very day. Loose aggregates are often visible for polar polymers,^65^ though the majority of the polymer is not involved in the aggregation.^66^ While the hydrophobic dodecyl end group could contribute to the broad distribution of the loose aggregates, the hydrophilicity of the long PDMAA (and PAPMA) counterbalances this associative effect and leads to primarily unimolecularly dissolved polymer chains under the conditions used here. However, upon adding hexacyanoferrate, the size of the micellar aggregates reaches a plateau up until 1.5 mM ferrocyanide, before the micelles continue to grow at higher concentrations (Figure 4).

The aggregation was further confirmed by cryogenic Transmission Electron Microscopy (cryo-TEM). Here, the formation of small irregularly shaped aggregates with ferrocyanide can clearly be seen in Figure 5A. A representative sample size of 41 aggregates was used to determine an average radius of 10.3 nm (details Section 2.8 in SI^67^). Assuming that the contrast mainly arises from the micellar core, the observed radius is of the same order of magnitude as the contour length of a mildly disperse PAPMA block. Occasionally, larger features, being in line with the DLS data, can be found, as shown in Figure 5B. They are probably of vesicular nature (with wall thicknesses in the range of 6 nm, indicating considerable relaxation of chain stretching), while in rare cases, elongated structures are also seen (details Section 2.8 SI). In DLS, these large features dominate the scattering properties of the solution, resulting in the large *R*_*h*_ discussed above. Also, as mentioned, smaller aggregates are visible for intermediate concentrations with an increase in scattering intensity (compare Figure 4 and Section 2.5 in SI). Here, these abundant small aggregates visible in cryo-TEM might start forming, while the larger features start appearing at a higher concentration threshold, where they dominate the DLS measurement. The rather high *PDI* (<0.45) of the “low” dispersity measurements can be explained by the presence of these different types of aggregates. Noteworthy, the low PAPMA/PDMAA block ratio does not prevent the transition to vesicles. The presence of such higher-order micellar morphologies could be a consequence of the stratification of the insoluble complex of PAPMA and the rather bulky, hydrated ferrocyanides. However, the morphology of the aggregates is not as well-defined compared to other micellar systems (probably caused by a rather shallow minimum of the free energy landscape, in which the alkyl end group could play a balancing role). Notwithstanding, the aggregation in the presence of ferrocyanide is clearly demonstrated by DLS and cryo-TEM.

**Figure 5:**
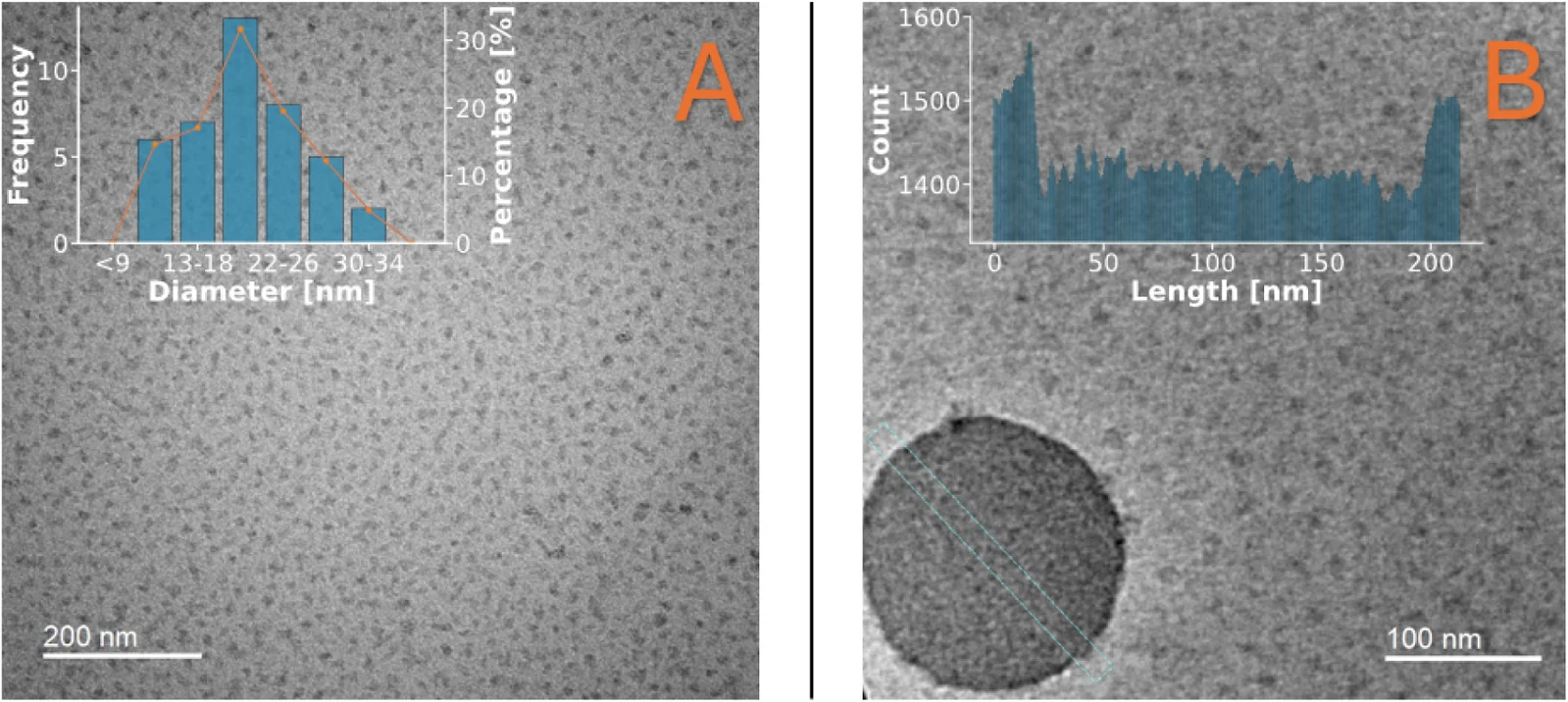
Cryogenic Transmission Electron Microscopy (cryo-TEM) images illustrating the PAPMA_30_-*b*-PDMAA_120_ aggregation behavior. (A) Polymer solution (1 g/L in 0.1 M KCl, pH 4.7, sodium acetate buffer) containing 1.4 mM ferrocyanide reveals the formation of small aggregates with irregular morphology. A size distribution from a representative set of samples is given as an inset (details Section 2.8 in SI). (B) An example of occasionally found larger structures with a line grayscale profile of a segment indicated in the micrograph.

The aggregation state can be reversibly switched between the unimolecularly dissolved state and the micelle-like state by chemical means via the addition of 2–3 droplets (approximately 100 μL) of either 0.1 M ascorbic acid or 3% hydrogen peroxide for reduction or oxidation, corresponding to a multifold molar excess over the hexacyanoferrate present. This procedure was tested for a solution containing 1.4 mM of the respective iron species, and the resulting *R*_h_ of the reduced/oxidized species aligns with the results found within the concentration series (see Figure 4; details Section 2.3 in SI). The successful oxidation or reduction is always indicated by a color change of the solution, because the ferricyanide solution has a distinct yellow color in comparison to the ferrocyanide solution.

Chemical switching poses a sudden change and thus a hard, abrupt trigger to induce the change in solution properties. Instead, one can use a soft trigger by changing the environment of the polymer gradually through electrochemical oxidation/reduction. Figure 6 demonstrates this concept through bulk electrolysis, where ferricyanide undergoes slow reduction to ferrocyanide, coinciding with aggregate formation. Using a comparative concentration of 1.4 mM ferricyanide, the system was monitored by periodically halting electrolysis to measure both the open-circuit potential (*OCP*) and hydrodynamic radius. The sufficient reduction/oxidation of the hexacyanoferrate species can be investigated using Faraday’s law and the Nernst equation, as shown in the section below comparing both polymeric systems. The whole process is reversible, and by electrochemical oxidation, the unimolecular state of the system can be restored. It is also possible to repeat this switching multiple times, as illustrated (Figure 6).

**Figure 6:**
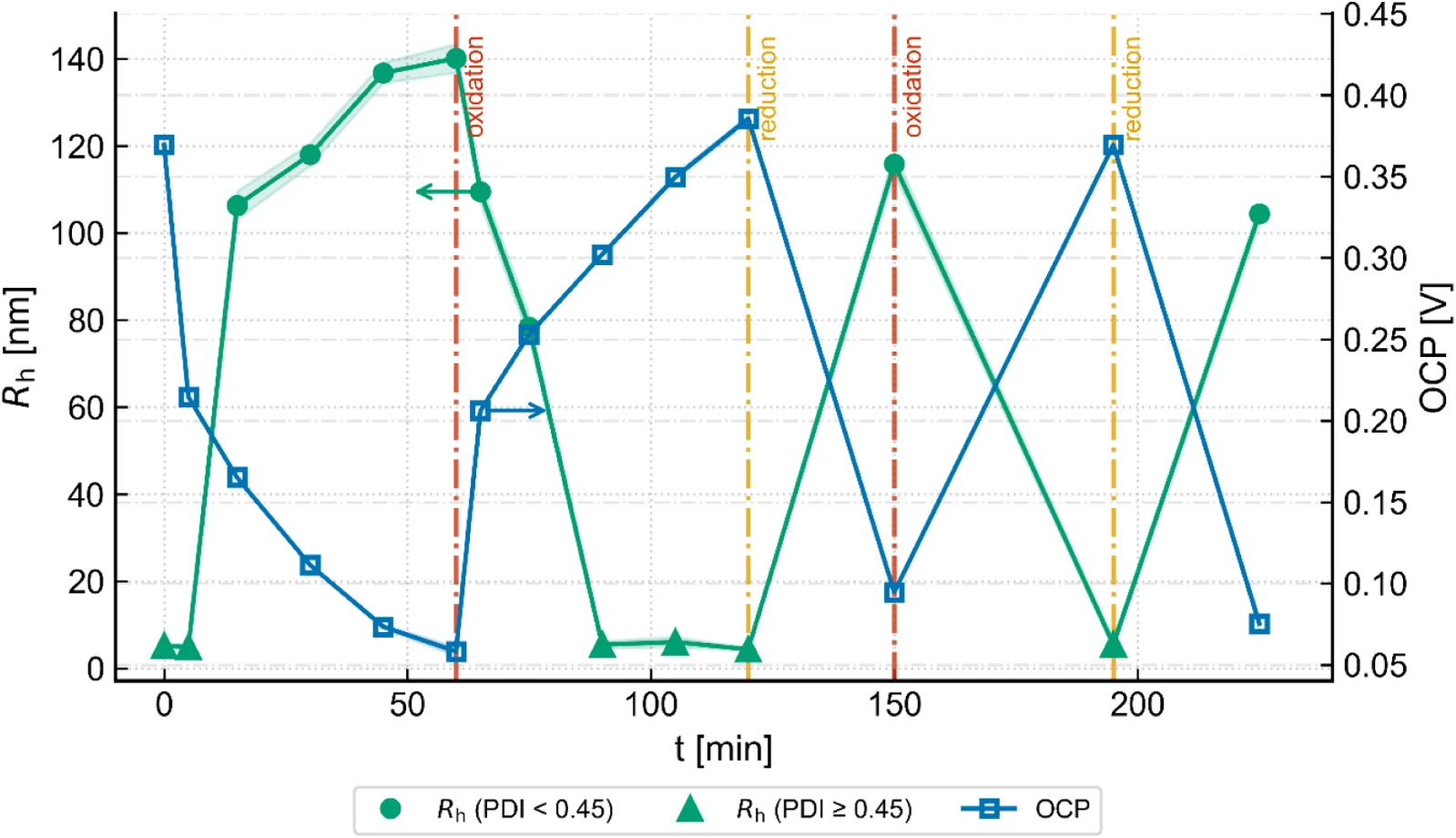
Electrochemical switching of PAPMA_30_-*b*-PDMAA_120_ (1 g/L) in 0.1 M KCl at pH 4.7 (buffered with sodium acetate) with an initial concentration of 1.4 mM ferricyanide. At each point, the bulk electrolysis was paused to measure *OCP* (open symbols) and *R*_h_. Dots (•) represent *R*_h_ values extracted from cumulant analysis, and triangles (▲) represent values from regularized fitting (loose aggregates not shown). The dashed-dotted lines indicate the time when the bulk electrolysis was switched between oxidation and reduction. Shaded regions represent error bounds calculated from diffusion coefficient analysis across multiple scattering angles.

### Redox-Switchable Aggregation of PEO_114_-*b*-qPDPAEMA_171_

To achieve orthogonal addressability with respect to different electrode potentials, the other polymer was chosen to be a block copolymer consisting of the popular nonionic hydrophilic polymer poly­(ethylene oxide) (PEO) and the polymer poly­(diisopropylaminoethyl methacrylate) (PDPAEMA) with the amino group being quaternized, which renders this polymeric block to be a strong cationic polyelectrolyte. The resulting block copolymer poly­(ethylene oxide)-*b*-poly­{[2-(methacryloyloxy)-ethyl]­diisopropylmethylammonium chloride} (PEO-*b*-qPDPAEMA) was already synthesized and characterized elsewhere.^23^ PEO-*b*-qPDPAEMA is similar to PAPMA-*b*-PDMAA in regard that it shows micellar aggregation with one hexacyanoferrate species and not with the other, but the sensitivity is antagonistic compared to PAPMA-*b*-PDMAA (i.e., it aggregates with ferricyanide but not with ferrocyanide; compare Scheme 2). In Figure 7, a concentration series for PEO-*b*-qPDPAEMA is illustrated. Note that this polymer is not sensitive to the pH as a strong polyelectrolyte, but salt content and pH were kept constant for comparative purposes. Also, a lower concentration of polymer was chosen to account for the higher polycationic block length. The concentration of charge for the chosen polymer concentration of 0.2 g/L is roughly 0.7 mM. Compared with the previous PAPMA-*b*-PDMAA samples, the lower polymer concentration is justified by a common switchability window for both systems (see below). The unimolecular state of this polymer has an average *R*_h_ of 8.7 nm, which is larger than that of the other diblock copolymer. However, the increased size was anticipated because the polymer chain is longer. Loose aggregates also appear in some rarer cases. As observed for relatively low ferricyanide concentrations, aggregation occurs, resulting in somewhat larger, probably loosely assembled micelles with swollen cores at low ferricyanide concentrations (55 nm). However, the size of these micelles levels off at about 35 nm for higher metalate concentrations.

**Scheme 2:**
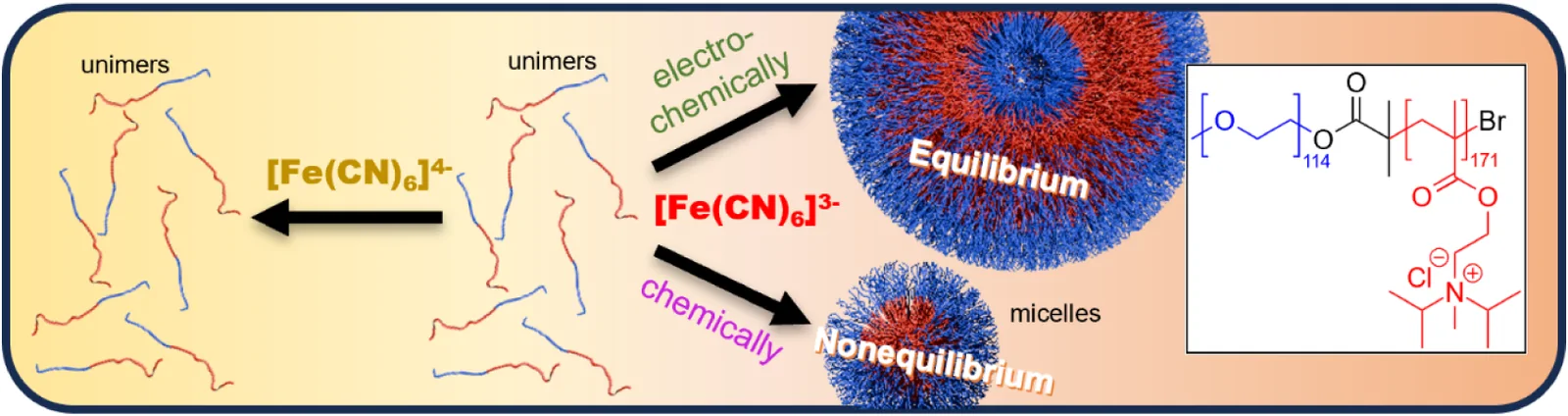
Aggregation Behavior of PEO_114_-*b*-qPDPAEMA_171_ ^a^

a The polymer’s interaction in solution with hexacyanoferrate leads to negligible complexation of qPDPAEMA with ferrocyanide in the chosen concentration regime, but qPDPAEMA complexation with ferricyanide leads to different morphologies depending on the pathway. Addition of ferricyanide acts as a hard trigger, leading most likely to kinetically trapped micelles, while electrochemical treatment, as a soft trigger, results in a vesicular morphology being more in line with the thermodynamically favored one because of the increased block ratio (qPDPAEMA/PEO).

**Figure 7:**
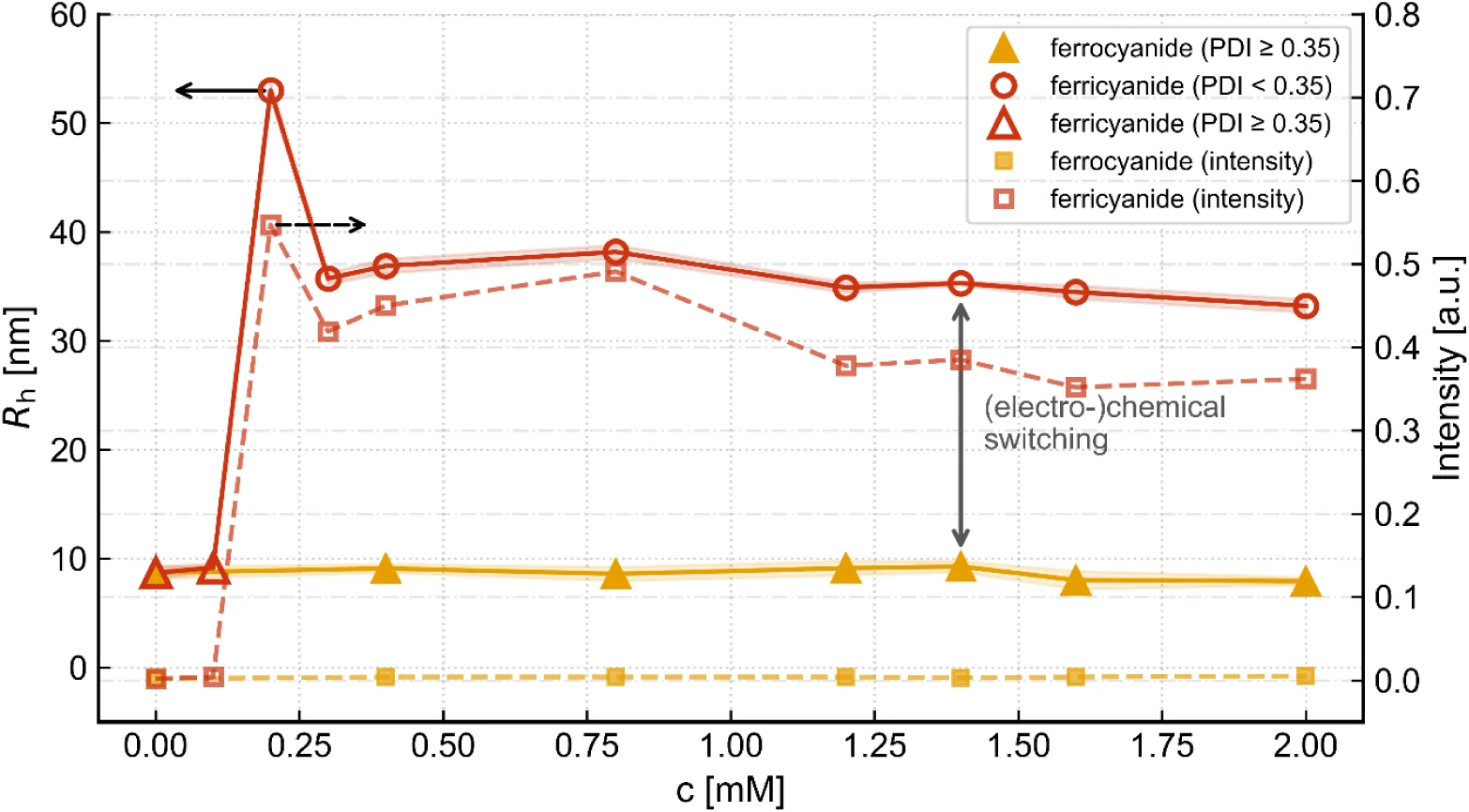
DLS measurements of PEO_114_-*b*-qPDPAEMA_171_ (0.2 g/L) in 0.1 M KCl at pH 4.7 (buffered with sodium acetate) with different concentrations of either ferrocyanide (yellow, full symbols) or ferricyanide (red, open symbols). Dots (•) represent *R*_h_ values extracted from cumulant analysis, and triangles (▲) represent values from regularized fitting (loose aggregates not shown). Shaded regions represent error bounds calculated from diffusion coefficient analysis across multiple scattering angles. Squares (■ with dashed line) represent the average scattering intensity at a 90° scattering angle for the respective measurement. Changing between the different aggregation states is possible by chemical oxidation/reduction of the iron species with H_2_O_2_ or ascorbic acid.

Switching between these states is, again, possible by chemically induced stimulation with the same procedure as for PAPMA-*b*-PDMAA (details Section 2.3 in SI).

Besides chemical switching, an electrochemically induced change in the micellization state can also be observed, as displayed in Figure 8. Starting with pure ferricyanide in the micellar state, electrochemical reduction leads to the growth of these aggregates to radii in the range of 400 nm. The observation of larger radii at low ferricyanide concentrations aligns with the larger entities observed in Figure 7, which are mainly caused by loosely electrostatically cross-linked polyelectrolyte aggregates with high water content. It must be kept in mind that the completely reduced system is sensitive to oxygen and light,^68^ which is treated accordingly (details in Section 2.9 in SI).

**Figure 8:**
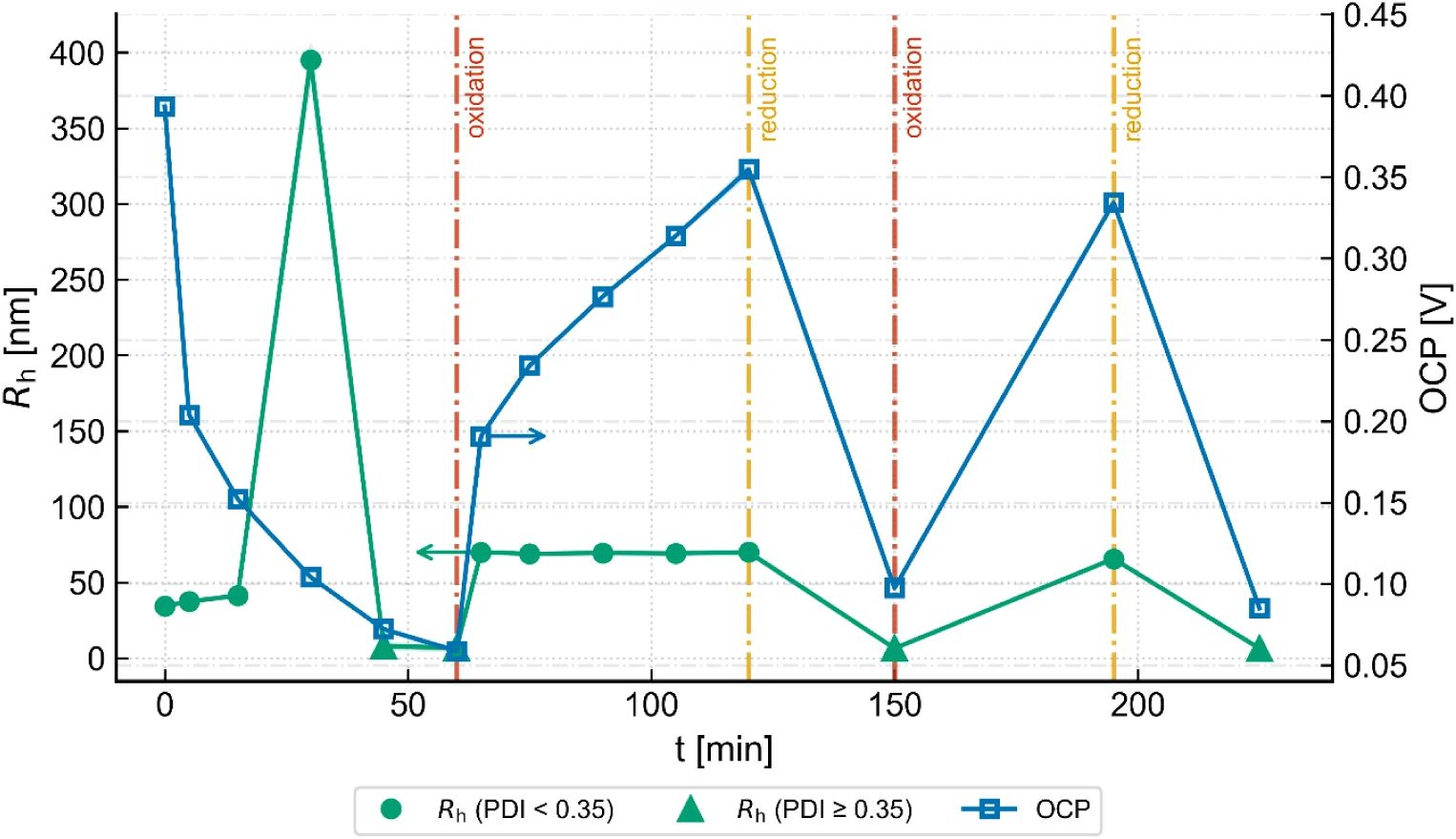
Electrochemical switching of PEO_114_-*b*-qPDPAEMA_171_ (0.2 g/L) in 0.1 M KCl at pH 4.7 (buffered with sodium acetate) with a starting concentration of 1.4 mM ferricyanide. At each point, the bulk electrolysis was paused to measure *OCP* (open symbols) and *R*_h_. Dots (•) represent *R*_h_ values extracted from cumulant analysis, and triangles (▲) represent values from regularized fitting (loose aggregates not shown), and the dashed-dotted lines indicate the times when the bulk electrolysis was switched between oxidation and reduction. Shaded regions represent error bounds calculated from diffusion coefficient analysis across multiple scattering angles.

Oxidation of this unimolecular state by electrochemical means does not lead to the initial micellar size but rather to micelles with an *R*_h_ of 70 nm. Thus, it is possible to gain differently sized aggregates or even different micellar morphologies depending on the oxidation trigger. The hard and fast trigger of chemically induced oxidation/reduction leads probably to nonequilibrium micelles (kinetically trapped structures that do not represent the thermodynamic free energy minimum) close to a crew-cut morphology (compact spherical micelles in which the size of the solvophobic core chains greatly exceeds that of the solvophilic corona, resembling a short-cropped haircut), which were also obtained upon direct mixing of the polymer and ferricyanide.^69^ In view of the rather long solvophobic block and the qualitative picture provided by the packing parameter,^70^ a different morphology than the compact spherical crew-cut/core–corona structure could be expected as the thermodynamically preferred one. Hence, the slow and soft trigger of electrochemically induced oxidation/reduction leads likely to a favored and thermodynamically more stable state (details Section 2.9 in the SI). By oxidation and reduction, it is possible to cycle between the two states multiple times.

The different shapes of these two kinds of micellar structures were further investigated by cryo-TEM, which is especially sensitive to the solvophobic component with high electron density. In contrast, the technique is rather blind to the solvophilic corona domain with its near water-like contrast. The results from cryo-TEM are presented in Figures 9 and 10. In Figure 9, the images of the polymer solution (0.2 g/L PEO_114_-*b*-qPDPAEMA_171_ in 0.1 M KCl at pH 4.7) with the addition of 1.4 mM ferricyanide are shown, in which micellar structures become visible. A size analysis of 77 micelles reveals an average radius of 27.9 nm. In addition to these prominent micelles, smaller aggregates are evident, exclusively confined to the carbon support, which could be a result of the interaction with the charged TEM grid (details Section 2.8 in SI). These smaller species may represent additional iron-complexed aggregates. However, their scattering contribution, if present in the bulk at all, is most likely obscured by the larger micelles in DLS measurements. Additionally, dendritic/fractal structures are visible throughout the grid (compare Figure 9A,B and Figure 10A). These artifacts originate likely from interactions between free ferricyanide and plasma-treated copper TEM grids under the formation of Prussian blue analogues,^71^ since they are also seen when investigating polymer-free ferricyanide solution by cryo-TEM (details Section 2.8 in SI). Magnification of single micelles reveals a not-totally-uniform contrast of these structures, indicating that fluctuations in the local ferricyanide density might be present under the conditions used for cryo-TEM.

**Figure 9:**
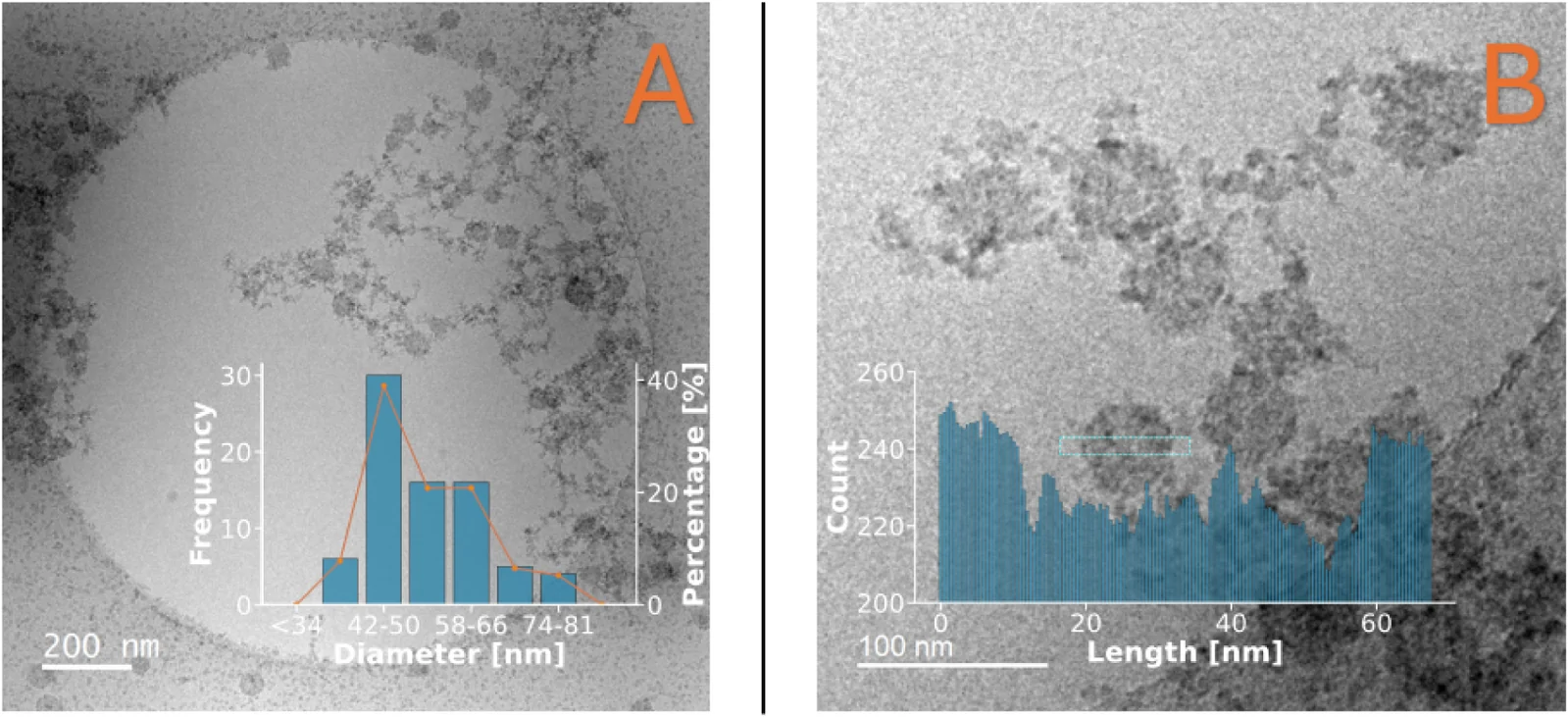
Cryo-TEM images of PEO_114_-*b*-qPDPAEMA_171_ nonequilibrium micelles. (A) Polymer solution (0.2 g/L polymer in 0.1 M KCl, pH 4.7, sodium acetate buffer) with 1.4 mM ferricyanide showing micelles and small aggregates on the carbon support grid. Micellar size on a representative sample of structures is determined to provide an indication of typical dimensions (details Section 2.8 in SI). In (B), magnifications of micelles with one exemplary line profile. Dendritic patterns seem to stem from ferricyanide (details Section 2.8 in SI).

**Figure 10:**
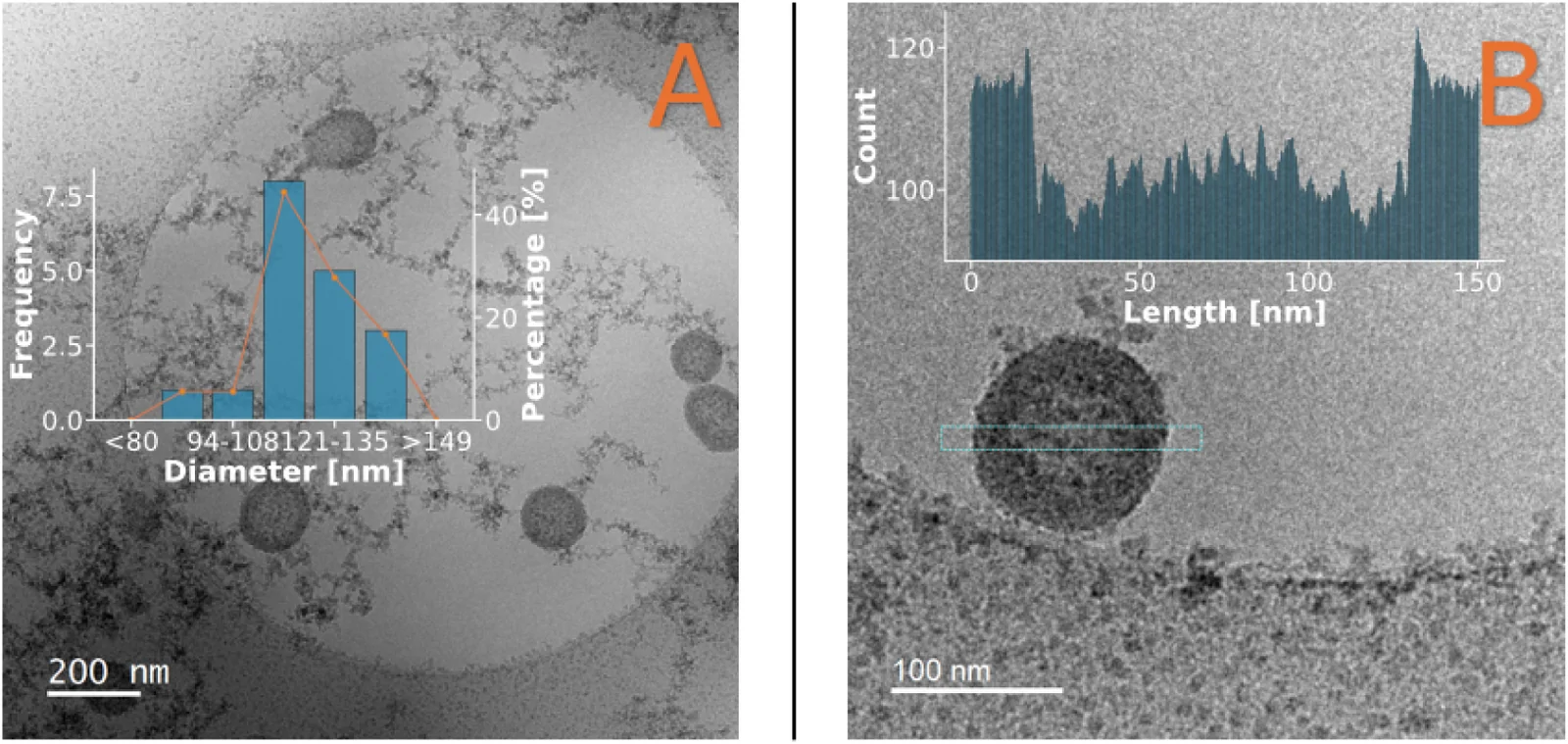
Cryo-TEM images of PEO_114_-*b*-qPDPAEMA_171_ electrochemically assembled equilibrium structures. (A) Polymer solution (0.2 g/L polymer in 0.1 M KCl, pH 4.7, sodium acetate buffer) with 1.4 mM ferricyanide after electrochemical assembly for size determination on a representative sample of structures to provide an indication of typical dimensions. Dendritic patterns seem to stem from ferricyanide (details Section 2.8 in SI). (B) More detailed image of a single vesicle with a grayscale line profile.

Figure 10 shows the electrochemically treated solution after plunge-freezing, revealing vesicular structures. Analysis of 18 vesicles yielded an average radius of 60.3 nm, which is again in agreement with the DLS results. A single micelle is depicted in a magnified view in Figure 10B, where the indents in the grayscale line profile toward the edges speak in favor of a vesicular morphology. Again, dendritic structures were visible. While the presence of these dendritic structures aggravates the analysis, the cryo-TEM micrographs unequivocally demonstrate different self-assembly states of the two polymers.

In light of the possible artifacts observed in cryo-TEM for ferricyanide-containing samples, small-angle X-ray scattering (SAXS) was employed to obtain an averaged picture without interaction with the copper grid (Figure 11). Cryo-TEM images reveal that both nonequilibrium and equilibrium micelles exhibit spherical morphologies, suggesting the use of a core–shell-sphere form factor fit for the analysis of the SAXS data. However, the scattering length density (*sld*) profiles of these micelles differ significantly. For nonequilibrium micelles, the *sld* of the core dominates over that of the shell (details Section 2.10 in SI^72,73^ ), making a simpler spherical form factor model sufficient. Thus, the initial nonequilibrium micelles are fitted with a sphere form factor (Figure 11) with a radius of approximately 25 nm, which is in perfect agreement with the data from cryo-TEM and previously published data for this polymer with larger polymer and ferricyanide concentrations.^69^ The PEO corona would contribute a further 10 nm to the *R*_h_ in DLS. In contrast, the *sld* situation is reversed for the equilibrium structures after an electrochemical redox cycle, with the shell contributing more significantly (details Section 2.10 in the SI). Therefore, a hollow-sphere/vesicular model is more appropriate, giving an approximate total radius of 40 nm. This total radius is constituted of an inner radius of 17 nm and a shell thickness of 23 nm, coinciding with the observations from cryo-TEM. Assuming a stronger stretching of the PEO corona in the vesicular morphology, *R*_h_ of 70 nm can be explained by these SAXS results. Further, any differences in the vesicular sizes between the different methods can be explained by the fact that vesicles can possess a broad range of sizes: the actual outcome depends on the history of the sample. Hence, different times between preparation and measurement might already influence the average size of the vesicles.

**Figure 11:**
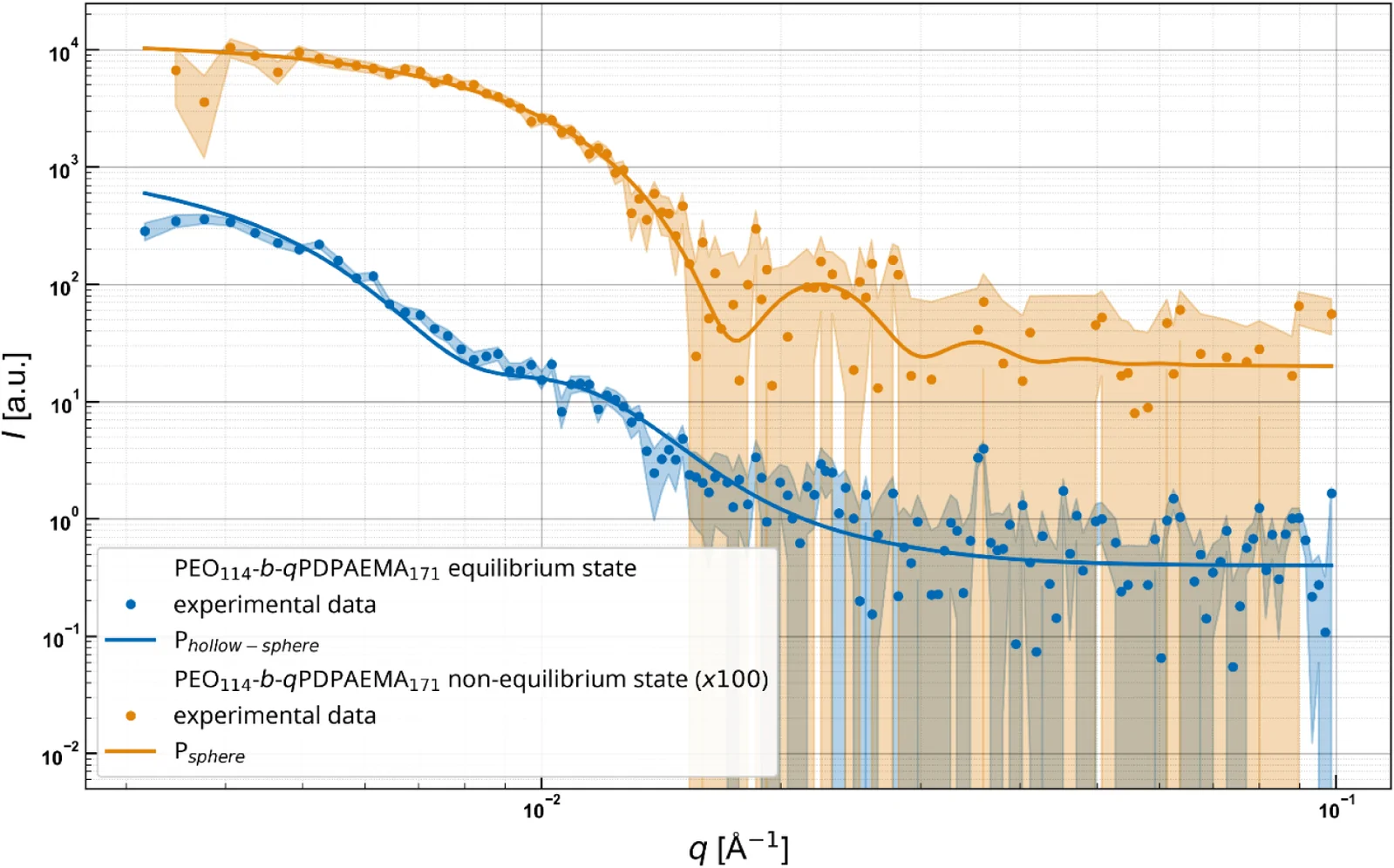
Small-angle X-ray scattering profiles of PEO_114_-*b*-qPDPAEMA_171_ (0.2 g/L) in 0.1 M KCl at pH 4.7 with 1.4 mM ferricyanide. Orange: in its initial nonequilibrium state, fitted with a hard sphere form factor model, resulting in a radius of approximately 25 nm. Blue: in its equilibrium state after electrochemical treatment, fitted with a hollow sphere form factor model, resulting in a total radius of approximately 40 nm.

In conclusion, both cryo-TEM and SAXS provide a congruent morphological picture, as both techniques mainly image the solvophobic domain. Strikingly, the electrochemical redox cycle leads to a different morphology of the polymer in comparison to the initial state, where a transition from kinetically trapped, nonequilibrium crew-cut micelles to vesicles close to equilibrium could explain the observed changes.

### Comparison of the Two Different Polymers

As outlined above, both diblock copolymers can exhibit amphiphilic behavior. The PAPMA block of PAPMA-*b*-PDMAA becomes amphiphilic when a sufficient amount of ferrocyanide is present, while the qPDPAEMA block of PEO-*b*-qPDPAEMA becomes amphiphilic in the presence of ferricyanide. Otherwise, each polymer appears bis-hydrophilic. As one of the blocks becomes hydrophobic, micellization occurs due to its resulting amphiphilic character. This complementary selectivity may seem counterintuitive, given that both interactions are electrostatically driven and ferrocyanide carries a higher overall charge than ferricyanide. The electron configuration and the presence of an unpaired electron in ferricyanide create a situation where its electron cloud is more easily distorted by an external electric field, rendering it more polarizable. Compared to, e.g., a quaternized ammonium group, where the positive charge experiences greater shielding from alkyl substituents resulting in lower charge density and higher polarizability (as shown in detail by Neubert et al. to bind ferricyanide selectively via pseudocontact ion pairing^24^), the protonated amine with its concentrated positive charge is less polarizable. Thus, the less polarizable ferrocyanide preferably interacts with the less polarizable protonated, primary ammonium group. Overall, these findings are consistent with the Pearson hard/soft acid–base principle. It can be switched between aggregated states and unimeric states by chemical and electrochemical means, whereas in the case of PEO-*b*-qPDPAEMA two different types of micellar morphologies can be achieved depending on the trigger. In sum, the interaction of the hexacyanoferrates with the polycationic blocks can be used to achieve an electrochemically controlled micellization/aggregation process.

Generally, micelles of the latter polymer show a lower *PDI* in cumulant analysis (with values sometimes even below 0.1) compared to PAPMA-*b*-PDMAA, indicating that the structures of the PAPMA-*b*-PDMAA aggregates are less defined than those of the PEO-*b*-qPDPAEMA aggregates. The robustness of the PEO-*b*-qPDPAEMA micellizations is underlined by the higher mean scattering intensity measured in DLS for PEO-*b*-qPDPAEMA despite a 5-fold lower concentration compared to PAPMA-*b*-PDMAA (compare Figures 4 and 7). In this context, it is worth pointing out that PEO-*b*-qPDPAEMA can be rather well characterized by SAXS using the same dilute concentration as in DLS (0.2 g/L), while no structures can be resolved for PAPMA-*b*-PDMAA. This was also observed for a different polymer system,^66^ where this behavior was explained by a high water uptake of the system, rendering the aggregates invisible due to contrast/sensitivity issues. Unlike PEO-*b*-qPDPAEMA, SAXS analysis of the aggregates/micelles made of PAPMA-*b*-PDMAA is troublesome, even at higher concentrations when keeping the hexacyanoferrate-to-polymer ratio. In turn, nearly identical PEO-*b*-qPDPAEMA micelles were detected at different polymer concentrations varying by about 2 orders of magnitude (details Section 2.11 in SI^74,75^ ). Nonetheless, despite the considerably shorter stimuli-responsive block (the block ratio situation was originally intended to achieve different micellar morphologies for both polymers, which could help their differentiation during analysis of the solution behavior with small, star-like micelles for PAPMA-*b*-PDMAA vs larger vesicles/crew-cut micelles for PEO-*b*-qPDPAEMA), PAPMA-*b*-PDMAA aggregates show larger *R*_h_ values than PEO-*b*-qPDPAEMA micelles, suggesting a broad size distribution with contributions from multiple morphologies, consistent with the elevated PDI and cryo-TEM observations.

A parameter of interest for the description of the different systems is the initial charge ratio (*icr*). It is the ratio of the molar amount of charges exhibited by the added hexacyanoferrate species to the molar amount of charges of the polymer present. Taking the molar ratio of the PAPMA block of 0.2 into account, the concentration of the cationic polymeric charge is about 1.7 mM for a polymer concentration of 1 g/L. Hence, the *icr* at which the aggregation starts to occur is approximately 2.1 for the conditions used here (see 0.9 mM ferrocyanide solution in Figure 4). The required excess of ferrocyanide indicates an equilibrium with a rather low equilibrium constant, dealing in most cases with nonstoichiometric complexes (i.e., there is always a discrepancy between the initial and nominal charge ratio, where the latter refers to the actual polymer complex). For the qPDPAEMA block, on the other hand, the cationic polymeric charge is roughly 0.7 mM for the chosen concentration of 0.2 g/L, where successful micellization already occurs at an *icr* of 1. Thus, comparing the initial charge ratio of both systems, it is quite clear that a lower *icr* is sufficient for PEO-*b*-qPDPAEMA to form micelles, underlining the fact that the interaction between ferricyanide and qPDPAEMA is stronger than the one between ferrocyanide and PAPMA under the conditions used here. Additionally, the range of *icr* does not seem to play such a big role for the former polymer. While increasing the ferricyanide concentration and thus the *icr*, there is no noticeable change induced in the hydrodynamic radius for PEO-*b*-qPDPAEMA over a large concentration range (also true for radii determined by SAXS, Section 2.11 in SI), whereas different behavior with increasing *icr* is observed for PAPMA-*b*-PDMAA (compare Figures 4 and 7).

If each individual polymer is investigated at different ratios of ferro- and ferricyanide (total iron species concentration of 1.4 mM), as depicted in Figure 12 (top), the result closely matches the anticipated outcome. Even a rather low percentage of ferricyanide leads to micelles of PEO-*b*-qPDPAEMA averaging at an *R*_h_ of approximately 36 nm. In contrast, even minor amounts of ferricyanide in the ferri-/ferrocyanide mixtures lead to a decrease in size and the disappearance of the PAPMA-*b*-PDMAA aggregate structures. At a ferricyanide ratio of 25%, the ferricyanide concentration is 0.35 mM, which corresponds to a ferrocyanide concentration of 1.05 mM. At this concentration, usual micelles are expected to form for PEO-*b*-qPDPAEMA, as experimentally demonstrated in Figure 7. Similarly, PAPMA-*b*-PDMAA aggregates are expected at the offered amount of ferrocyanide, which are, however, absent under these conditions (*PDI* = 0.57). It is most likely that ferricyanide interferes with the PAPMA/ferrocyanide interaction: the higher ionic strength of the solution might reduce the range of electrostatic interactions between ferrocyanide and the cationic PAPMA block. Hence, the ferricyanide ratio needs to be smaller to also induce PAPMA aggregation.

**Figure 12:**
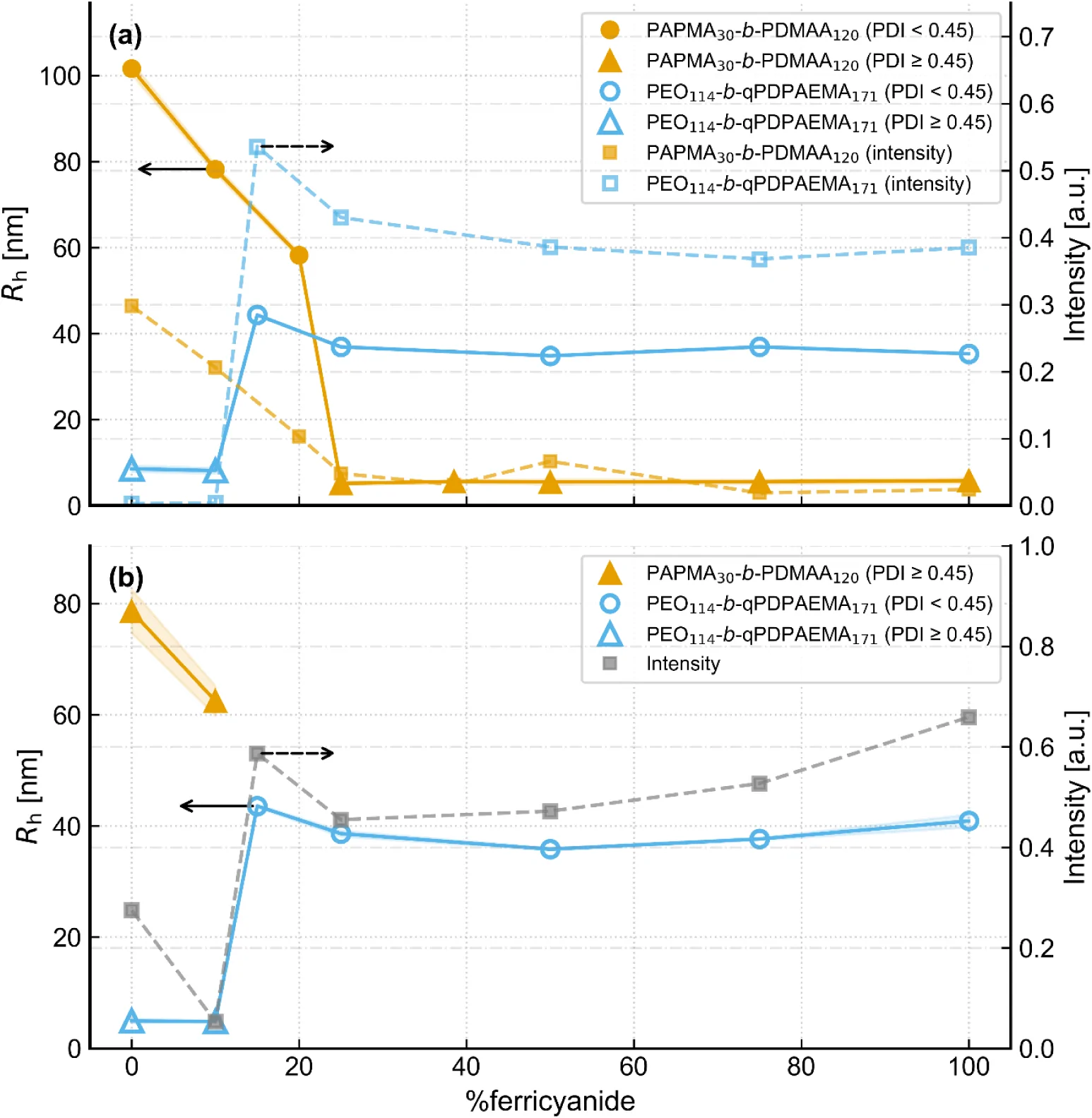
(a) Solution behavior of PEO_114_-*b*-qPDPAEMA_171_ (0.2 g/L; blue, open symbols) or PAPMA_30_-*b*-PDMAA_120_ (1 g/L; yellow, closed symbols) in 0.1 M KCl at pH 4.7 with different ratios of ferri- to ferrocyanide at a total concentration of iron species of 1.4 mM. (b) System containing PEO_114_-*b*-qPDPAEMA_171_ (0.2 g/L) and PAPMA_30_-*b*-PDMAA_120_ (1 g/L) simultaneously in 0.1 M KCl at pH 4.7 with different ratios of ferri- to ferrocyanide at a total concentration of iron species of 1.4 mM. In both cases, dots (•) represent *R*_h_ values extracted from cumulant analysis, and triangles (▲) represent values from regularized fitting (loose aggregates not shown). Shaded regions represent error bounds calculated from diffusion coefficient analysis across multiple scattering angles. Squares (■ with dashed lines) represent the average scattering intensity at a 90° scattering angle for the respective measurement. Changing between the different aggregation states at the extremes for (a) is possible by chemical oxidation/reduction of the iron species with H_2_O_2_ or ascorbic acid.

As shown in Figure 12 (top), there is only a narrow hexacyanoferrate ratio range, where polymers of both systems could coexist within micelles (above 10% and below 25% ferricyanide). Hence, we investigated the solution properties after mixing both aggregational states in that hexacyanoferrate concentration range, exemplarily at 21% ferricyanide (details Section 2.6 in the SI). Mixing the micelles and aggregates of both systems leads to a nearly monomodal distribution (Figure 13). Thorough, regularized DLS multiangle analyses are able to differentiate hydrodynamic sizes differing by a factor of 3-4,^76,77^ as long as the scattering contribution is comparable (see Figures 4 and 7) and the polydispersity is narrow enough for both populations, which is the case here. Hence, our monomodal size distribution seems to be more in line with a combinatorial type of disperse aggregates built up by both polymers. Instead of two distinct micellar populations, mixed micellar aggregates are likely to occur in our system, where the resulting micelles are larger than the PEO-*b*-qPDPAEMA micelles and smaller than the PAPMA-*b*-PDMAA micelles (see a more detailed discussion in the SI).

**Figure 13:**
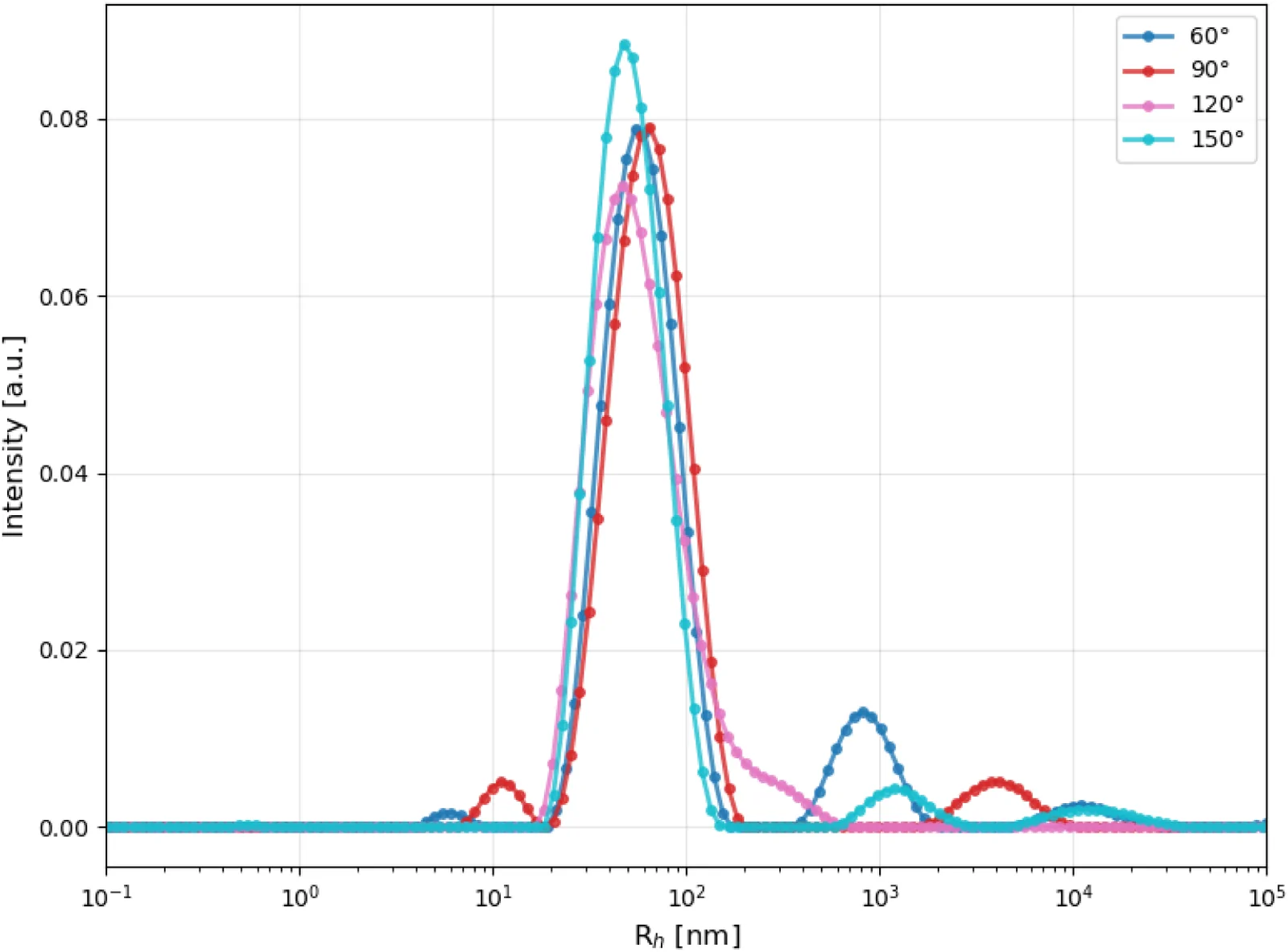
Size distribution at different scattering angles for the system of mixing micellar PEO_114_-*b*-qPDPAEMA_171_ and PAPMA_30_-*b*-PDMAA_120_ solutions in 0.1 M KCl at pH 4.7, where one peak clearly dominates. Mixing the micellar solutions leads to concentrations of 0.3 mM ferricyanide + 1.1 mM ferrocyanide (21% ferricyanide) with 0.2 g/L PEO-*b*-qPDPAEMA + 1 g/L PAPMA-*b*-PDMAA.

### Combined System of Both Polymers

The complementary ion selectivity of the two block copolymers raises the question of whether electrochemistry alone, by modulating the ferri/ferrocyanide ratio and the solution pH, can independently control the aggregation state of each polymer within a shared solution. In principle, this electrochemical control would allow full control over four distinct states: only PAPMA-*b*-PDMAA aggregated, only PEO-*b*-qPDPAEMA aggregated, both aggregated simultaneously, or neither aggregated (Scheme 3). Achieving this level of addressability with a single external stimulus in a two-component system is the central motivation for studying the combined polymer systems. Toward testing whether full control is possible or not, the system containing both polymers, with a concentration of 1 g/L PAPMA-*b*-PDMAA and 0.2 g/L PEO-*b*-qPDPAEMA, was examined at a constant total concentration of hexacyanoferrates of 1.4 mM. This mass ratio was established to maintain charge stoichiometry between the two polymers, with the lower concentration of PEO-*b*-qPDPAEMA offsetting its longer charged block, while also trying to keep the ionic strength of the system at a reasonable value. First, the polymer solution was tested via DLS with varying ratios of ferri- to ferrocyanide, where the observations are presented in Figure 12 (bottom). For no or low amounts of ferricyanide, regularized fitting reveals the presence of unimers and aggregates, probably of PAPMA-*b*-PDMAA, which then start to disappear at higher ferricyanide concentrations. The detection of the PEO-*b*-qPDPAEMA unimers is possible because of the lower scattering contribution of the PAPMA-*b*-PDMAA, which could be a consequence of the overall lower aggregation number of this polymer. In contrast, the PAPMA-*b*-PDMAA unimers at high ferricyanide contents are not visible in DLS because of the dominant scattering of the dense crew-cut PEO-*b*-qPDPAEMA micelles (average *R*_h_ = 39.3 nm), which is close to the value for the pure system. The absence of the unimeric state of PAPMA-*b*-PDMAA at high ferricyanide concentrations again suggests that the structures are more compact with sufficiently high aggregation numbers in the case of PEO-*b*-qPDPAEMA, and the scattering of the latter molecule overshadows that of the PAPMA-*b*-PDMAA unimers. Again, it is possible to switch between both states chemically (details Section 2.3 in SI).

**Scheme 3:**
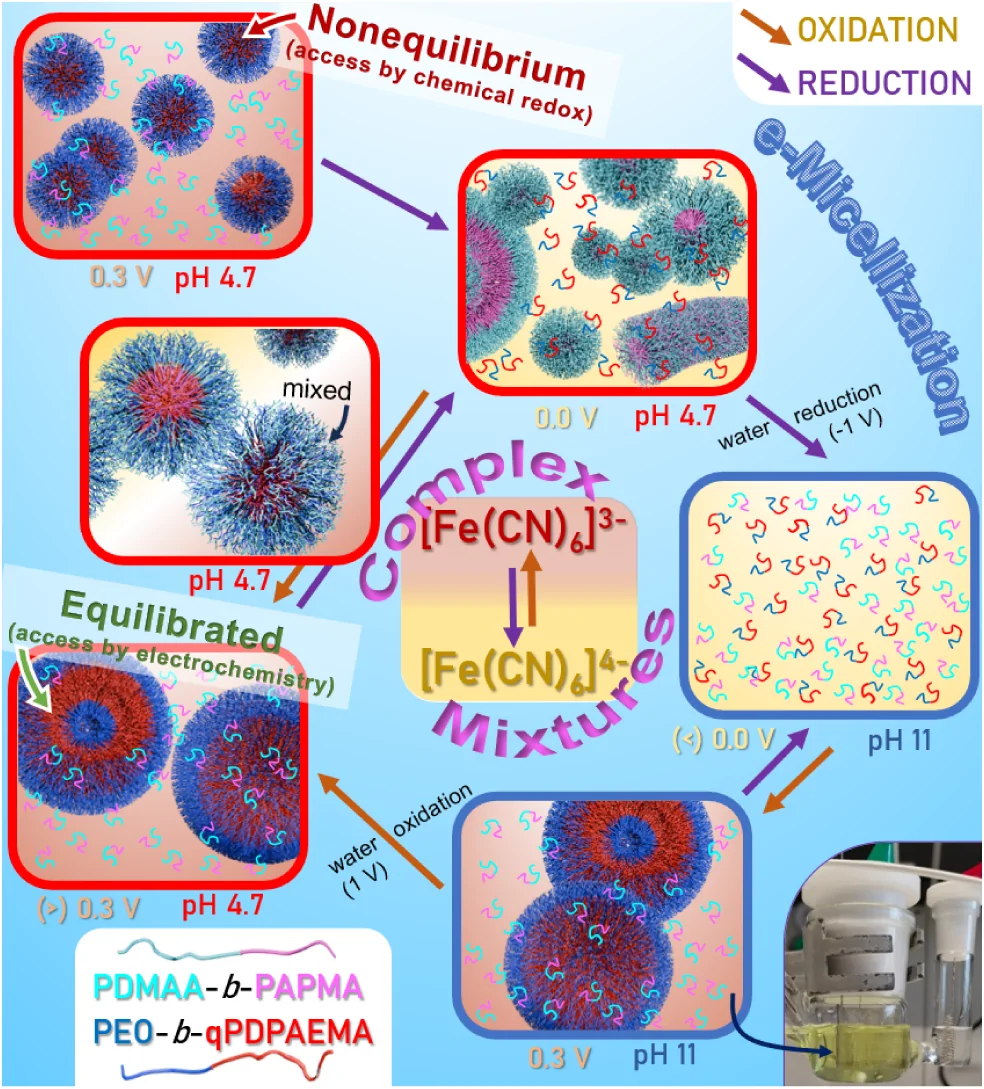
Possible Aggregation Behavior of Mixtures of PEO_114_-*b*-qPDPAEMA_171_ and PAPMA_30_-*b*-PDMAA_120_ ^a^

a Full control is at hand, where either just one type of polymer is aggregated (mutually switchable), both types of polymers are involved in micellization simultaneously, or none of the polymers are present in micellar form.

As before, the electrochemical pathway is also explored, which is visualized in Figure 14. At the beginning, one can see the (nonequilibrium) PEO-*b*-qPDPAEMA micelles with an average *R*_h_ of 39.8 nm, similar to that observed earlier in the experiment with different hexacyanoferrate ratios. Sufficient reduction of the system leads to the appearance of the PAPMA-*b*-PDMAA aggregates at a radius of ∼100 nm. For the points represented with a triangle, peaks of unimeric polymer can also be found through regularized fitting. Oxidation of this solution leads to a transformation of the nonequilibrium micelles into the equilibrated micelles of PEO-*b*-qPDPAEMA as seen in the experiment shown in Figure 8. The average *R*_h_ with 65 nm, is now slightly smaller than in the previous experiment (7% smaller). As before, the higher ionic strength of the system, due to the presence of the other cationic polymer, might decrease the range of electrostatic interactions, leading to fewer polymers being incorporated into one complex. Alternatively, the micelles may be more compact because of the higher charge involved in the system. Irrespective of these subtle differences, alternating electrochemical reduction and oxidation now enable a switch between the aggregates of both polymers. At the transitions, mixed micelles are expected to occur, as indicated in Figures 12 and 13, though the adjustment of the *OCP* to the narrow hexacyanoferrate ratio regime, where both polymers aggregate, is challenging compared to simply going to the extremes.

**Figure 14:**
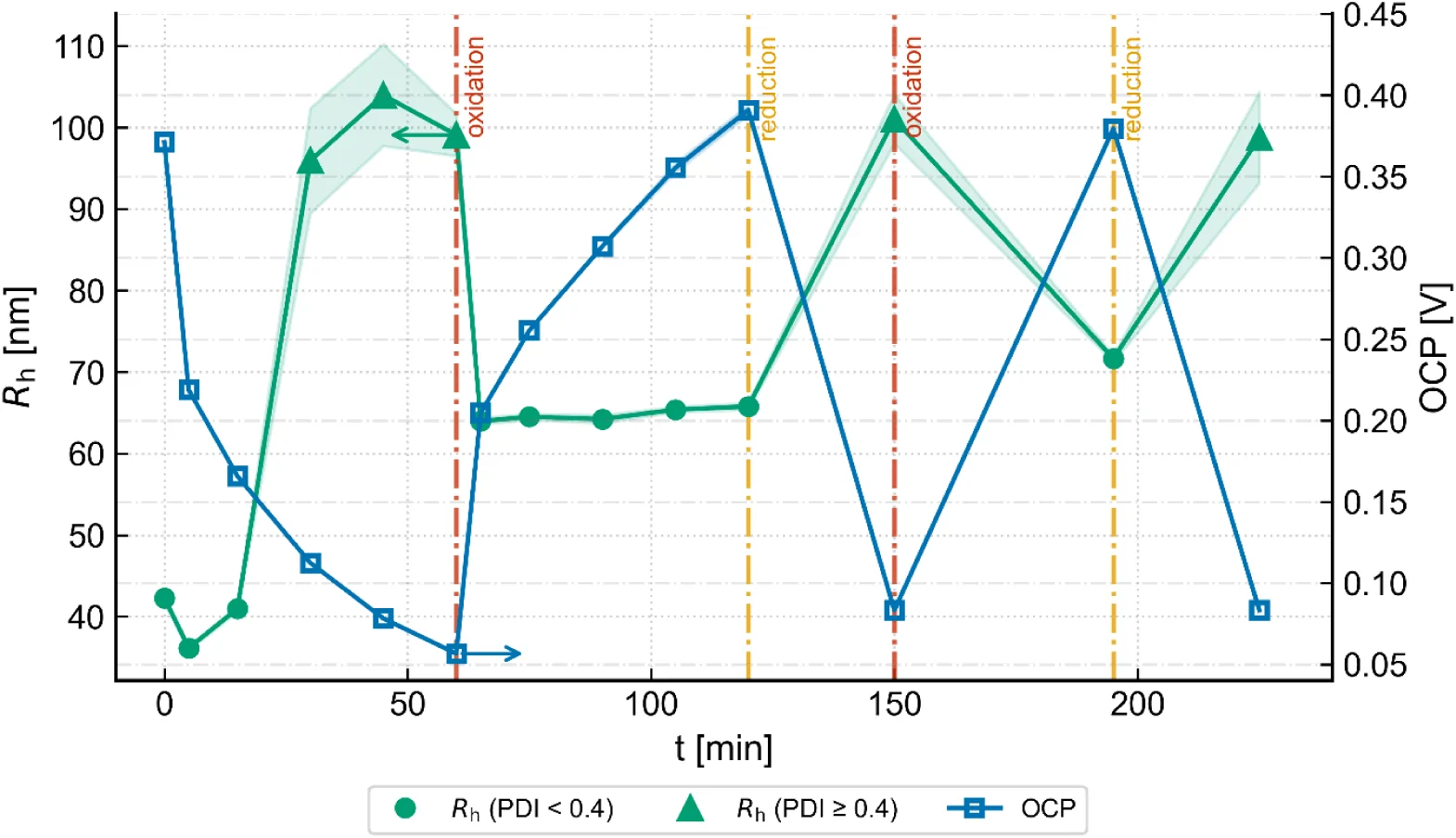
Electrochemical switching of the system containing PEO_114_-*b*-qPDPAEMA_171_ (0.2 g/L) and PAPMA_30_-*b*-PDMAA_120_ (1 g/L) in 0.1 M KCl at pH 4.7 (buffered with sodium acetate) with a starting concentration of 1.4 mM ferricyanide. At each point, the bulk electrolysis was paused to measure *OCP* (open symbols) and *R*_h_. Dots (•) represent *R*_h_ values extracted from cumulant analysis, and triangles (▲) represent values from regularized fitting (loose aggregates not shown), and the dashed-dotted lines indicate the times, when the bulk electrolysis was switched between oxidation and reduction. Shaded regions represent error bounds calculated from diffusion coefficient analysis across multiple scattering angles.

According to Faraday’s law, the complete oxidation/reduction of the system with the amount of moles (3.64 × 10^-5^ mol) of hexacyanoferrate involved requires approximately 3.5 C, which can be transferred within 30–60 min (sophisticated engineering of the electrochemical cell would surely reduce the switching times). As can be seen in Figure 15, this amount of charge was almost met by the first reduction, but not in subsequent reductions/oxidations. By using the Nernst equation, the approximate ratio of ferri-/ferrocyanide, which is also depicted in Figure 15, can be calculated from the measured *OCP*. Despite an incomplete reduction/oxidation, it becomes clear that there is at least 99% of ferricyanide present at the highest *OCP*s, while 99% of all available ferrocyanide is present at the lowest *OCP*s. Therefore, it can be concluded that the electrochemical experiments enable a full electrochemical modulation of the required change in the redox state of the system.

**Figure 15:**
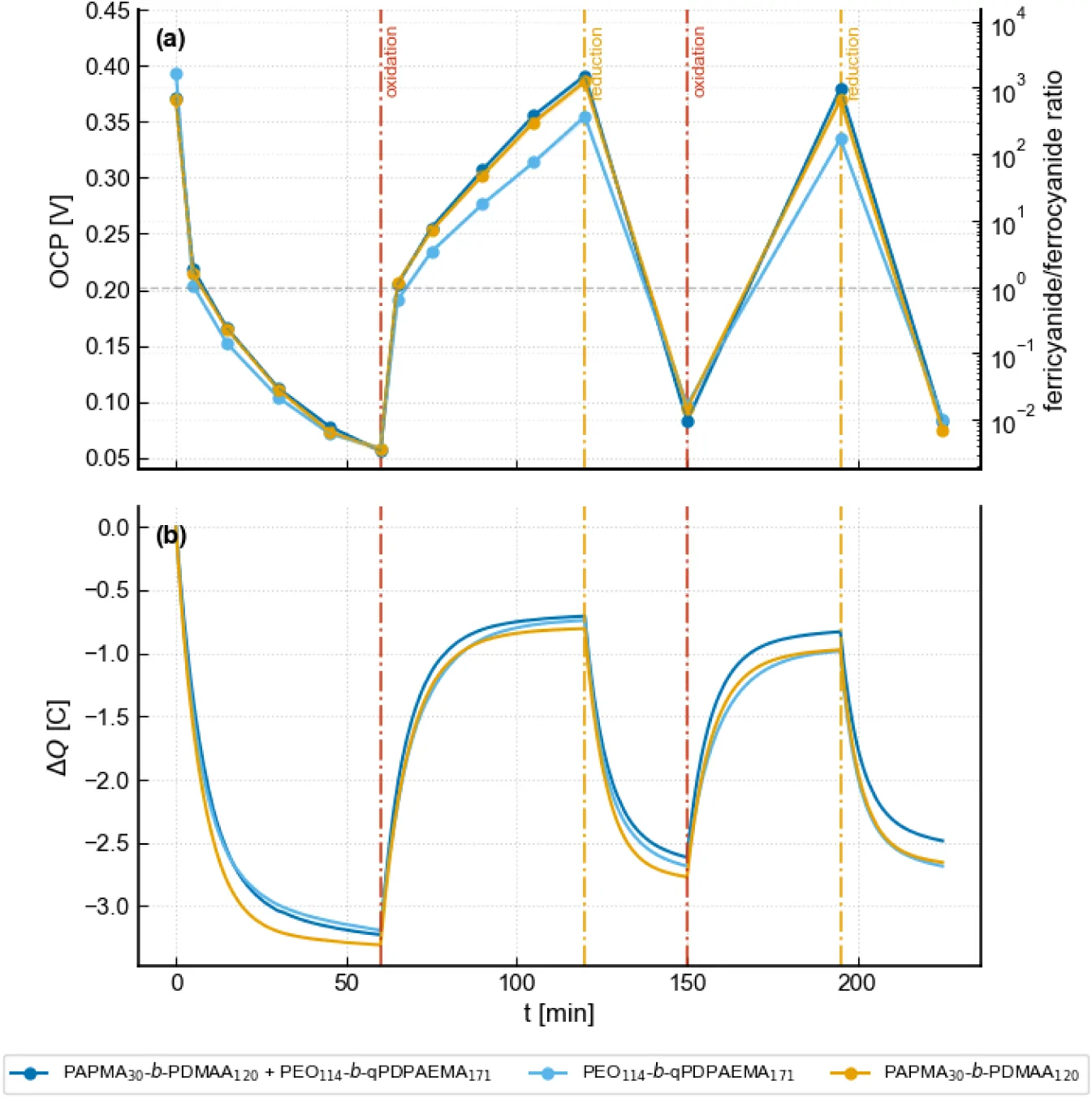
(top) Open circuit potential (*OCP*) over time during the bulk electrolysis: with the help of the Nernst equation, the ratio of ferri- to ferrocyanide is calculated, assuming a standard potential of 0.203 V. The latter is shown as a gray line, which was derived from cyclic voltammetry data (details in Section 2.2 in SI) of 1.4 mM ferricyanide in 0.1 M KCl solution (buffered at pH 4.7 with acetate). (bottom) Relative charge flow during the bulk electrolysis experiment. In the negative *y*-direction: reduction; in the positive *y*-direction: oxidation.

While switching is possible between states involving the extremes and even the mixed scenarios, the question arises whether a purely unimolecular state can also be achieved by electrochemistry. As mentioned before, PAPMA-*b*-PDMAA is a weak electrolyte, and a sufficient increase in pH should lead to negligible complexation. According to the literature,^61,62^ the p*K*_a_ of the APMA monomer is around 9, and a pH of approximately 11 results in almost completely deprotonated APMA units, which mitigates any complexation with the anionic metalate ions.

In fact, this pH change can be achieved electrochemically. By applying a potential of 1 V during the bulk electrolysis, the pH can be effectively increased by water electrolysis, even for the buffered system. This procedure was tested with the solution shown in Figure 14 after the measurement, which resulted in conditions of pH 11.2 with completely reduced ferricyanide. Under these conditions, aggregation should be prevented, and indeed, unimers were predominantly found via DLS (details Section 2.7 in SI).

To demonstrate the full control and accessibility of this approach, the combined system was subsequently subjected to a complete electrochemical cycle. Starting at 1.4 mM ferricyanide (step 0, *R*_h_ ≈ 43 nm, pH 4.7; compare also Figures 12 and 14), electrochemical reduction converts all ferricyanide to ferrocyanide, which in turn triggers PAPMA-*b*-PDMAA aggregation (step 1, *R*_h_ ≈ 186 nm). Electrochemical pH increase to 11.1 then dissolves all aggregates into the unimolecular state (step 2), after which reoxidation at elevated pH produces PEO-*b*-qPDPAEMA micelles close to equilibrium (step 3, *R*_h_ ≈ 53 nm), being considerably larger than the crew-cut micelles in their nonequilibrium state. These micelles apparently persist upon electrochemical acidification to pH 4.4 (step 4, *R*_h_ ≈ 67 nm) by applying a voltage of 1.3 V, resembling perfectly the vesicles obtained after the electrochemical oxidation in Figure 8. Hence, we were able to complete a full redox cycle by passing through all four distinct states, though the delicate comicellar state (with the narrow window of existence seen in Figure 12) is hard to detect by this method. From the completely oxidized state, the state of PAPMA-*b*-PDMAA aggregation can be established again by reduction (repetition of step 1, *R*_h_ ≈ 118 nm). More details of these results can be found in the SI in Section 2.7. However, the repeated pH excursions between low and high pH required for full four-state control are expected to limit long-term cyclability, as successive cycles at high pH might progressively compromise the chemical stability of the polymers.

In sum, we demonstrate full (electro)­chemical control over all four accessible aggregation states of the combined polymer system through modulation of both the hexacyanoferrate redox ratio and the solution pH.

## Conclusions

In this work, we demonstrated electrochemically controlled micellization of two amphiphilic block copolymers, PAPMA_30_-*b*-PDMAA_120_ and PEO_114_-*b*-qPDPAEMA_171_ by redox switching of hexacyanoferrate ions. The polymers exhibit complementary behavior: while PAPMA-*b*-PDMAA aggregates with ferrocyanide, PEO-*b*-qPDPAEMA aggregates with ferricyanide in the chosen concentration range, attributable to differences in ion polarizability and hard/soft acid–base interactions.^66^

Both systems can be reversibly switched between unimolecular and aggregated states through chemical or electrochemical oxidation or reduction. For PEO-*b*-qPDPAEMA, the pathway matters. While chemical oxidation produces compact spherical micelles, electrochemical treatment yields larger vesicular structures, suggesting kinetic versus thermodynamic control of assembly.

When combined, the two polymers enable complete control over the aggregation characteristics of this complex mixture because (electro)­chemical modulation allows selective switching between PAPMA-*b*-PDMAA aggregates, PEO-*b*-qPDPAEMA vesicles, mixed aggregates, or fully unimolecular states (the latter achieved by electrochemically raising the pH). This represents a powerful approach for the dynamic control of polymer self-assembly using a single externally tunable stimulus: the applied electrode potential.

There is a plethora of future potential in incorporating electroactive groups into polymers, which can become hydrophilic/hydrophobic on demand, opening opportunities for responsive materials, controlled delivery systems, and adaptive soft matter applications.

## Experimental Section

### Materials

*N*-(3-*t*-Boc-aminopropyl)­methacrylamide (≤100%) was purchased from Polysciences (Warrington, USA); ethanol (99.8%) and dimethylacetamide (HPLC-grade) from Fisher Scientific (Waltham, USA); 2-cyano-2-propyldodecyltrithiocarbonate (CPDC, 97%), 4,4′azobis­(4-cyanopentanoic acid) (ACVA, > 98%), potassium hexacyanoferrate­(II) trihydrate, and *N*,*N*-dimethylacrylamide (99%) from Sigma-Aldrich (Darmstadt, Germany); potassium hydroxide (98.4%) and 37% hydrochloric acid from VWR (Darmstadt, Germany); potassium hexacyanoferrate­(III) from VEB Laborchemie Apolda (Apolda, Germany); potassium chloride (>99.5%) from Chemsolute (Renningen, Germany); H_2_O_2_ (synthesis grade) from Carl Roth (Karlsruhe, Germany); ascorbic acid (99+%) from Alfa Aesar (Haverhill, USA); and 1,4-dioxane (≤99.5%) from Grüssing (Filsum, Germany). Isopropanol and trifluoroacetic acid were redistilled from technical grade. A Merck (Darmstadt, Germany) Milli-Q IQ 7000 device provided purified water for the preparation of samples. The dialysis membranes used were Spectra/Por Biotech Membranes (1 kDa, 6–8 kDa, and 100 kDa) made from either regenerated cellulose (RC) or cellulose ester (CE), manufactured by Spectrum Laboratory Products (New Brunswick, USA). The basic aluminum oxide (Alox B, for chromatography, Brockmann I, 40–300 μm, 60A) was purchased from Acros Organics (now part of Fisher Scientific).

### Synthesis

#### Poly­[*N*-(*t*-Boc-Aminopropyl)­methacrylamide] (**A**)

1.067 g (4.409 mmol) *N*-(*t*-Boc-aminopropyl)­methacrylamide [*t-*Boc­(APMA)] was dissolved in 1.5 g ethanol (EtOH) in a glass vial and purged with nitrogen for 30 min. To this mixture, a solution of 28.6 mg (8.29 × 10^–2^ mmol) CTA (2-cyano-2-propyldodecyltrithiocarbonate) and 2.6 mg (9.28 × 10^–3^ mmol) 4,4′-azobis­(4-cyanopentanoic acid) (ACVA) in 0.5 g EtOH was added [rough ratio monomer/CTA/initiator: 50/1/0.1]. The whole mixture was then again purged with nitrogen for 30 min. Subsequently, the flask was immersed in a preheated oil bath at 70 °C and stirred for 19 h at a temperature of 68 °C. The reaction was then terminated by cooling in an ice bath and exposure to air. The product was purified by dialysis against 1,4-dioxane (MWCO: 1 kDa, RC). Subsequent freeze-drying yielded 0.5925 g of a white powder (57% conversion according to NMR). Characterization details are found in the SI (Section 1.1.1).

#### Poly­[*N*-(*t*-Boc-Aminopropyl)­methacrylamide-*b*-*N*,*N*-dimethylacrylamide] (**B**)

0.3691 g (4.85·10^–2^ mmol) of **A** was dissolved in about 2 g of ethanol. Meanwhile, the inhibitor was removed from the monomer *N*,*N*-dimethylacrylamide (DMAA) using a column of basic aluminum oxide (Alox B). To the ethanolic solution, 0.6045 g (6.094 mmol) of monomer and 1.8 mg (3.85·10^–3^ mmol) of ACVA were added and purged with nitrogen for 30 min [approximate ratio monomer/CTA/initiator: 125/1/0.1]. The mixture was placed in a preheated oil bath at 70 °C and stirred for 5 h. The reaction was terminated by placing the reaction tube in an ice bath and exposing it to air. The polymer was purified by dialysis against 1,4-dioxane (MWCO: 1 kDa, RC) and freeze-dried, yielding 0.7297 g of a white powder (89% conversion according to NMR). Further details can be found in the SI (Section 1.1.2).

#### Poly­[(*N*-aminopropyl)­methacrylamide-*b*-*N*,*N*-dimethylacrylamide] (**C**)

300 mg (1.51 × 10^–2^ mmol) of **B** were dissolved in approximately 5 mL of trifluoroacetic acid (TFA) and stirred at room temperature for 48 h. Excess TFA was removed in vacuo, and the residue was neutralized with a 4 M potassium hydroxide solution. The product was then purified by aqueous dialysis (MWCO: 6–8 kDa, RC) at a pH of 4–5 (acidified with hydrochloric acid). Subsequent freeze-drying yielded 254 mg of a white solid. Further NMR details can be found in the SI (Section 1.1.3).

### Methods

#### Size Exclusion Chromatography (SEC)

Size-exclusion chromatography (SEC) measurements in dimethylacetamide (DMAc) were conducted on a PSS SECurity^2^ system consisting of a PSS SECurity 2-Canal Inline Degasser, a PSS SECurity^2^ TCC6500 column oven (50 °C), a PSS GRAM column set (8 × 50 mm, 10 μm precolumn, 8 × 300 mm, 10 μm analytical column), a PSS SECurity^2^ isocratic pump, a PSS SECurity^2^ automatic vial sampler, and a PSS SECurity^2^ refractive index detector (35 °C). High-performance liquid chromatography (HPLC)-grade DMAc was used as an eluent at a flow rate of 1 mL min^–1^. All samples were prepared at concentrations of 1 mg mL^–1^ (in DMAc) and passed through 0.22 μm polytetrafluoroethylene (PTFE) membrane filters. Molecular weight and dispersity analyses were evaluated using PSS WinGPC UniChrom software (version 8.3). The system was calibrated using a PSS ReadyCal Kit PEG/PEO (238–9.69 × 10^5^ g mol^–1^).

#### Nuclear Magnetic Resonance Spectroscopy (NMR) and Diffusion-Ordered NMR Spectroscopy (DOSY)

The NMR spectra of the final and intermediate products were measured with a Bruker Avance III 500 MHz NMR spectrometer (Bruker BioSpin, Ettlingen, Germany) in either D_2_O or CDCl_3_ and evaluated with the corresponding Bruker software, TopSpin (version 4.0.8). Chemical shifts are given in ppm and are either relative to the residual HDO signal of D_2_O at δ = 4.79 ppm or SiMe_4_ (internal standard in CDCl_3_).

Diffusion-ordered spectroscopy (DOSY) was carried out on ^1^H channel using the same spectrometer, with sample concentrations of 8.5 mg/mL in D_2_O. Measurements were performed at 298.2 K using the bipolar gradient pulse sequence ledbpgp2s. The diffusion time *Δ* was set to 0.2 s and the gradient pulse duration to δ = 3 ms. A series of 16 gradient increments, ranging linearly from 2% to 95% of the maximum gradient strength, was applied, and 16 scans were accumulated for each increment. The spectra were processed with TopSpin (version 4.0.8) using a line-broadening of 1 Hz. The diffusion coefficients were determined using the DOSY evaluation subroutine in TopSpin via an exponential fitting of the signal integrals with one component.

#### Small-Angle X-ray Scattering (SAXS)

Small-angle X-ray scattering (SAXS) measurements were performed using a SAXSpoint 5.0 system (control software: SAXSdrive 4.60.1.734) from Anton Paar (Graz, Austria). The system utilized a Cu K_α_ source (λ = 0.154 nm) and a pinhole collimation setup (achieved by an octagonal slit collimation system). The beam was collimated to a diameter of 0.3 mm before the sample position. The scattered intensity was recorded on a Dectris Eiger2 1M detector (75 μm pixel size). To cover a wide range of the scattering vector, data were collected at multiple detector positions with a fixed sample-to-detector distance of 1.6 m. The acquisition time for each detector position was 2 h, collected as 24 individual frames of 300 s each, resulting in a total acquisition time of 8 h per sample. All measurements were conducted using flame-sealed glass capillaries (borosilicate glass, 0.01 mm wall thickness, manufacturer: WJM Glas) at a constant temperature of 20 °C using temperature-controlled sample stages. The radially averaged 1D intensities are provided. The data were fitted using the software SasView^73^ (version 6.1.0), whereas details about the fitting process can be found in the SI (Sections 1.2.1 and 2.10).

#### Cryogenic Transmission Electron Microscopy (Cryo-TEM)

Cryo transmission electron microscopy (cryo-TEM) was performed on a JEM-2200FS from JEOL (Akishima, Japan), operating at 200 kV and equipped with a field emission gun and an in-column energy omega filter. The zero-loss energy-filtered, bright-field micrographs were recorded under cryo conditions (Holder *T* = −174 °C) with a bottom-mounted CMOS 4K camera (OneView) by Gatan (Pleasanton, USA). The images were processed using digital processing software (Digital Micrograph 3.6, Gatan). For cryo preparation, 4 μL of the aqueous sample solution was deposited on either a copper grid with a holey carbon film from Quantifoil (Großlöbichau, Germany) or a gold grid with a lacey carbon film from Electron Microscopy Sciences (Hatfield, USA). In both cases, the support grids were glow-discharged with an air plasma beforehand. The grid was then blotted at 15 °C and 90% humidity with filter paper and plunged into liquid ethane using a Leica EM GP plunge freezer (Wetzlar, Germany). After preparation, the grids were stored and transferred to the TEM under liquid nitrogen.

#### Electrochemical Measurements

Electrochemical measurements were performed with a CHI760E potentiostat from CH Instruments (Dallas, USA) using a three-electrode setup, with a platinum wire (23 cm, 0.5 mm diameter) as the counter electrode and an Ag/AgCl electrode (type RE-1B) as the reference electrode. For the bulk electrolysis (BE) experiments, a platinum gauze electrode (80 mesh, 25 × 25 mm) was used as the working electrode, while for the cyclic voltammetry experiments, a round Pt electrode (radius 2 mm) was employed. All electrodes were purchased from ALS (Tokyo, Japan). Further details about the setup and experiments can be found in the SI (Section 1.2.2).

#### Dynamic Light Scattering (DLS)

Dynamic light scattering (DLS) measurements were performed with an ALV/CGS-3 Goniometer system (Langen, Germany) equipped with a He–Ne laser (32 mW, 632.8 nm), two avalanche photodiodes, an external programmable thermostat (Julabo F32), and an index-matching bath filled with toluene. Angle-dependent measurements were performed at 20 °C in a pseudo cross-correlation mode, varying the scattering angle from 50° to 150° in 10° steps, 3-fold, with a duration of 12 s for each run. The resulting data were evaluated with the software JADE-DLS.^63^ Further details are found in the SI (Section 1.2.3)

## Supplementary Material



## Data Availability

All figures and relevant measurement data are available via zenodo: 10.5281/zenodo.20232735 (https://zenodo.org/records/20232735).

## Notes

Experimental details on synthesis and characterization; detailed discussion on the synthesis procedure; further electrochemistry data, scattering data, and cryo-TEM images (PDF)
