# Supplementary material for: Full (Electro-)Chemical Control over the Aggregation State of Diblock Copolymer Mixtures Including Nonequilibrium Micelles
Source: ACS Polym Au. 2026 Jun 1;6(5):1267–85. doi: 10.1021/acspolymersau.6c00044 (PMC13673910; doi:10.1021/acspolymersau.6c00044)
Supplement: Supplementary file 1 [file lg6c00044_si_001.pdf]

Full (Electro-)Chemical Control over the Aggregation State of Diblock Copolymer Mixtures Including Nonequilibrium Micelles

-

SUPPORTING INFORMATION

Vincent Schildknecht[1], *Annika Lehmann*[1], *Richard Neubert*[1], *Quirin Prasser*[1], *Anja Voigt*[1], *Gilbert Chahine*[2], *Isabelle Morfin*[3], *Erica Brendler*[4], *Sabine Rosenfeldt*[5], *Ulrich Mansfeld*[6], *Felix A. Plamper*[1,7,8*]

[1] Institute of Physical Chemistry, TU Bergakademie Freiberg, Lessingstr. 45, 09599 Freiberg, Germany.

[2] CNRS, Grenoble-INP, SIMAP, Université Grenoble Alpes, 38000 Grenoble, France.

[3] CNRS, LiPhy, Université Grenoble Alpes, 38000 Grenoble, France.

[4] Institute of Analytical Chemistry, TU Bergakademie Freiberg, Lessingstr. 45, 09599 Freiberg, Germany.

[5] Bayreuth Institute of Physical Chemistry I and Bavarian Polymer Institute (BPI), University of Bayreuth, Universitätsstr. 30, 95447 Bayreuth, Germany.

[6] Bayreuth Institute for Macromolecular Research (BIMF) and Bavarian Polymer Institute (BPI), University of Bayreuth, Universitätsstr. 30, 95447 Bayreuth, Germany.

[7] Center for Efficient High Temperature Processes and Materials Conversion ZeHS, TU Bergakademie Freiberg, Winklerstr. 5, 09599 Freiberg, Germany

[8] Freiberg Center for Water Research ZeWaF, TU Bergakademie Freiberg, Winklerstr. 5, 09599 Freiberg, Germany.

* felix.plamper@chemie.tu-freiberg.de

1. EXPERIMENTAL

1.1 Synthesis

1.1.1 Poly[*N*-(*t*-Boc-aminopropyl)methacrylamide] (A)

Polymer **A** was synthesized as described in the main section. The ^{1}H-NMR of the purified polymer **A** is depicted in Fig. S1. The polymer was further characterized with Size Exclusion Chromatography (SEC; $Đ$ = 1.23; M_n = 5080, PEO-calibrated). The prominent impurity at a chemical shift of approximately 3.7 ppm can be assigned to 1,4-dioxane left from freeze drying.[1]

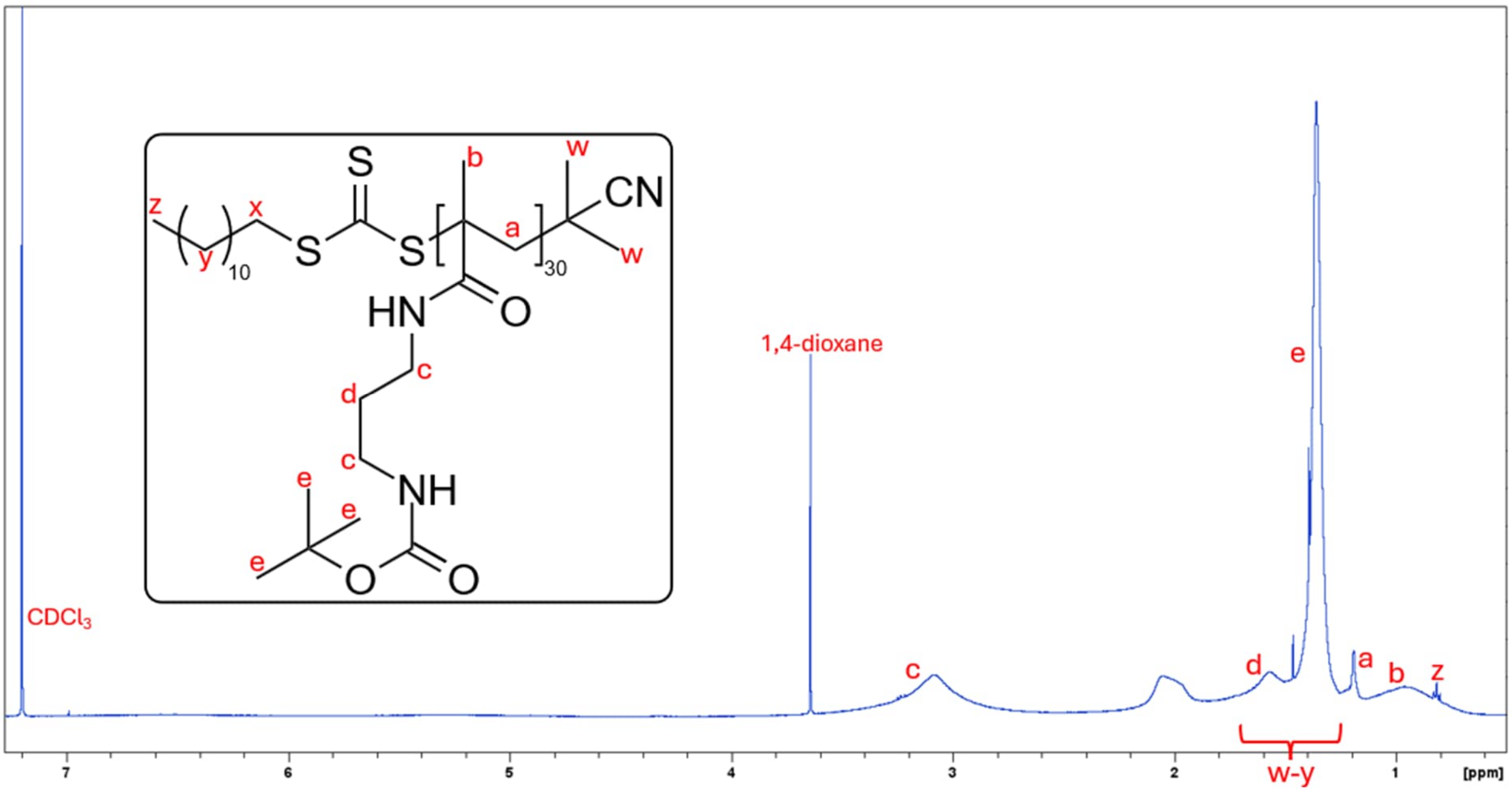

Fig. S1: ^{1}H-NMR of the P(*t*-Boc)APMA$_{30}$ macro-CTA in $CDCl_3$ (NH-signal not visible, probably due to exchange broadening).

1.1.2 Poly[*N*-(*t*-Boc-aminopropyl)methacrylamide-*b*-*N*,*N*-dimethylacrylamide] (B)

Polymer **B** was synthesized as described in the main section. According to SEC, there was still a non-negligible amount of **A** in the product, which was removed by multiple dialysis steps, each over 72h in isopropanol (MWCO: 100 kDa, CE), to gain the purified diblock-copolymer with its ^{1}H-NMR shown in Fig. S2. To determine the molar ratio of the P(*t*-Boc)APMA block (mol% = 0.21), the intensities of signals e and B, as well as b and B were compared in respect to their proton count. Also, SEC of the pure diblock was carried out ($Đ$ = 1.27; M_n = 14300, PEO-calibrated).

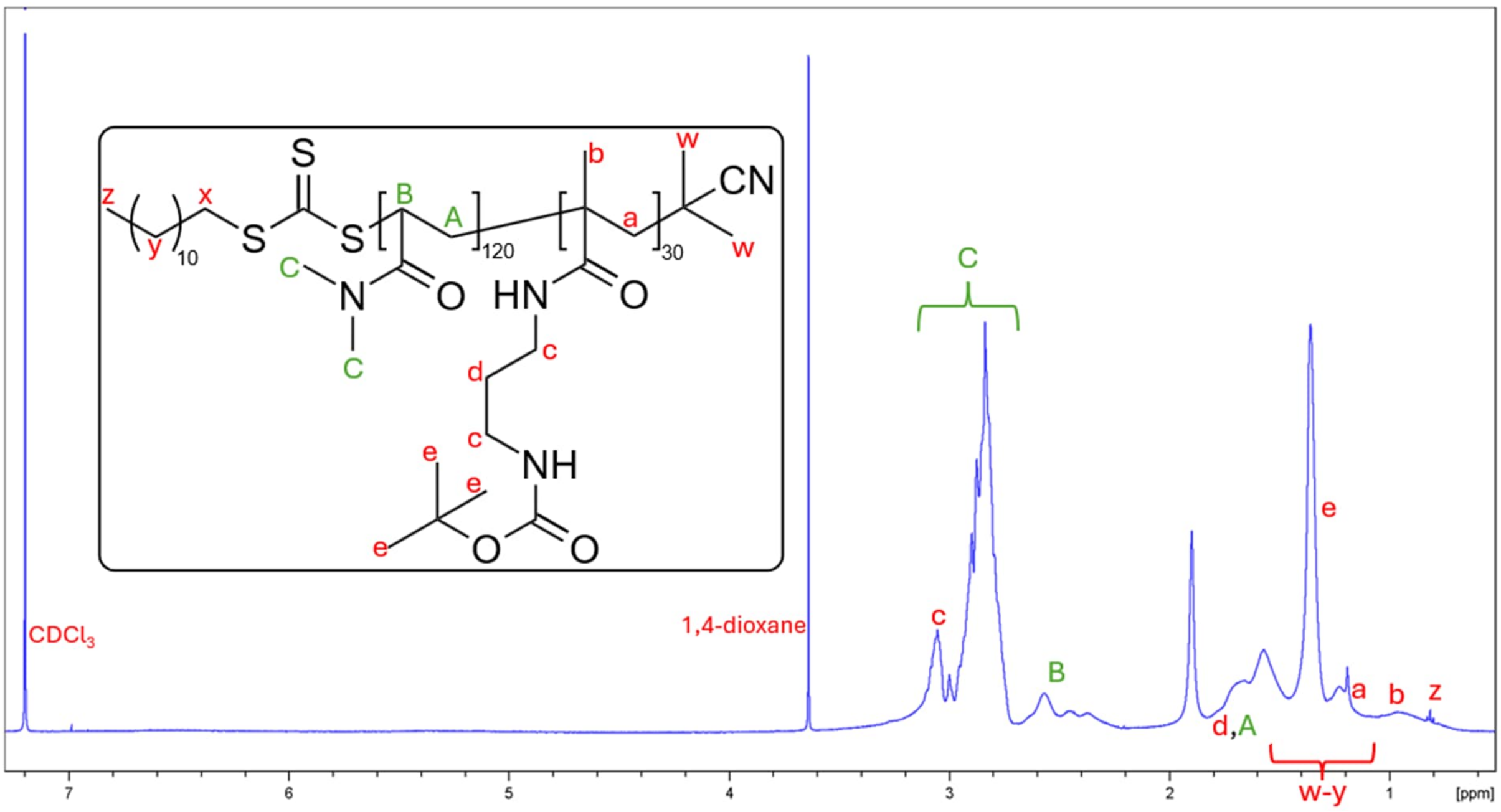

Fig. S2: ^{1}H-NMR of diblock copolymer P(*t*-Boc)APMA$_{30}$-*b*-PDMAA$_{120}$ in $CDCl_3$ (NH-signal not visible, probably due to exchange broadening).

1.1.3 Poly[(*N*-aminopropyl)methacrylamide-*b*-*N*,*N*-dimethylacrylamide] (C)

Polymer **C** was synthesized as described in the main section. The ^{1}H-NMR is depicted in Fig. S3. Here, the molar ratio of the PAPMA block (mol% = 0.19) is determined by comparing the intensities of signals b and B in respect to their proton count.

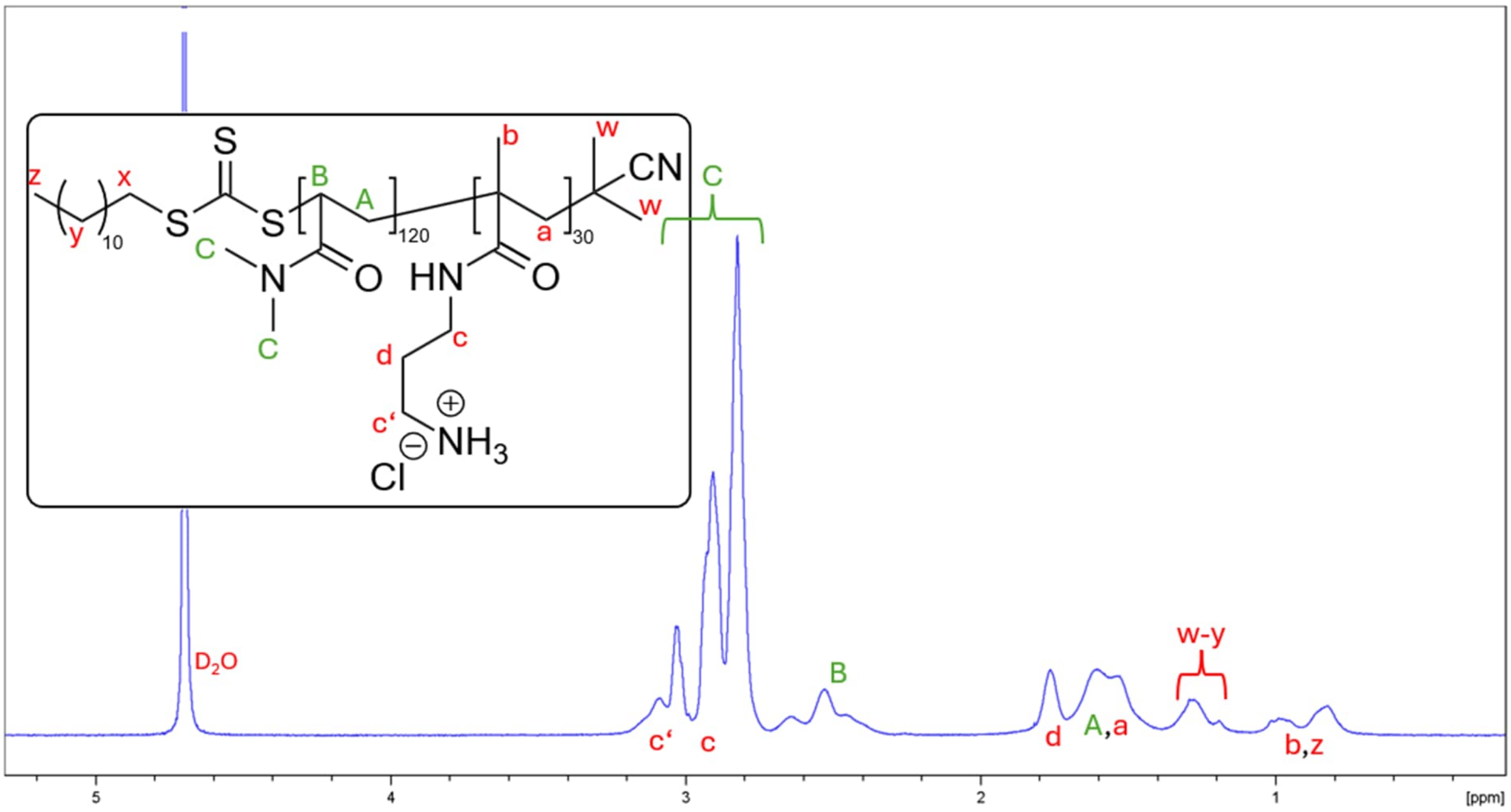

Fig. S3: ^{1}H-NMR of diblock copolymer PAPMA$_{30}$-*b*-PDMAA$_{120}$ in D_2O.

1.1.4 Poly[(*N*-aminopropyl)methacrylamide]

150 mg ($1.97\cdot10^{-2}$) of **A** were dissolved in about 3 ml of trifluoroacetic acid (TFA) and stirred at room temperature for 25 h. Excess TFA was removed in vacuo and the residue neutralized with 4 M potassium hydroxide solution. The product was then cleaned by aqueous dialysis (MWCO: 1 kDa, RC) at a pH of 3.5 (acidified with hydrochloric acid). Subsequent freeze-drying yielded 143 mg white solid. The ^{1}H-NMR is depicted in Fig. S4.

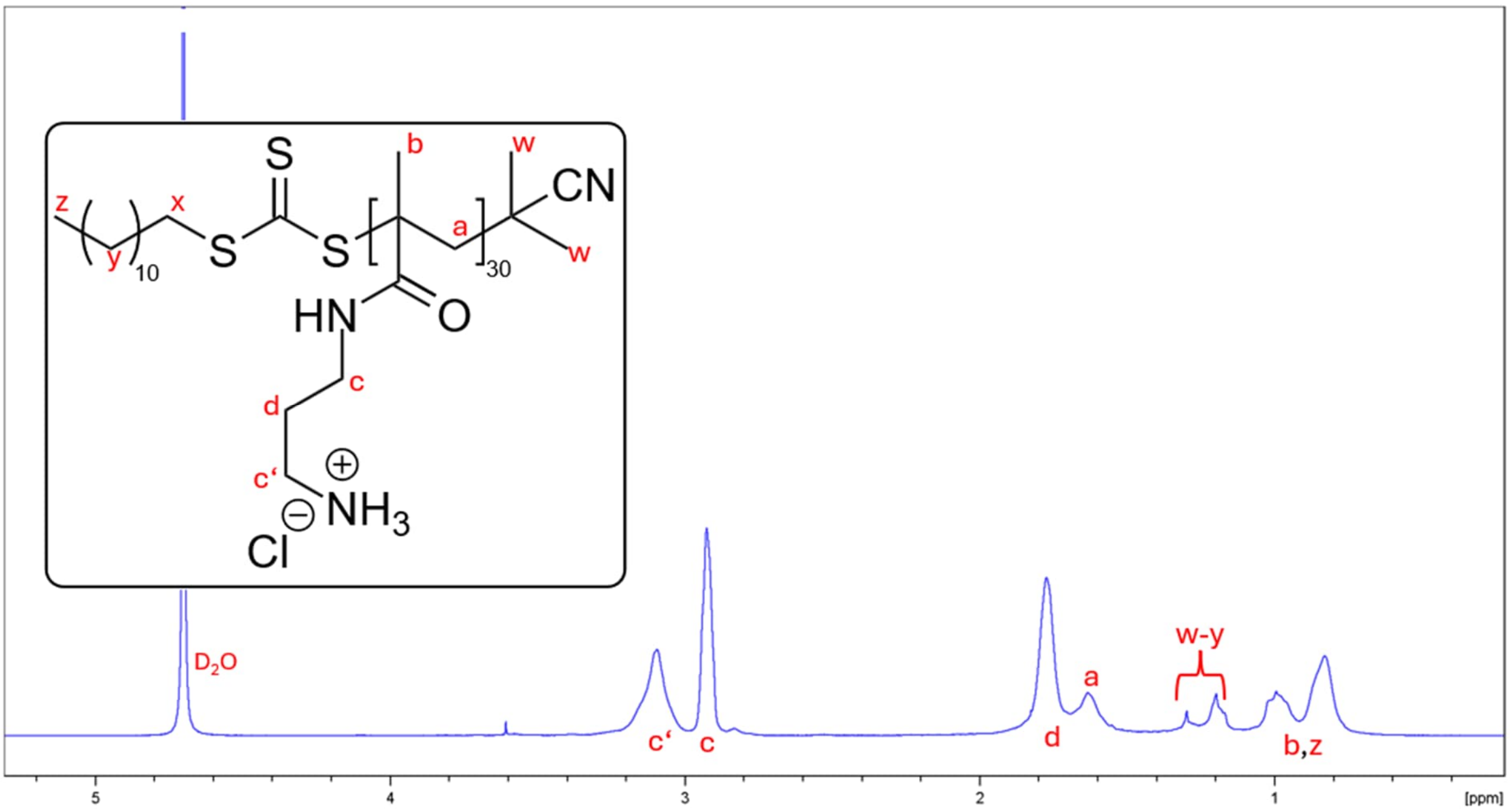

Fig. S4: ^{1}H-NMR of deprotected macro-CTA diblock $PAPMA_{30}$ in D_2O.

1.2 Methods

1.2.1 Small Angle X-Ray Scattering (SAXS)

Higher concentrated samples than the ones showed in the main section were investigated at the European Synchrotron Radiation Facility (ESRF) at the Beamline 2 (BM02) (data: *doi.org/10.15151/ESRF-ES-2049617513*). Here, each sample was measured at 7123.5 eV with an acquisition time of 60 s in 1s steps using a Pixel Photon Count Detector IMXPAD d5-S540. All measurements were conducted using flame-sealed glass capillaries (boro silicate glass, 0.01 mm wall thickness, manufacturer: WJM Glas) at a constant temperature of 20 °C using temperature-controlled sample stages. The radially averaged 1D intensities are given.

The data were fitted using the software SasView[2] (version 6.1.0) and the following form factors were used when fitting the SAXS data. The sphere formfactor[3] is defined as follows:

$$I(q) = \frac{scale}{V}\left[3V(\Delta\rho)\cdot\frac{sin(qr) - qrcos(qr)}{(qr)^3}\right]^2 + background \tag{1}$$

Whereas V is the volume of the scatterer, r the radius of the sphere and $\Delta\rho$ the difference between SLDs of scatterer and solvent. The formfactor for the hollow sphere[3] is defined as:

$$I(q) = \frac{\varphi}{V_{shell}}\left[\frac{3V_{core}(\Delta\rho)j_1(qr_{core})}{qr_{core}} + \frac{3V_{tot}(\Delta\rho)j_1(qr_{tot})}{qr_{tot}}\right]^2 + background \tag{2}$$

Here, φ is the volume fraction and *tot* corresponds to the total scattering object, while *core* only corresponds to the core of the scatterer. j_1 is the spherical Bessel-function:

$$j_1 = \frac{\big(sin(x) - xcos(x)\big)}{x^2} \tag{3}$$

1.2.2 Electrochemical Measurements

The electrochemical cell for the electrochemical measurements was custom made at the TU Bergakademie Freiberg consisting of a main chamber for the working and reference electrode and a separate cell for the counter electrode connected to the main chamber with a glass frit, filled with 3 M KCl-solution (s. Fig. S5). The open circuit potential (OCP) was measured for 60 seconds and averaged over that time. Bulk electrolysis (BE) was conducted by applying a voltage of -0.1 V for reduction and 0.5 V for oxidation while stirring and purging with nitrogen. Cyclic voltammetry (CV) was conducted between 0.5 V and -0.1 V, starting at 0.5 V, with scanning speeds of 10, 20, 50 and 100 mV/s. The number of cycles per measurement was 6 for 10 mV/s and 3 for 20, 50 and 100 mV/s.

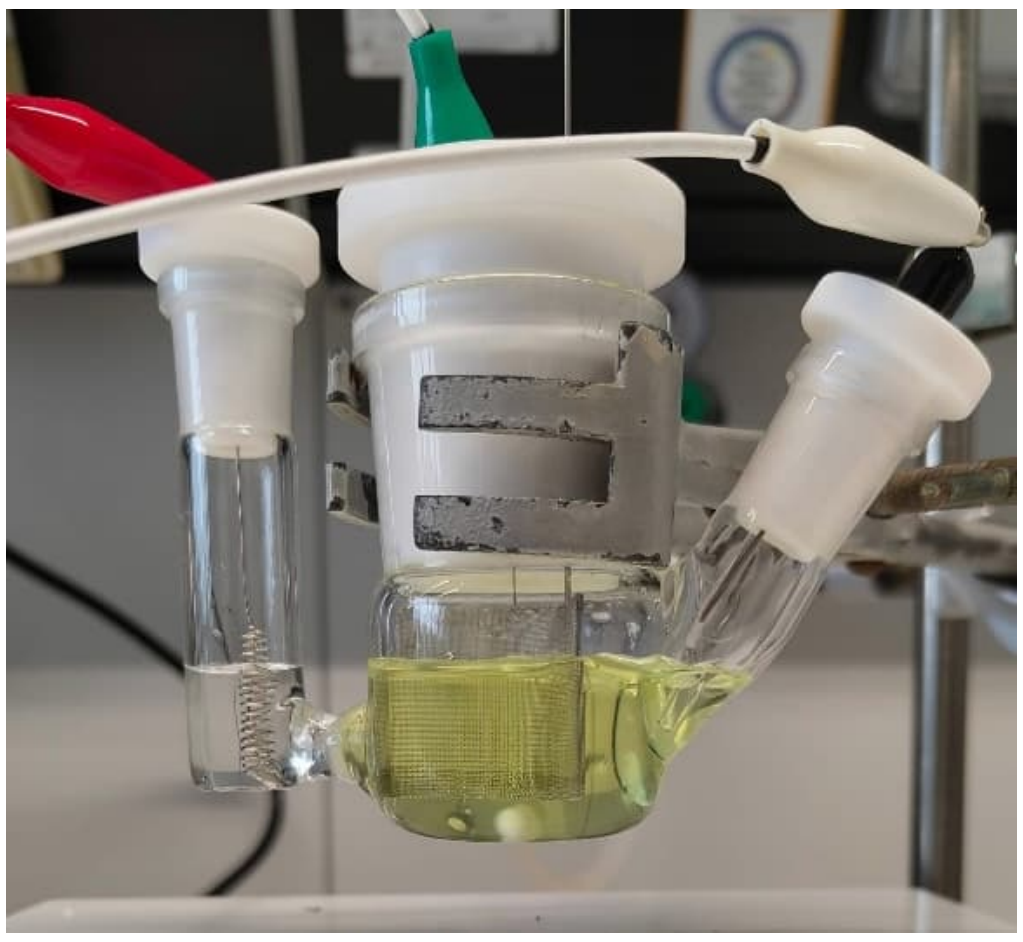

Fig. S5: Picture of the electrolysis cell in use. On the left a separate chamber for the counter electrode filled with 3 M KCl, on the right Ag/AgCl-reference electrode and in the middle the platinum mesh used as working electrode dipped into the yellowish ferricyanide solution purged with nitrogen. During electrolysis, the solution is stirred with a magnetic stirrer.

1.2.3 Dynamic Light Scattering (DLS)

As mentioned, the data were evaluated with the software JADE-DLS (version 1; https://github.com/AG-Plamper/JADE-DLS).[4] In every case, the hydrodynamic radius was calculated using the Stokes-Einstein equation from the diffusion coefficient determined by linear regression of the decay rate versus the squared scattering vector. The decay rates were determined by the cumulant method (up to 4th cumulant), using an iterative fitting method, and regularized fitting to check for multimodality. In case of multimodal samples, the diffusion coefficient of each mode was determined individually by linear regression. The smoothing factor α in regularized fitting was determined for each set of measurements (each determination of R_h) individually by varying α (between 0.1 and 1) for randomly chosen sets of measurements inside one measurement (5 different angles) and then choosing a physically meaningful α. Details about the exact processing can directly be found in the code of the software. The plotted intensities were calculated by the following equation:

$$I = \frac{CR_{mean}}{I_{Laser}} \cdot sin(\theta) \tag{4}$$

Here, θ is the scattering angle, I_{Laser} the intensity of the laser beam (measured by splitting the primary beam towards a photodiode) and CR_{mean} the mean countrate over the measurement duration.

2. FURTHER RESULTS AND DISCUSSIONS

2.1 Detailed Synthesis Discussion

As mentioned in the main part, several problems were encountered during RAFT synthesis of the diblock polymer PAPMA-*b*-PDMAA. First attempts of synthesizing a PAPMA-macroinitiator failed because of solubility issues either with the monomer in organic solvents or a suitable chain transfer agent (CTA) in aqueous solution, while synthesizing the PAPMA-block onto the PDMAA-macroinitiator lead to issues during macromolecular characterization of the final P(APMA-*b*-DMAA) in typical eluents, like dimethylacetamide, leading to tailing in Size Exclusion Chromatography (SEC), even when optimizing the conditions for one of the blocks. As a workaround, the *tert*-butyloxycarbonyl (*t*-Boc) protecting group led to *N*-(3-*t*-Boc-aminopropyl)methacrylamide as a proteced APMA monomer, which was used for further synthesis.

Two different CTAs were tested for synthesis. While CPCP (4-cyano-4-(phenylcarbonothioyl-thio)pentanoic acid) showed poor results, 2-cyano-2-propyldodecyltrithiocarbonate (CPDC) lead to successful polymerization in ethanol. The conversion ranged around 55% (as an average over three different attempts), indicating that system-specific optimization could unlock even greater efficiency gains. The second block consisting of *N*,*N*-dimethylacrylamide was then also added in ethanol in a similar procedure. After the addition of the second block (94% conversion in average), a non-negligible amount of homopolymer documents a nonquantitative blocking efficiency. One reason appears to be the fundamentally different reaction kinectics of both monomers, since one monomer is a methacrylamide and the other one is an acrylamide.

While both acrylamides and methacrylamides are considered as more activated monomers (MAMs),[5] which are characterized by their electron-withdrawing substituents that activate the vinyl double bond towards radical addition, acrylamides are more reactive in propagation reactions than methacrylamides, as their less stabilized radicals exhibit higher addition rate coefficients.[5] Methacrylamides are 1,1-disubstituted monomers bearing two substituents on the α-carbon, whereas acrylamides are monosubstituted with only one α-substituent. This structural difference profoundly affects radical stability, because the additional α-substitution in methacrylamides generates more sterically hindered and resonance-stabilized propagating radicals. Despite their lower propagation reactivity, 1,1-disubstituted monomers exhibit favorable behavior in RAFT polymerizations because of their performance when being incorporated in direct proximity to the sulfur-containing moiety of the CTA: the disubstitution

stabilizes the propagating radical and makes the corresponding RAFT-derived polymer radical a better leaving group during the fragmentation step of the RAFT equilibrium. This enhanced fragmentation efficiency compensates for the slower monomer addition, maintaining rapid RAFT equilibrium and preventing the retardation that can plague polymerizations with slow fragmentation kinetics.[6] In contrast, monosubstituted radicals, while faster propagating species, are less effective leaving groups in the RAFT mechanism. This principle is the main reason for the necessity of polymerizing the 1,1-disubstituted monomer block first to serve as macro-CTA for the subsequent polymerization of the monosubstituted monomer during synthesis of block copolymers combining these two classes of monomers. Reversing this order results in inefficient reinitiation, as shown for different block compositions of polyacrylamide and polymethacrylamide by Y. Vasilieva et al.[7] The initially extended blocks grow even further because of the stabilized radicals involved, while the still non-extended homopolymers hardly experience any fragmentation anymore because of the higher energy of the resulting fragmented radicals. Despite these principles, the blocking efficiency is also not perfect in our case, as some homopolymer is left even after extension.

2.2 Evaluation of CV-Data

To determine the standard potential [($E_{cathodicPeak}$+$E_{anodicPeak}$)/2] versus the Ag/AgCl-reference electrode of the ferro-/ferricyanide-redox couple, CV-measurements of a 1.4 mM ferricyanide solution (in 0.1 M KCl and buffered with actetate at pH 4.7) were conducted. As a result over multiple cycles and scan rates a value of approximately 0.203 V was calculated (against Ag/AgCl; Fig. S6). The curves are as expected and show symmetric oxidation and reduction peaks with increasing current for higher scan rates.

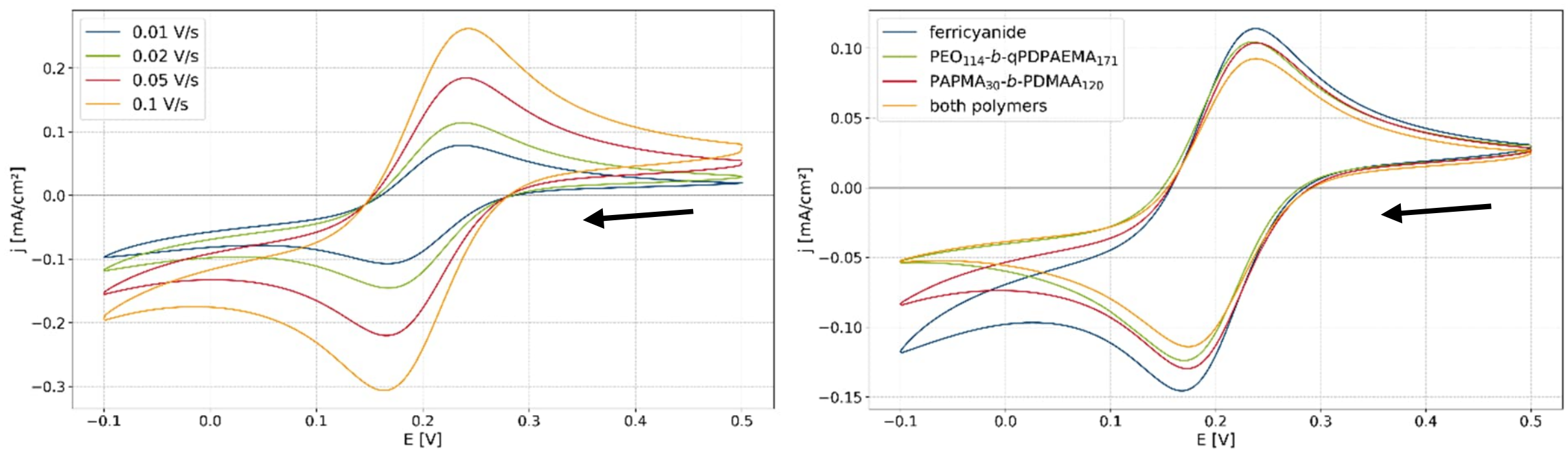

Fig. S6: Cyclovoltammetric results with arrows indicating the scanning direction: (left) for 1.4 mM ferricyanide solution (0.1 M KCl; acetate buffer pH 4.7) for different scan rates; (right) for 1.4 mM ferricyanide solution (0.1 M KCl; acetate buffer pH 4.7) with and without polymer (0.2 g/L PEO_{114}-*b*-$qPDPAEMA_{171}$, 1 g/L $PAPMA_{30}$-*b*-$PDMAA_{120}$ or both) at scan rate 20 mV/s.

Additionally, CVs were conducted for the electrolytically investigated polymer solutions. As shown in Fig. S6, the anodic and cathodic peaks are still rather symmetric for all polymer systems and the peak potentials are not shifted, indicating that there are no adsorption effects at the electrode surface during the electrochemical modulation of the different systems. As expected, the magnitude of the peak currents is lower for the polymer containing solutions, which shows a decreased reduction/oxidation rate compared to the pure ferricyanide due to slower reaction kinetics of the anionic redox probe at the electrode because of the electrostatic interaction with cationic charge of the polymers. In total, the addition of the polymers alters the CVs only slightly.

2.3 Chemical Switching

As a complementary approach to electrochemical switching, the reversibility of the hexacyanoferrate-induced aggregation was tested by chemical oxidation and reduction. This chemical switching was performed by adding approximately 100 µL (2–3 drops from a Pasteur pipette) of either 0.1 M ascorbic acid or 3% hydrogen peroxide as reductant or oxidant, respectively, corresponding to a multifold molar excess over the hexacyanoferrate present. Successful conversion is visually confirmed by the appearance or disappearance of the characteristic yellow color of ferricyanide containing solutions. For both individual polymers, the resulting aggregation states are equivalent to those obtained by direct addition of the respective hexacyanoferrate species, confirming that aggregation is driven by the oxidation state of the iron complex rather than by the chemical reagent itself (Tab. S1). In some cases, a broad peak, probably from loose aggregates, appears in the DLS besides the signal from the unimers (see also sections 2.4 and 2.5). These species were resolved by using regularized fitting methods.

Tab. S1: Chemical oxidation/reduction of ferro-/ferricyanide by addition of 2-3 droplets (approximately 100-150 µL) of either 0.1 M ascorbic acid or 3% hydrogen peroxide, corresponding to a multifold molar excess over the hexacyanoferrate present, with PEO_{114}-*b*-$qPDPAEMA_{171}$ (0.2 g/L) in 0.1 M KCl at pH 4.7 (buffered with sodium acetate). In regularized fitting, loose aggregates also appear for the unimeric state.

$PAPMA_{30}$-*b*-$PDMAA_{120}$			
Condition	Aggregation State	R_h from Cumulant Fit (*PDI*) [nm]	R_h from Regularized Fit [nm]
1.4 mM ferricyanide	dissolved	27.6 ± 0.5 (0.67)	5.8 ± 0.6; 56 ± 11
Reduction (0.1 M Ascorbic Acid)	aggregated	104 ± 1 (0.31)	131 ± 4
1.4 mM ferrocyanide	aggregated	102 ± 2 (0.21)	110 ± 3
Oxidation (3% H_2O_2)	dissolved	41 ± 1 (0.53)	5.3 ± 0.4; 70 ± 3
PEO_{114}-*b*-$qPDPAEMA_{171}$			
Condition	Aggregation State	R_h from Cumulant Fit (*PDI*) [nm]	R_h from Regularized Fit [nm]
1.4 mM ferricyanide	aggregated	34.2 ± 0.2 (0.04)	34.3 ± 0.4
Reduction (0.1 M Ascorbic Acid)	dissolved	9.9 ± 0.5 (0.64)	9.1 ± 0.5; 104 ± 16
1.4 mM ferrocyanide	dissolved	10.0 ± 0.3 (0.55)	9.3 ± 0.5; 108 ± 25
Oxidation (3% H_2O_2)	aggregated	38.1 ± 0.3 (0.10)	39.8 ± 0.8

In the combined system, chemical switching transfers the solution between the two complementary aggregated states, with bimodal size distributions at intermediate conditions reflecting coexistence of dissolved unimers of one polymer alongside aggregates of the other (Tab. S2).

Tab. S2: Chemically induced oxidation/reduction by addition of 2-3 droplets (approximately 100-150 µL) of either 0.1 M ascorbic acid or 3% hydrogen peroxide, corresponding to a multifold molar excess over the hexacyanoferrate present, of the system containing PEO_{114}-*b*-$qPDPAEMA_{171}$ (0.2 g/L) and $PAPMA_{30}$-*b*-$PDMAA_{120}$ (1 g/L) in 0.1 M KCl at pH 4.7. Bimodal distributions in the regularized fit reflect the coexistence of dissolved unimers of PEO_{114}-*b*-$qPDPAEMA_{171}$ and large aggregates of PA PMA_{30}-*b*-$PDMAA_{120}$.

Condition	Species	R_h from Cumulant Fit (*PDI*) [nm]	R_h from Regularized Fit [nm]
1.4 mM ferricyanide	Aggregates of PEO-*b*-qPDPAEMA	40.9 ± 1.1 (0.26)	48 ± 2.7
Reduction (0.1 M Ascorbic Acid)	Aggregates of PAPMA-*b*-PDMAA and unimers of PEO-*b*-qPDPAEMA	75.3 ± 1.0 (0.35)	4.4 ± 0.5; 92.7 ± 3.5
1.4 mM ferrocyanide	Aggregates of PAPMA-*b*-PDMAA and unimers of PEO-*b*-qPDPAEMA	85.8 ± 2.0 (0.39)	5.7 ± 0.8; 104 ± 7.7
Oxidation (3% H_2O_2)	Aggregates of PEO-*b*-qPDPAEMA	39.5 ± 0.5 (0.26)	43.6 ± 1.2

2.4 Exemplary Autocorrelation Functions from DLS-Results

In this section autocorrelation functions at 90° scattering angle with their corresponding fits and decay-time distributions, as fitted by JADE-DLS[4] regularized fitting mode (α = 0.3), are shown for specific states, representatively (Fig. S7).

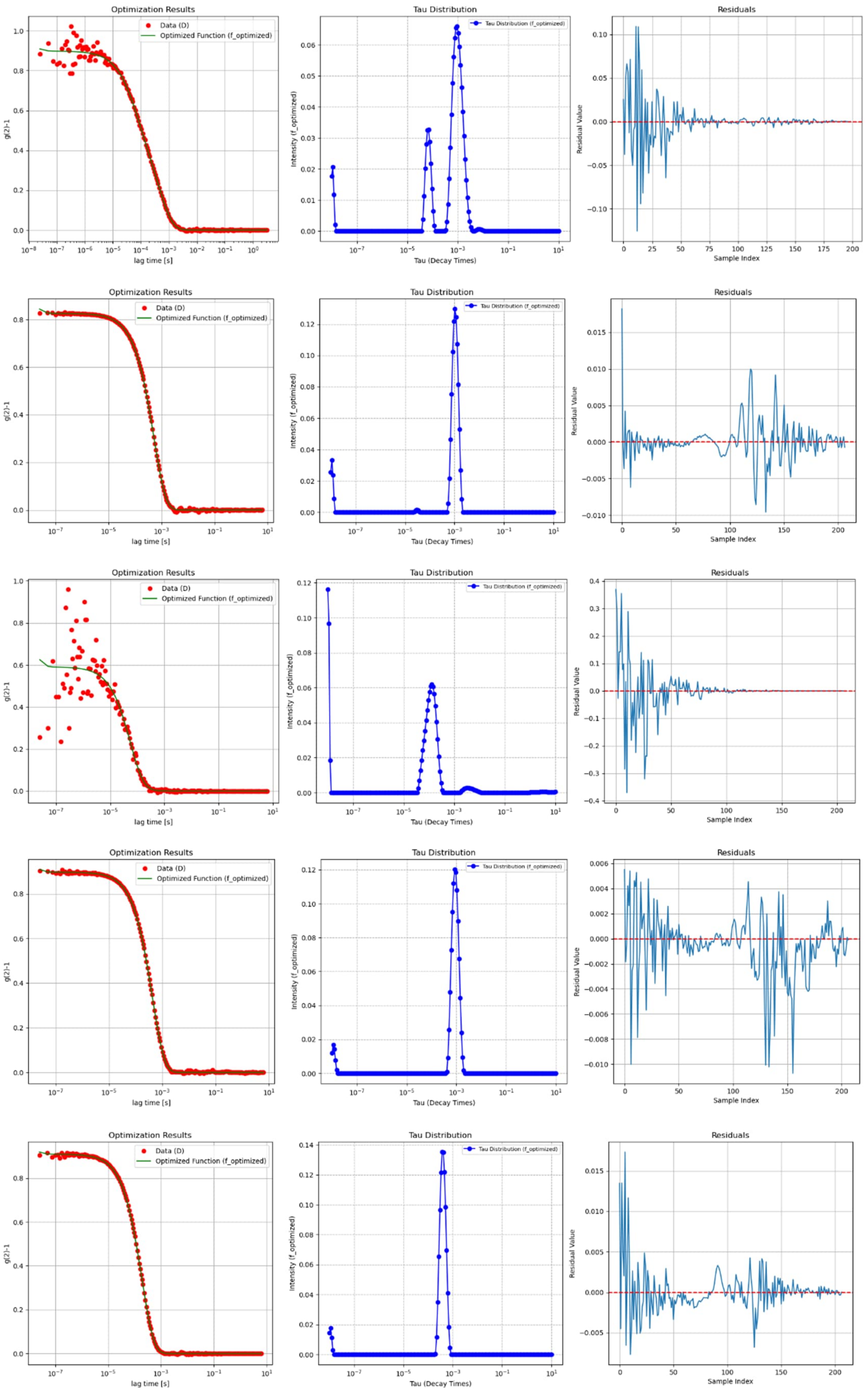

Fig. S7: On the left: autocorrelation functions $g^{(2)}(\tau)$-1 with fits and residuals (right). Center: corresponding decay time distribution to determine the diffusion coefficient by linear regression over multiple angles. From top to bottom: PAPMA$_{30}$-*b*-PDMAA$_{120}$ unimer with loose aggregate; large aggregate of PAPMA$_{30}$-*b*-PDMAA$_{120}$ with a hinted smaller structure present (1.4 mM ferrocyanide); unimer of PEO$_{114}$-*b*-qPDPAEMA$_{171}$

with measurement artifact at the left range of decay times; PEO_{114}-*b*-$qPDPAEMA_{171}$ equilibrium structure (1.4 mM ferricyanide); PEO_{114}-*b*-$qPDPAEMA_{171}$ nonequilibrium aggregate (approx. 1.4 mM ferricyanide). All measurements are taken at 0.1 M KCl and pH 4.7. For PEO_{114}-*b*-$qPDPAEMA_{171}$,the polymer concentration was 0.2 g/L and 1 g/L for $PAPMA_{30}$-*b*-$PDMAA_{120}$.

2.5 Details about Aggregation of $PAPMA_{30}$-*b*-$PDMAA_{120}$ in DLS-Measurements

For DLS measurements for the system containing PAPMA-*b*-PDMAA, additional detail is given in the following section. Addition of small amounts of ferrocyanide to the polymer solution (1 g/L polymer, 0.1 M KCl, pH 4.7 buffered with acetate) does not change the state of the system as compared to the pure polymer solution. PAPMA-*b*-PDMAA is mostly unimolecularly dissolved with some loose aggregates. Starting with a concentration of 0.5 mM, an additional signal can be distinguished in some cases, indicating the formation of small aggregates as seen in cryo-TEM. With increasing concentrations, these aggregates could become more frequent, but would be obscured by larger features in DLS. The DLS method is reaching its limits for these trimodal cases (see Fig. S8) and thus, the results must be treated with caution. Further increase of the iron species concentration leads to the results described in the main section with a major peak and occasional small moieties visible in regularized fitting. As already discussed, a *PDI* threshold of 0.45 was established for this system, defining a *PDI* < 0.45 as "low" dispersity, which is already relatively high compared to usually chosen values and implies significant polydispersity.[8] The chosen value seems reasonable, because the *PDI* defined as "low" dispersity moves in a range of 0.35-0.45, while the *PDI* reaches values of 0.55-0.7 when multiple modes with large errors appear consistently, as it is the case for concentrations lower than 0.7 mM ferrocyanide. This state is then defined here as a "high" dispersity state. When increasing the ferrocyanide concentration even further, an additional effect manifests itself as the decay rate drops with higher angles deviating from linear behavior as depicted in Fig. S8 with the example of a 2 mM ferrocyanide solution. In such a case, the affected angles were excluded from linear regression to still extract usable diffusion coefficients. This kind of deviation can be explained by simple mirroring effects due to the experimental setup to measure these large angles (partial reflection of beam at the opposite side of the cuvette). These findings further demonstrate that only a small concentration window can be used for reliable electrochemical modulation.

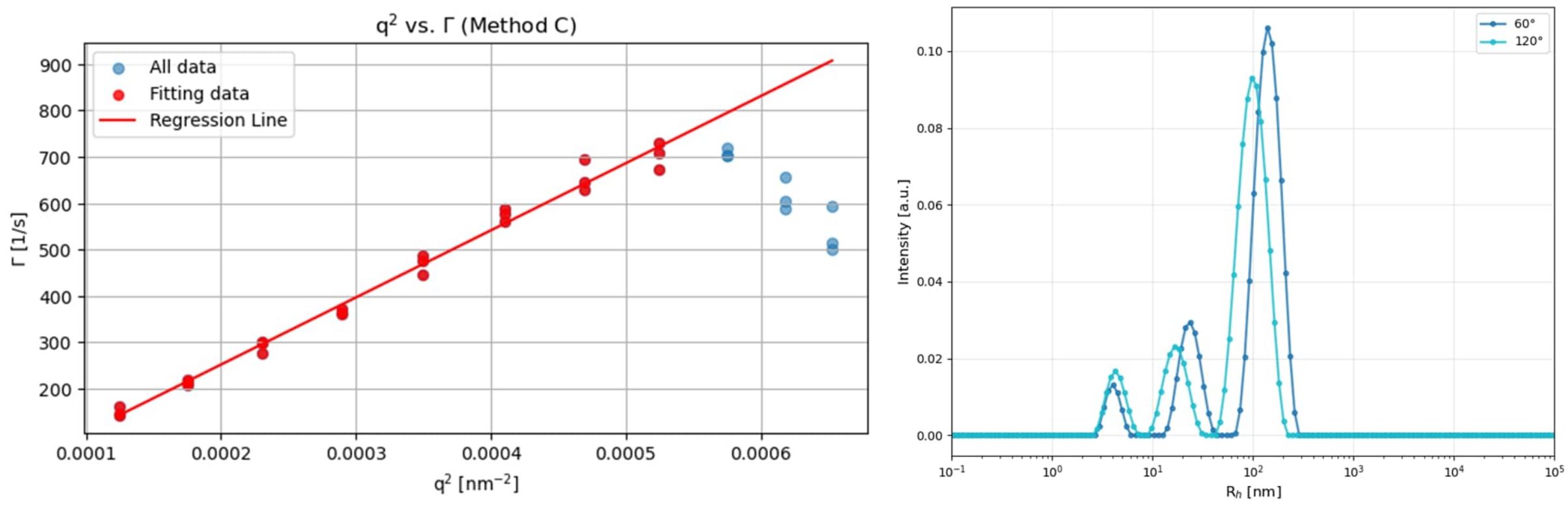

Fig. S8: (left) Decay rates from DLS measurements of PAPMA$_{30}$-*b*-PDMAA$_{120}$ (1 g/L) in 0.1 M KCl at pH 4.7 (buffered with sodium acetate) with 2 mM ferrocyanide for different angles. Larger angles are excluded from the linear regression to determine the diffusion coefficient. (right) Distribution function of DLS measurements of PAPMA$_{30}$-*b*-PDMAA$_{120}$ (1 g/L) in 0.1 M KCl at pH 4.7 with 0.5 mM ferrocyanide at two different angles with a clearly trimodal distribution, indicating the presence of small structures besides unimolecularly dissolved polymer and loose aggregates.

The influence of the salt concentration on the aggregates was investigated by varying the KCl-concentration between 0 and 0.1 M at a ferrocyanide concentration of 1.4 mM. As seen in Tab. S3, the aggregation occurs over the whole concentration range, but the dispersity and aggregate size vary. At zero added KCl, residual positive charge on the aggregates limits the aggregation number, while small additions of salt screen this repulsion, allowing larger but loosely crosslinked aggregates to form via multivalent ferrocyanide bridging. When reaching the concentration of 0.1 M KCl, the Debye screening length is sufficiently compressed to confine each ferrocyanide to tight, short-range ion pairing, yielding smaller and significantly more monodisperse aggregates.

Tab. S3: Salt-concentration dependency of aggregate size of PAPMA$_{30}$-*b*-PDMAA$_{120}$ (1 g/L) with 1.4 mM ferrocyanide (*icr* ≈ 3.3) at pH 4.7 (buffered with sodium acetate).

KCl-concentration [M]	R_h from Cumulant Fit (PDI) [nm]
0	116.5 ± 2.5 (0.33)
0.01	148.1 ± 5.8 (0.27)
0.05	145.6 ± 3.9 (0.32)
0.1	101.6 ± 1.4 (0.20)

2.6 Mixing of the Micellar Aggregates

To investigate the state of mixed hexacyanoferrate composition at which aggregates of both micelles could occur, a mixture of both micellar aggregates was analyzed via DLS as elaborated in the main section. Tab. S4 gives an overview over parameters and results of the experiment.

Tab. S4: Results and parameters of the micellar mixtures. All conditions: 0.1 M KCl, pH 4.66 (sodium acetate buffer). Mixed solution prepared by 1:1 volume mixing of the two individual solutions leading to approximately 21% ferricyanide. (*icr* = initial charge ratio).

Parameter	PEO_{114}-*b*-$qPDPAEMA_{171}$	Mixed (50/50)	$PAPMA_{30}$-*b*-$PDMAA_{120}$
Polymer conc. [g/L]	0.4	0.2 / 1.0	2.0
Ferricyanide [mM]	0.6	0.3	—
Ferrocyanide [mM]	—	1.1	2.2
Ferricyanide fraction [%]	100	ca. 21	0
icr (ferrocyanide)	1.8	—[a]	2.2
icr (ferricyanide)	1.3	—[a]	1.7
R_h cumulant (*PDI*) [nm]	39.3 ± 0.4 (0.06)	47.0 ± 0.5 (0.28)	141 ± 4 (0.12)
R_h regularized [nm]	38 ± 1	51.6 ± 2.6	148 ± 10

[a] *The icr is not calculated for the mixed system due to the simultaneous presence of two polymers with different charge densities and selectivities.*

2.7 Combined Polymer System at high pH and Full Electrochemical Control

During the bulk electrolysis experiments, by applying a potential of -1 V, the pH can be effectively increased due to water electrolysis even for the buffered system. As mentioned, this procedure was applied to a solution shown in Fig. 14 (main section), where both polymer systems are combined, which results in conditions of pH 11.2 with completely reduced ferricyanide. With these conditions, there should be no aggregation at all and indeed it is

possible to distinguish between both polymers in their unimeric state and loose aggregates as illustrated in Fig. S9. Again, the dominance of loose aggregates/premicelles in dynamic light scattering (intensity-weighted) does not mean that the major component by mass consists of loose aggregates, but the small, weakly scattering unimers constitute most likely the major fraction in the system. This is also outlined by a drop of the scattering intensity from around 0.3 to 0.1 (average intensity at 90° scattering angle; in a.u.) with the mentioned pH increase. As reported in the main section, the combined system was subjected to a complete cycle of triggers to demonstrate a full four-state electrochemical control. More detailed results are provided in Table S5. Representatively, the R_h resulting from cumulant fitting are shown. Generally, the results are subject to high *PDI*, which is attributed to dust contamination, as revealed by spurious large-size populations in the regularized fit. The qualitative state discrimination is nonetheless preserved throughout, starting with the nonequilibrium micelles of PEO-*b*-qPDPAEMA that dissolve in favor of the PAPMA-*b*-PDMAA aggregates with increasing ferrocyanide concentration. The unimolecular state at step 2 is reflected in a large increase in *PDI* to 0.6 and significant drop in intensity, consistent with the absence of well-defined aggregates and the dominance of scattering from dust particles. As soon as the ferrocyanide is oxidized again, the equilibrium micelles of PEO-*b*-qPDPAEMA are formed that remain stable upon subsequent re-acidification to pH 4.4. The smaller aggregate-size in step 3 is explainable, because the redox potential in solution remains shifted toward the reduced state following the electrochemical pH increase, resulting in incomplete re-oxidation and consequently smaller equilibrium micelles. From the equilibrium state we can return to aggregates of PAPMA-*b*-PDMAA. Hence, we could demonstrate a full control of the micellization state of complex polymer mixtures by merely electrochemical means.

Tab. S5: Demonstrating full electrochemical control over all four accessible aggregation states of the combined polymer system (0.2 g/L PEO_{114}-*b*-$qPDPAEMA_{171}$ and 1 g/L $PAPMA_{30}$-*b*-$PDMAA_{120}$ in 0.1 M KCl) through modulation of both the hexacyanoferrate redox ratio and the solution pH.

Step	Operation	pH	Dominating Fraction	R_h from Cumulant Fit (*PDI*) [nm]	*I* [kHz][a]
0	Start with 1.4 mM ferricyanide	4.7	nonequilibrium micelles of PEO_{114}-*b*-$qPDPAEMA_{171}$	44.2 ± 1.2 (0.42)	0.791 ± 0.005
1	Reduction (-0.1 V)	4.7	Aggregates of $PAPMA_{30}$-*b*-$PDMAA_{120}$	186 ± 4 (0.33)	0.406 ± 0.011
2	Reduction (-1 V)	11.1	unimers/loose aggregates	119 ± 6 (0.60)	0.125 ± 0.004
3	Oxidation (0.5 V)	10.5	equilibrium micelles of PEO_{114}-*b*-$qPDPAEMA_{171}$	53.2 ± 1 (0.42)	0.835 ± 0.028
4	Oxidation (1.4 V)	4.4	equilibrium micelles of PEO_{114}-*b*-$qPDPAEMA_{171}$	66.8 ± 1.1 (0.46)	1.088 ± 0.060
5	Reduction (-0.1 V)	4.5	aggregates of $PAPMA_{30}$-*b*-$PDMAA_{120}$	118 ± 5 (0.43)	0.213 ± 0.004

[a] *average scattering intensity at 90° scattering angle*

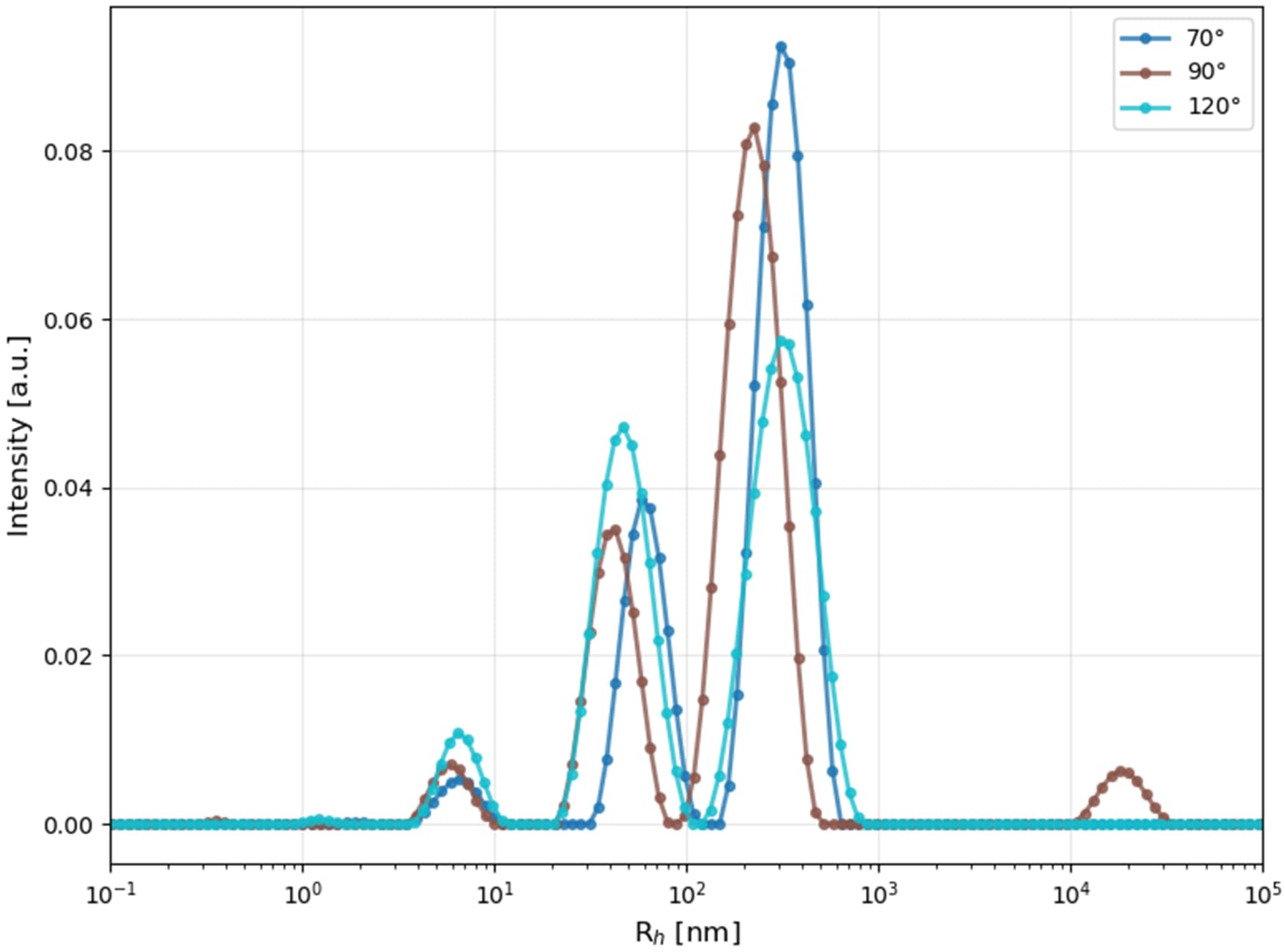

Fig. S9: Trimodal distribution at different scattering angles found at a pH of 11.2 with PEO_{114}-*b*-q$PDPAEMA_{171}$ (0.2 g/L) and $PAPMA_{30}$-*b*-$PDMAA_{120}$ (1 g/L) in 0.1 M KCl after electrochemically reducing a 1.4 mM ferricyanide solution. From left to right the three main peaks are most likely unimers of $PAPMA_{30}$-*b*-$PDMAA_{120}$ overlapping with the unimers of PEO_{114}-*b*-q$PDPAEMA_{171}$, premicellar structures and loose aggregates.

2.8 Cryo-TEM Details and Comparative Measurements

Comparative measurements demonstrated that the aggregates observed in cryo-TEM are specifically induced by the respective iron species interacting with each polymer. Fig. S10 shows the image of a PAPMA-*b*-PDMAA containing solution (1 g/L polymer, 0.1 M KCl, acetate buffer pH 4.7) without (Fig. S10A) and with ferrocyanide (Fig. S10B,D,E,F), whereas it becomes clear that visible structures appear because of the aggregation of the polymer with ferrocyanide. Further confirmation of the larger structures was also found by investigation of PAPMA-*b*-PDMAA containing solution with identical parameters without the KCl (comparable results for R_h: 117 nm for saltless solution versus 102 nm for 0.1M KCl; details see section 2.3 above). Several larger aggregates can be viewed here in thicker parts of the vitrified water film near the edges of the grid (Fig. S10C). Such size segregation, showing the larger structures to the edge, is commonly observed in cryo-TEM. The details about the size determination discussed in the main section is shown in Fig. S10E and F. In Fig. S10D, an occasionally found elongated worm-like structure is shown.

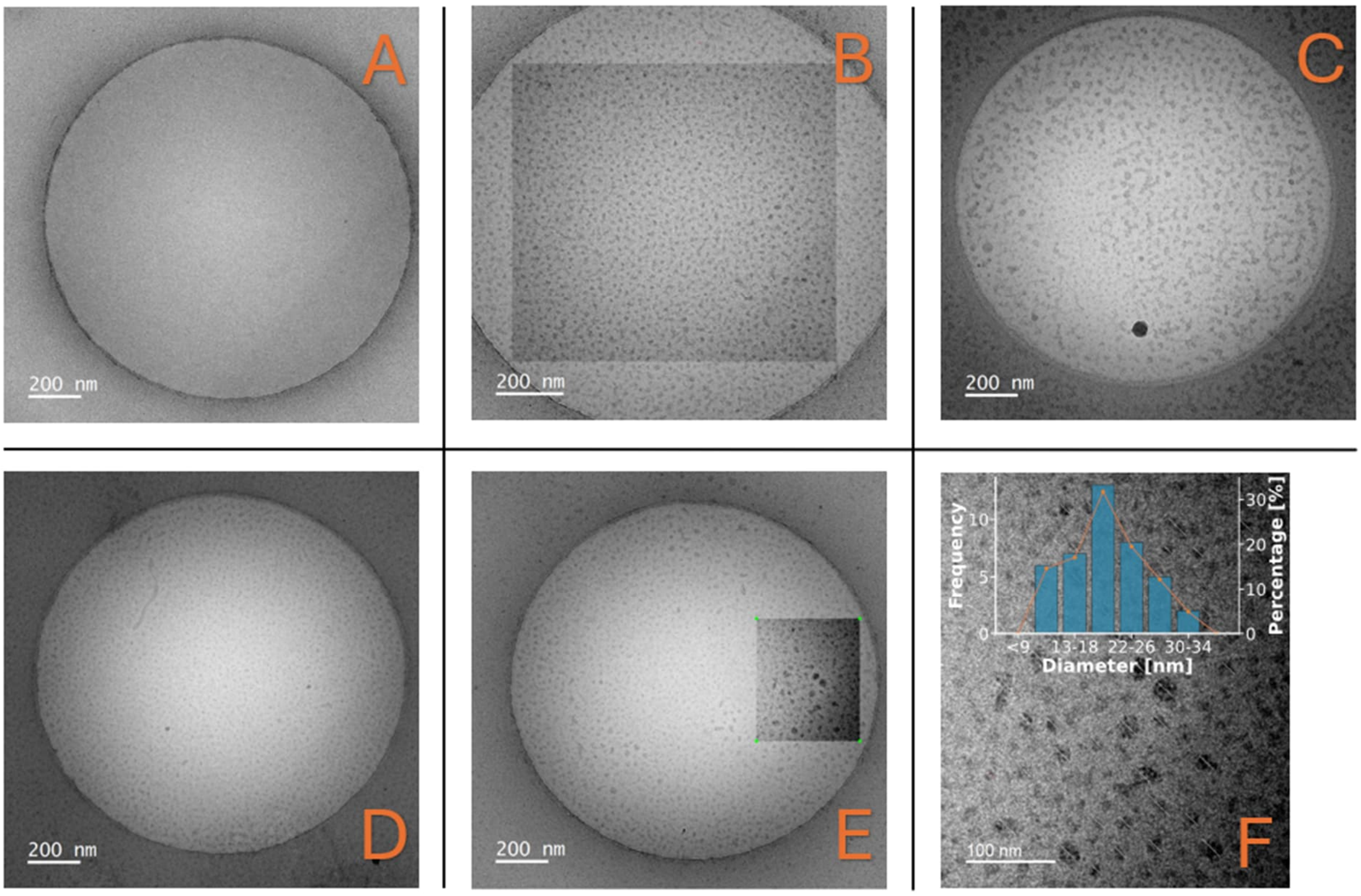

Fig. S10: Cryo-TEM images of $PAPMA_{30}$-*b*-$PDMAA_{120}$ aggregation behavior. (A) Polymer solution (1 g/L polymer; 0.1 M KCl; acetate buffer pH 4.7) showing uniform contrast consistent with unimolecularly dissolved polymer chains. (B) Addition of 1.4 mM ferrocyanide leads to visible aggregation behavior (marked area is used in main section). (C) Polymer solution (1 g/L polymer; acetate buffer pH 4.7) with addition of 1.4 mM ferrocyanide without 0.1 M KCl. (D) Polymer aggregates with occasional wormlike feature. (E) and (F) showing the snippet used for size determination.

The same comparison is done for PEO-*b*-qPDPAEMA (0.2 g/L polymer; 0.1 M KCl; acetate buffer pH 4.7). Fig. S11A depicts the polymer solution without ferricyanide showing uniform contrast consistent with unimolecularly dissolved polymer chains, while with the addition of 1.4 mM ferricyanide micelles become visible. Besides the prominent large structures, dendritic patterns are observed on the samples containing ferricyanide, exemplary shown in Fig. S11B and E. This pattern formation is also visible in a control experiment using the same solution without polymer (Fig. S11C). This suggests that these patterns arise from ferricyanide containing solution, rather than from polymer aggregation. Here, ferricyanide can interact with the copper to produce materials of Prussian blue analogues.[9]

It cannot be excluded that the slightly anionic surface of the carbon film (due to glow charge transfer treatment) leads to an additional interaction with the cationic moieties of the polymers, but skipping of that surface treatment leads to insufficient wetting of the grid making the imaging process impossible.

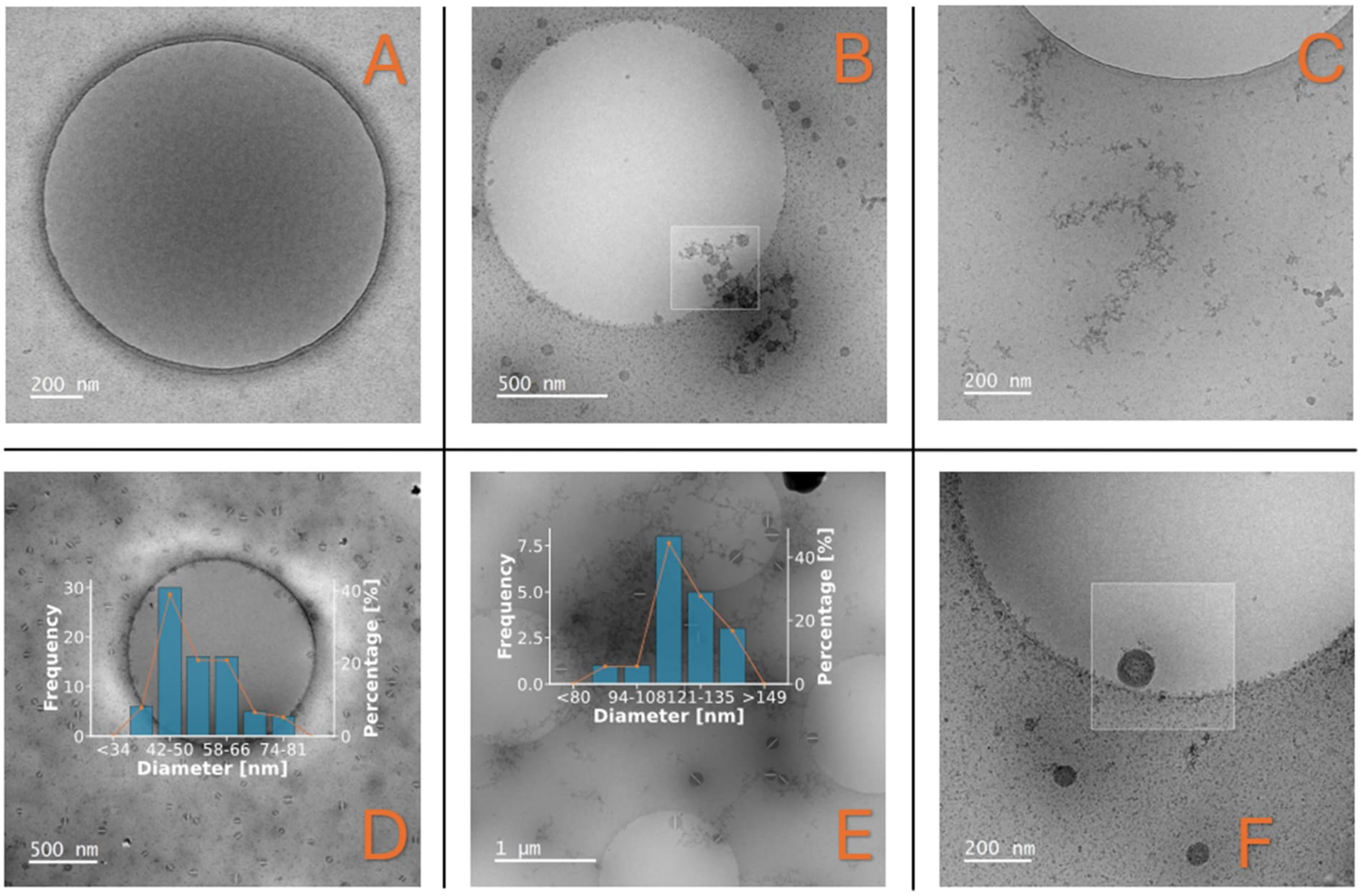

Fig. S11: Cryo-TEM images of PEO_{114}-*b*-$qPDPAEMA_{171}$ aggregation behavior. (A) Polymer solution (0.2 g/L polymer; 0.1 M KCl; acetate buffer pH 4.7) showing uniform contrast consistent with unimolecularly dissolved polymer chains. (B) Addition of 1.4 mM ferricyanide leads to visible aggregation behavior (nonequilibrium micelles). (C) Images of polymer-free 1.4 mM ferricyanide solution (0.1 M KCl; acetate buffer pH 4.7) showing dendritic structures. (D) Size determination of nonequilibrium micelles. In (E) and (F) the electrochemically assembled equilibrium structures are depicted, whereas in (E) the size determination is shown and (F) depicts from where the zoom-in picture of the single micelle shown in the main section is taken from.

In another experiment, it was tried to further limit influencing factors by using a gold instead of a copper grid. While no changes can be observed for PAPMA-*b*-PDMAA (Fig. S12A), PEO-*b*-qPDPAEMA now exhibits flatted structures (Fig. S12B), which most likely result from interfacial rearrangements as described in the literature before[10].

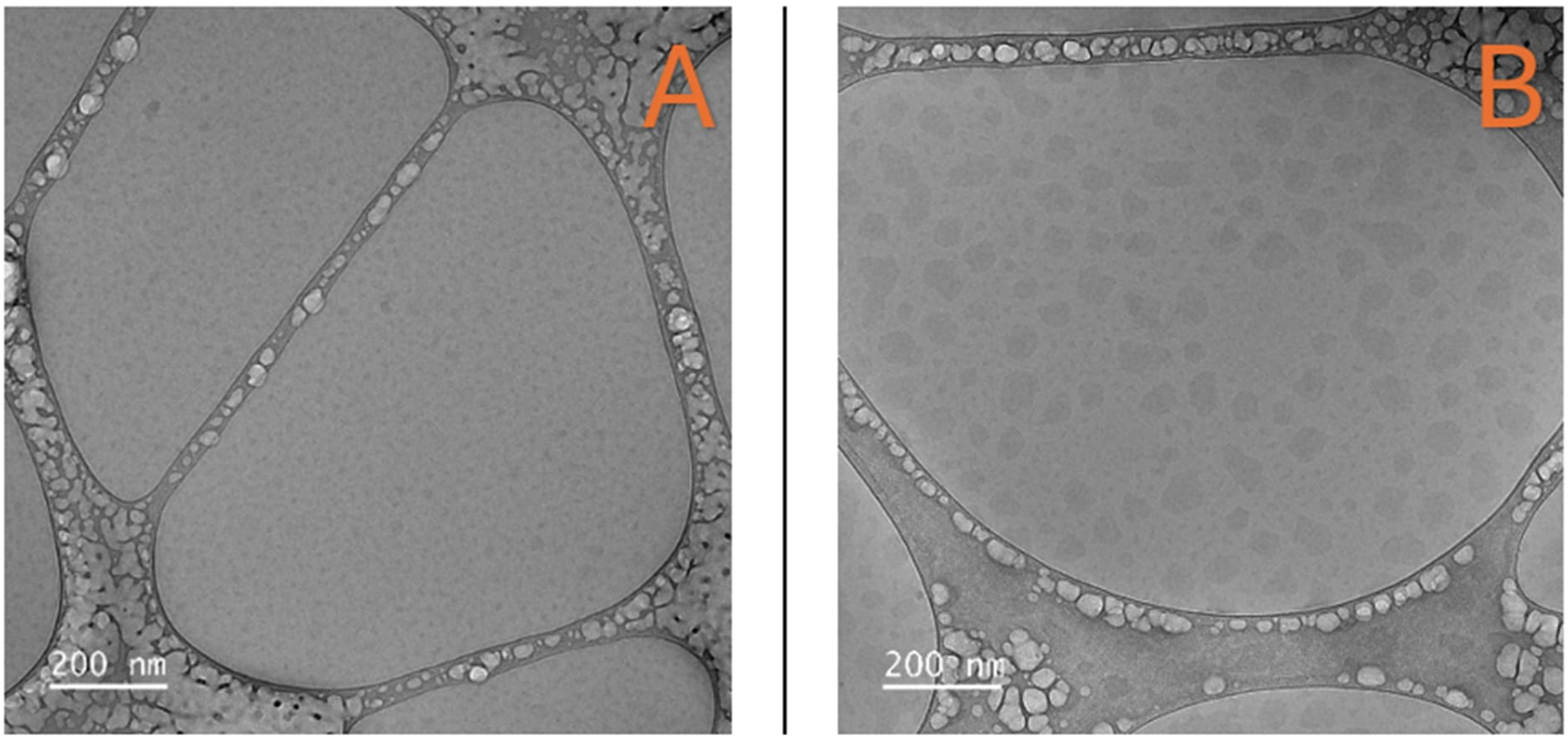

Fig. S12: Cryo-TEM images of hexacyanoferrate containing polymer solutions on Au support grid. (A) showing PAPMA$_{30}$-*b*-PDMAA$_{120}$ solution (1 g/L polymer; 0.1 M KCl; acetate buffer pH 4.7; 1.4 mM ferrocyanide) and (B) PEO$_{114}$-*b*-qPDPAEMA$_{171}$ solution (0.2 g/L polymer; 0.1 M KCl; acetate buffer pH 4.7; 1.4 mM ferricyanide).

Cryo-TEM of the combined system was attempted, but the images do not give a clear picture of the structures present as self-sorting into two distinct micelle populations is a possible interpretation as well as mixed aggregates (Fig.S13). This ambiguity is inherent to the system in the absence of polymer-specific labeling strategies which would be required for definitive differentiation, representing a possible strategy for future work. The rather monomodal DLS size distribution nevertheless remains consistent with co-assembly into mixed aggregates rather than two independently scattering populations.

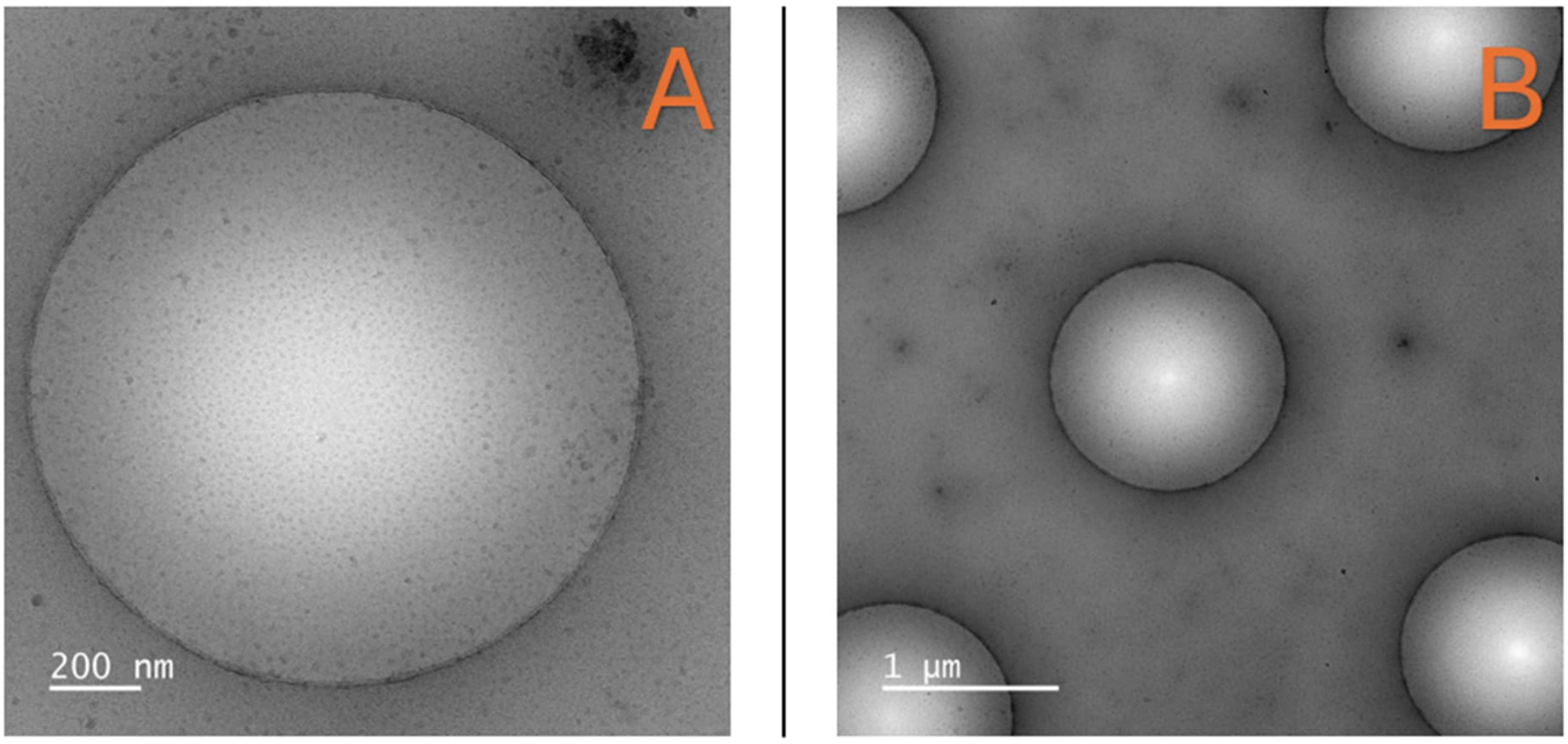

Fig. S13: Cryo-TEM images of the combined system at 21% ferricyanide (0.3 mM ferricyanide + 1.1 mM ferrocyanide, 0.2 g/L PEO-*b*-qPDPAEMA + 1 g/L PAPMA-*b*-PDMAA) reveal small irregular aggregates reminiscent of those observed for pure PAPMA-*b*-PDMAA in the presence of ferrocyanide (compare Fig. 5). Notably, the compact spherical nonequilibrium micelles characteristic of pure PEO-*b*-qPDPAEMA

are absent. This could indicate a combination of co-assembly of both polymers into mixed aggregates and small PAPMA-*b*-PDMAA aggregates as shown before.

2.9 Electrochemical Switching of PEO_{114}-*b*-$qPDPAEMA_{171}$

In general ferrocyanide solutions are sensitive to oxygen and light, leading to oxidation of the ferrocyanide.[11] Thus, all ferrocyanide-containing solutions are freshly prepared and the solutions are purged with nitrogen during the electrochemical experiments. To measure DLS during the electrochemical experiments, the sample comes in contact with oxygen, which is only a problem while investigating PEO_{114}-*b*-$qPDPAEMA_{171}$. During the first electrochemical reduction (Fig. 9), subsequent DLS measurements indicate an aggregation with sizes up to almost 350 nm after leaving the sample unattended for only a few minutes. This behavior is in line with the other results for little higher ferricyanide concentrations described before. By filling the sample vial with the solution while purging with nitrogen, this effect could be minimized and it was possible to characterize the unimolecular state. The fact that the vesicles achieved with the electrochemical pathway are more stable than the original crew-cut micelles is underlined by the observation that an instant growth of micelles up to 350 nm after several minutes is not observed in the vesicle case.

2.10 Details about SAXS Measurements from PEO_{114}-*b*-$qPDPAEMA_{171}$

This section gives details about the fitting of the SAXS-data presented in the main section. Initially, the approximate scattering densities (*sld*) were calculated, resulting in $9.4 \cdot 10^{-6}$ 1/Å^{-2} for the buffered (pH 4.7) 0.1 M KCl solution and $10.5 \cdot 10^{-6}$ 1/Å^{-2} for the polymer/ferricyanide complex. It was assumed that the ferricyanide is completely incorporated into the micellar structure and the qPDPAEMA is densely packed. The background was fitted first. Tab. S1 lists the detailed fit parameters for the SAXS-fits shown in the main section. Cryo-TEM suggested that the resulting micelles were spherical. Consequently for both micellar morphologies, the spherical core-shell formfactor was used in which the scale, background and solvent *sld* was fixed. The *sld* for core and shell were allowed to be fitted between 9.4 and $10.5 \cdot 10^{-6}$ 1/Å^{-2} and as mentioned in the main section it became clear that the *sld* of the core dominated over the *sld* of the shell for the nonequilibrium micelles and vice versa for the equilibrium micelles, suggesting that simpler models are appropriate as well. Thus, a spherical form factor was chosen for the former, resulting in a radius of approximately 25 nm. The *sld* was again allowed to vary in the given range. The resulting *sld* is lower than the calculated value, which is probable because the qPDPAEMA might be stretched and swollen with some solvent. The polydispersity

was constrained between 0.01 and 0.2, which was deduced from the *PDI* of DLS. For the vesicle model, the shell volume fraction (vol. fraction parameter) was fixed at 1.0, assuming a densely packed, non-swollen PDMAEMA-ferricyanide membrane, while the polydispersity was constrained between 0.1 and 0.45 (also deduced from the *PDI* by DLS). Nevertheless, a total radius of approximately 40 nm was obtained. For both models the scale was allowed to vary during the fit process, but kept in the same range for comparability. The low scale indicates a lower effective polymer concentration, which is most likely originating in uncomplexed ferricyanide in the solution, resulting in a reduced contrast between micelles and solvent.

Tab. S1: Fitting parameters for SAXS results shown in Fig. 11 in the main section. For the nonequilibrium state, the sphere, and for the equilibrium state, the hollow sphere formfactor is chosen (fit function see section 1.3.3).

Parameter	*Sphere*	*Hollow Sphere*
Scale	*0.017*	*0.021*
Background [cm^{-1}]	*0.2*	*0.4*
sld [10^{-6}/$Å^{-2}$]	*10.38*	*10.45*
sld solvent [10^{-6}/$Å^{-2}$]	*9.4*	*9.4*
Polydispersity	*0.05*	*0.4*
Radius [Å]	*254.72*	*400.63*
Vol.fraction	-	*1*
Shell Thickness [Å]	-	*225.57*

2.11 SAXS Measurements at Higher Concentrations

In addition to the SAXS measurements discussed in the main section, higher concentrated samples were investigated whether PAPMA-*b*-PDMAA aggregates would exhibit enhanced scattering signals at the ESRF.[12,13] As illustrated in Fig. S14, samples at concentrations of 25 g/L and 50 g/L with proportionally scaled ferrocyanide concentration display scattering intensities comparable to those of the 0.1 M KCl reference, indicating no significant improvement. Only at large *q*, there is an upturn in intensity being probably linked to a polymer form factor from the unimers, which is most likely the result of the very high polymer concentration used. On the other hand, increasing the concentration of PEO-*b*-qPDPAEMA accordingly leads to enhanced scattering at intermediate *q*. The curves for 5 g/L and 10 g/L PEO-*b*-qPDPAEMA are fitted using the sphere form factor (Fig. S15) yielding similar radii (about 26 nm) as for the 0.2 g/L obtained in the main section. Several reasons could contribute to these observations. First, the complexation equilibrium could change with PAPMA-*b*-PDMAA concentration, shifting also the critical ferrocyanide contents for micellization. In case of such a weak complexation, this interdependence also makes a *cmc*-determination difficult, though we are surely beyond the *cmc*s in the aggregated states. In addition, the stimuli-responsive PAPMA block is considerably shorter than the qPDPAEMA block, expecting smaller micelles for PAPMA-*b*-

PDMAA than for PEO-*b*-qPDPAEMA with lower aggregation number. Though DLS of PAPMA-*b*-PDMAA (at 1 g/L) shows rather large aggregates of about 100 nm or even larger (whose main X-ray scattering is expected in a range outside our accessible *q*-range), some part of the polymer could be still assembled into smaller entities. At 1 g/L, their X-ray scattering might be too low to resolve the aggregates of PAPMA-*b*-PDMAA. In contrast, cryo-TEM indicates such small structures and sometimes larger structures. The increase in size in Fig. 4 could be also a consequence of the presence of some vesicles or an elongation toward cylindrical micelles, while still spherical micelles (or even unimers) could be a considerable fraction. The hydrophobic dodecyl-endgroup from the CTA could contribute to the partial formation of such worm-like/vesicular micelles, where the endgroup could probably bend back to the core of the micelles, leading to an increased hydrophobic/hydrophilic balance in favor of some higher order morphology micelles. Hence, the PEO-*b*-qPDPAEMA micellization in presence of ferricyanide is very robust and can be scaled to different concentrations. Such a facile treatment cannot be seen for PAPMA-*b*-PDMAA and ferrocyanide, where the equilibrium could shift and the concentrations of the partners need to be adjusted to other concentrations. These results further demonstrate that in a modulated system containing both polymers, the concentration of PAPMA-*b*-PDMAA is a critical factor. In line with the negligible scattering in SAXS at switching conditions, the interaction between PAPMA and ferrocyanide seems weaker than the one between ferricyanide and qPDPAEMA, which could be partly caused by the different block ratios of the two bishydrophilic polymers. However, previous studies[14] indicated also such a weak binding scenario in case of the polyelectrolytes without a nonionic block component (also homopolymers). Hence, the PAPMA/ferrocyanide interaction is weaker compared to the strong polyelectrolyte couple, irrespective of the fully charged state of PAPMA at low pH.

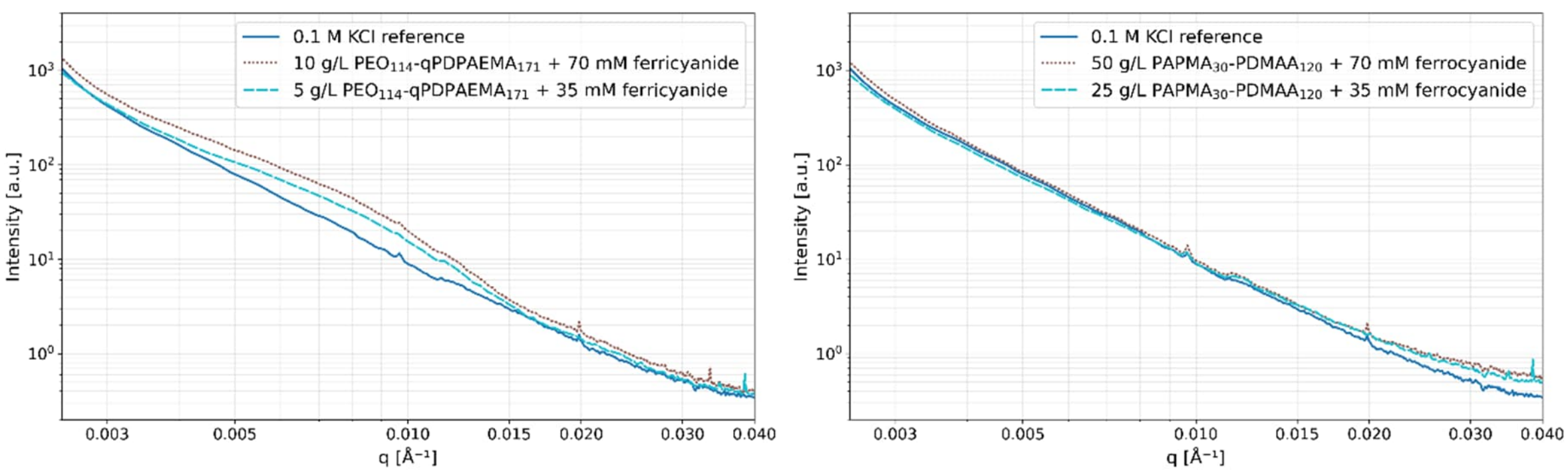

Fig. S14: SAXS raw measurements of the investigated polymers at higher concentrations. For $PAPMA_{30}$-*b*-$PDMAA_{120}$ no significant improvement of the scattering signal is visible, while the scattering intensity of PEO_{114}-*b*-$qPDPAEMA_{171}$ scales with increasing concentrations.

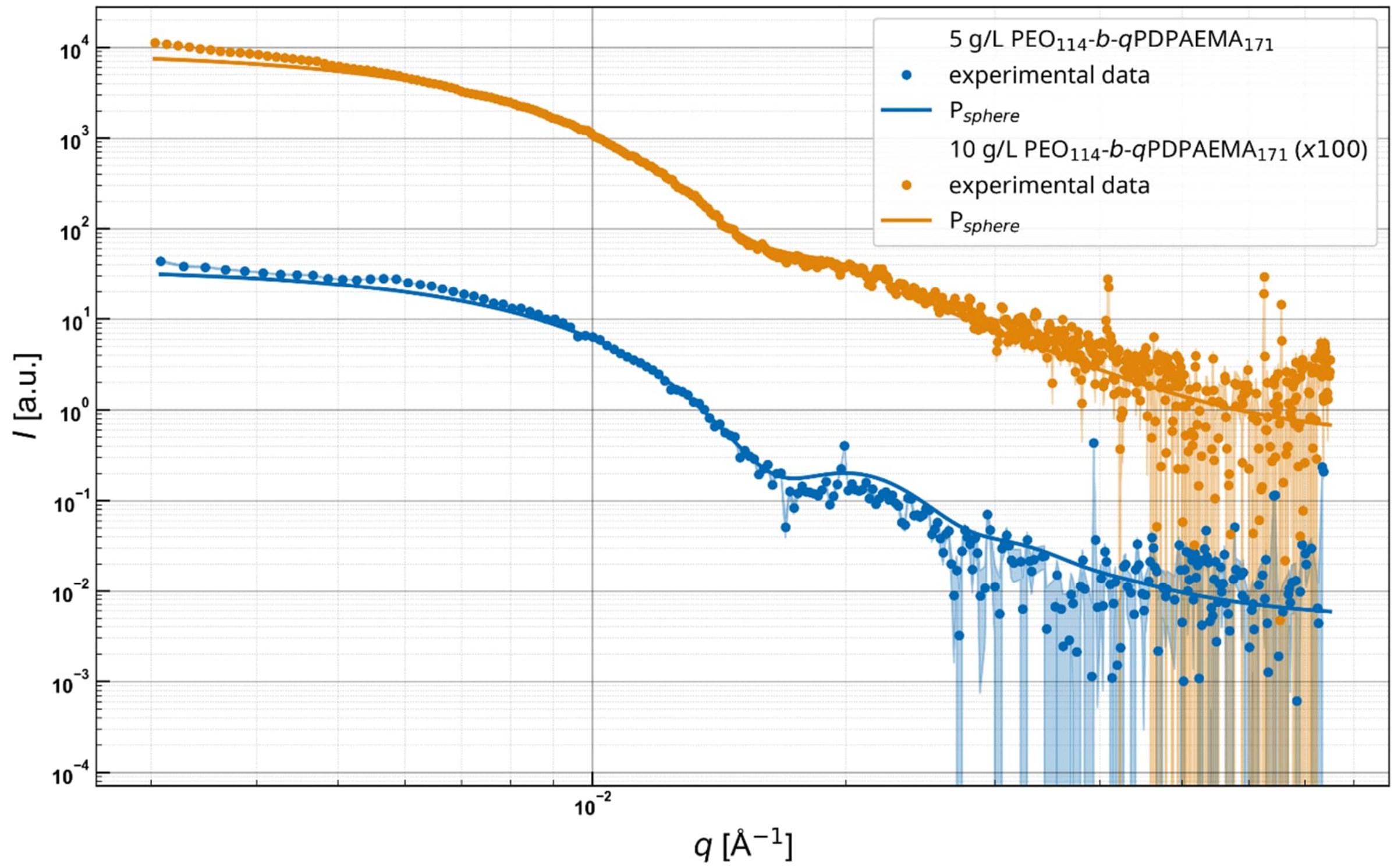

Fig. S15: In blue: PEO$_{114}$-*b*-qPDPAEMA$_{171}$ (5 g/L polymer, 0.1 M KCl, acetate buffer pH 4.7). In orange: PEO$_{114}$-*b*-qPDPAEMA$_{171}$ (10 g/L polymer, 0.1 M KCl, acetate buffer pH 4.7). Both scattering curves are fitted using the sphere form factor resulting in a radius of approximately 26 nm (fit parameter in Tab. S2).

Tab. 2: Fitting parameters for SAXS results shown in Fig. S14. Both are fitted with the sphere form factor (fit function see section 1.3.3).

Parameter	*5 g/L*	*10 g/L*
Scale	*1*	*1*
Background [cm^{-1}]	*0.005*	*0.005*
Sld [10^{-6}/Å^{-2}]	*9.33*	*9.31*
Sld solvent [10^{-6}/Å^{-2}]	*9.4*	*9.4*
Polydispersity	*0.14*	*0.2*
Radius [Å]	*259.16*	*257.58*

3. REFERENCES

(1) Fulmer, G. R.; Miller, A. J. M.; Sherden, N. H.; Gottlieb, H. E.; Nudelman, A.; Stoltz, B. M.; Bercaw, J. E.; Goldberg, K. I. NMR Chemical Shifts of Trace Impurities: Common Laboratory Solvents, Organics, and Gases in Deuterated Solvents Relevant to the Organometallic Chemist. *Organometallics* **2010**, *29* (9), 2176–2179. DOI: 10.1021/om100106e.

(2) Doucet, M. e. a. *SasView*, 6.1.0. DOI: 10.5281/zenodo.15775666.

(3) A. Guinier and G. Fournet. *Small-Angle Scattering of X-Rays*; John Wiley and Sons, 1955.

(4) Schildknecht, V.; Neubert, R. *JADE-DLS*, 2025, 1.0. https://github.com/AG-Plamper/JADE-DLS. DOI: 10.5281/zenodo.16841738.

(5) G. Moad and E. Rizzardo, Ed. *RAFT Polymerization:Methods, Synthesis and Applications*, 2021.

(6) Keddie, D. J.; Moad, G.; Rizzardo, E.; Thang, S. H. RAFT Agent Design and Synthesis. *Macromolecules* **2012**, *45* (13), 5321–5342. DOI: 10.1021/ma300410v.

(7) Vasilieva, Y. A.; Scales, C. W.; Thomas, D. B.; Ezell, R. G.; Lowe, A. B.; Ayres, N.; McCormick, C. L. Controlled/living polymerization of methacrylamide in aqueous media via the RAFT process. *Journal of Polymer Science Part A: Polymer Chemistry* **2005**, *43* (14), 3141–3152. DOI: 10.1002/pola.20786.

(8) Filippov, S. K.; Khusnutdinov, R.; Murmiliuk, A.; Inam, W.; Zakharova, L. Y.; Zhang, H.; Khutoryanskiy, V. V. Dynamic light scattering and transmission electron microscopy in drug delivery: a roadmap for correct characterization of nanoparticles and interpretation of results. *Materials Horizons* **2023**, *10* (12), 5354–5370. DOI: 10.1039/D3MH00717K.

(9) Yun, J.; Kim, Y.; Gao, C.; Kim, M.; Lee, J. Y.; Lee, C.-H.; Bae, T.-H.; Lee, S. W. Copper Hexacyanoferrate Thin Film Deposition and Its Application to a New Method for Diffusion Coefficient Measurement. *Nanomaterials* **2021**, *11* (7), 1860. DOI: 10.3390/nano11071860.

(10) Prasser, Q.; Steinbach, D.; Münch, A. S.; Neubert, R.; Weber, C.; Uhlmann, P.; Mertens, F.; Plamper, F. A. Interfacial Rearrangements of Block Copolymer Micelles Toward Gelled Liquid–Liquid Interfaces with Adjustable Viscoelasticity. *Small* **2022**, *18* (18), 2106956. DOI: 10.1002/smll.202106956.

(11) Enomoto, T.; Tsuji, Y.; Li, X.; Akimoto, A. M.; Yoshida, R. Molecular Insights into Photochemical Hydrogen Evolution Dual Catalysis within the Shell of Polymer Micelle-Based Nanoreactors. *ACS Appl. Nano Mater.* **2023**, *6* (12), 10078–10086. DOI: 10.1021/acsanm.3c00621.

(12) Chahine, G. A.; Blanc, N.; Arnaud, S.; Geuser, F. de; Guinebretière, R.; Boudet, N. Advanced Non-Destructive in Situ Characterization of Metals with the French Collaborating Research Group D2AM/BM02 Beamline at the European Synchrotron Radiation Facility. *Metals* **2019**, *9* (3), 352. DOI: 10.3390/met9030352.

(13) Neubert, R.; Rosenfeldt, S.; Schildknecht, V.; Voigt, A. *ASAXS on flower-like micelles with iron core [Dataset]. European Synchrotron Radiation Facility.*, 2028. DOI: 10.15151/ESRF-ES-2049617513.

(14) Schneider, S.; Janssen, C.; Klindtworth, E.; Mergel, O.; Möller, M.; Plamper, F. Influence of Polycation Composition on Electrochemical Film Formation. *Polymers* **2018**, *10* (4). DOI: 10.3390/polym10040429. Published Online: Apr. 12, 2018.
